# Synergistic effects of melatonin and 24-epibrassinolide on chickpea water deficit tolerance

**DOI:** 10.1186/s12870-024-05380-2

**Published:** 2024-07-15

**Authors:** Matin Abdoli, Mohamad Reza Amerian, Mostafa Heidari, Amin Ebrahimi

**Affiliations:** Agronomy and Plant Breeding Department, Faculty of Agriculture, Shahrood University of Technology, Semnan, Iran

**Keywords:** Chickpea, Melatonin, 24-epibrassinolide, Biochemical and physiological characteristics, Water deficit conditions

## Abstract

**Background:**

Water deficiency stress reduces yield in grain legumes, primarily due to a decrease in the pods number. Melatonin (ML) and 24-epibrassinolide (EBL) are recognized for their hormone-like properties that improve plant tolerance to abiotic stresses. This study aimed to assess the impact of different concentrations of ML (0, 100, and 200 µM) and EBL (0, 3, and 6 µM) on the growth, biochemical, and physiological characteristics of chickpea plants under water-stressed conditions.

**Results:**

The study's findings indicated that under water-stressed conditions, a decrease in seed (30%) and pod numbers (31%), 100-seed weight (17%), total chlorophyll content (46%), stomatal conductance (33%), as well as an increase in H_2_O_2_ (62%), malondialdehyde content (40%), and electrolyte leakage index (40%), resulted in a 40% reduction in chickpea plants grain yield. Our findings confirmed that under water-stressed conditions, seed oil, seed oil yield, and seed protein yield dropped by 20%, 55%, and 36%, respectively. The concurrent exogenous application of ML and EBL significantly reduces oxidative stress, plasma membrane damage, and reactive oxygen species (ROS) content. This treatment also leads to increased yield and its components, higher pigment content, enhanced oil and protein yield, and improved enzymatic and non-enzymatic antioxidant activities such as catalase, superoxide dismutase, polyphenol oxidase, ascorbate peroxidase, guaiacol peroxidase, flavonoid, and carotenoid. Furthermore, it promotes the accumulation of osmoprotectants such as proline, total soluble protein, and sugars.

**Conclusions:**

Our study found that ML and EBL act synergistically to regulate plant growth, photosynthesis, osmoprotectants accumulation, antioxidant defense systems, and maintain ROS homeostasis, thereby mitigating the adverse effects of water deficit conditions. ML and EBL are key regulatory network components in stressful conditions, with significant potential for future research and practical applications. The regulation metabolic pathways of ML and EBL in water-stressed remains unknown. As a result, future research should aim to elucidate the molecular mechanisms by employing genome editing, RNA sequencing, microarray, transcriptomic, proteomic, and metabolomic analyses to identify the mechanisms involved in plant responses to exogenous ML and EBL under water deficit conditions. Furthermore, the economical applications of synthetic ML and EBL could be an interesting strategy for improving plant tolerance.

**Supplementary Information:**

The online version contains supplementary material available at 10.1186/s12870-024-05380-2.

## Background

Significant global expansion is observed in the environmental stressors affecting agricultural products, such as drought [1], salinity [2], abnormal temperatures [3], microplastic [4, 5], and heavy metal pollutions [6–9]. These findings have raised concerns and directed research attention to this area [10]. Drought stress reduces plant growth by affecting the plant's physiological and biochemical processes, including enzyme activity, cell membrane permeability, leaf water status, and rate of photosynthesis. Due to the complex interplay between environmental conditions and the many physiological, biochemical, and molecular processes that affect plant growth and development, plants have a complicated resistance to drought stress [10, 11].

The Mediterranean region, South Asia, and North Africa are crucial areas for the growth of chickpeas (*Cicer arietinum*), which rank as the second most significant and lucrative legume in the world [12]. It is recognized as one of the most noteworthy legumes, set apart by its large quantities of calcium and phosphorus and its high digestible protein content. Within chickpea cultivation regions worldwide, water deficit conditions during various growth stages are recognized as a fundamental and impactful challenge, resulting in decreased grain yield. The erratic rainfall patterns significantly hamper chickpea productivity, resulting in documented losses of up to 50% [13]. Acquiring sufficient knowledge about the responses of plants to environmental stresses is essential for improving the productivity of breeding programs targeted at producing drought-tolerant cultivars [13, 14]. Developing cultivars resistant to and adapted to environmental stresses through traditional breeding methods or biotechnology is time-consuming and expensive. Consequently, scientists are constantly looking for quick, easy, and environmentally sustainable alternatives [13, 14]. Several abiotic and biotic factors, such as fungi and bacteria [6], hormones or growth regulators [15], nanomaterials, biochar [16], and cold plasma [17], have been recognized as effective approaches for developing resilient and adaptable plant varieties to environmental stresses [1–3, 18].

Phytohormones, natural organic compounds, wield substantial influence over pivotal plant life cycle processes even at low concentrations. They participate in signal transduction networks, which enhance plant development and productivity in response to abiotic stresses [19]. Melatonin (ML) and brassinosteroids (BRs) are key elicitors that regulate various physiological processes in plants, especially in response to abiotic stresses. Through mechanisms such as enhancing growth, minimizing oxidative damage, and fine-regulating stress-responsive genes, BRs, and ML may counteract the detrimental effects of stress and fortify plant survival in adverse environmental conditions [20–23].

(BRs) play a crucial role in regulating gene transcription, which encodes proteins and enzymes essential for coping with various stressors. Despite their diminished activity under normal plant growth conditions, BRs are notably advantageous in stress conditions. These benefits include the reactive oxygen species (ROS) removal, maintenance of redox balance, increased carbon absorption, photoprotection, and increased antioxidant capacity through both enzymatic and non-enzymatic ROS scavenger mechanisms. Applying exogenous BRs has emerged as a valuable strategy, enhancing plants' ability to withstand abiotc stresses [21, 22]. Exogenous 24-epibrassinolide (EBL) enhances important processes like maximum quantum efficiency, net photosynthetic rate, transpiration rate, stomatal conductance, and chlorophyll content. Specifically, applying EBL to wheat leaves under high-temperature conditions boosts biomass accumulation, growth, photosynthetic efficiency, and antioxidant capacity [24].

In response to stress, the expression of genes involved in ML biosynthesis increases, leading to a surge in endogenous ML. This enhances physiological aspects such as boosting the antioxidant system to mitigate oxidative damage, resulting in reduced accumulation of ROS and reactive nitrogen species (RNS), decreased electrolyte leakage (EL), lowered lipid peroxidation, diminished relative conductivity, reduction in toxic substance levels, limitation of cellular redox imbalance, and improved nitro-oxidative balance [15, 25]. It is now established that applying ML and EBL improves plants' ability to withstand heat [3], cold [26], salt [2, 23], heavy metals [27], and biotic stresses, in addition to improving their resistance to drought stress [1]. These compounds also prolong the freshness of cut flowers [28] and increase the shelf life, and quality of fruits and vegetables [29].

Encountering water deficit conditions during reproductive and grain-filling phases negatively impacts legumes, often leading to notable yield decreases; therefore, it's crucial to enhance chickpea yield and evaluate its response under such conditions [13, 14]. In recent years, ML and EBL have attracted attention from researchers due to their unique ability to confer resistance to abiotic stresses in plants [1–3, 15]. Although the individual impacts of EBL and ML in enhancing plant tolerance to various abiotic stresses are well-established, their combined effects in mitigating the impact of stressors remain unexplored. This study investigates the impact of EBL and ML on growth, photosynthesis, biochemical traits, and oxidative stress markers in *Cicer arietinum* under water deficit conditions. The findings of this study will be instrumental in pinpointing the traits most affected by water deficit conditions or the application of ML and EBL in chickpea plants.

## Results and discussion

### Analysis of Variance

The variance analysis indicates that water deficit conditions significantly affected all traits, including yield, its components, as well as physiological, biochemical, and nutritional characteristics. Additionally, aside from plant height (PH), number of lateral branches (NLB), pod weight per plant (PWP), main branch diameter (MBD), percentage of seedless pods (PSP), chlorophyll b content (Chl b), ascorbate peroxidase (APX), guaiacol peroxidase (GPX), hydrogen peroxide (H_2_O_2_), and stomatal conductance (SC), all other traits investigated in this study exhibited significant variations in response to simultaneous application of ML and EBL. Furthermore, the experiment, conducted over two separate crop years yielded noteworthy alterations in all parameters at 1% or 5% significance levels (Supplementary Table 1). The double interaction effects of the treatments, including year*EBR, year*ML, various levels of irrigation*year, different levels of irrigation*EBR, diverse levels of irrigation*MEL, and ML*EBR, were found to be statistically significant for some of the investigated traits at significance levels of 1 or 5%. The combined triple interaction effects of the different irrigation levels, ML, and EBR resulted in fewer differences in the assessed attributes (Supplementary Table 1).

### Comparative analysis of yield and its components traits affected by experimental treatments

**Fig. 1:**
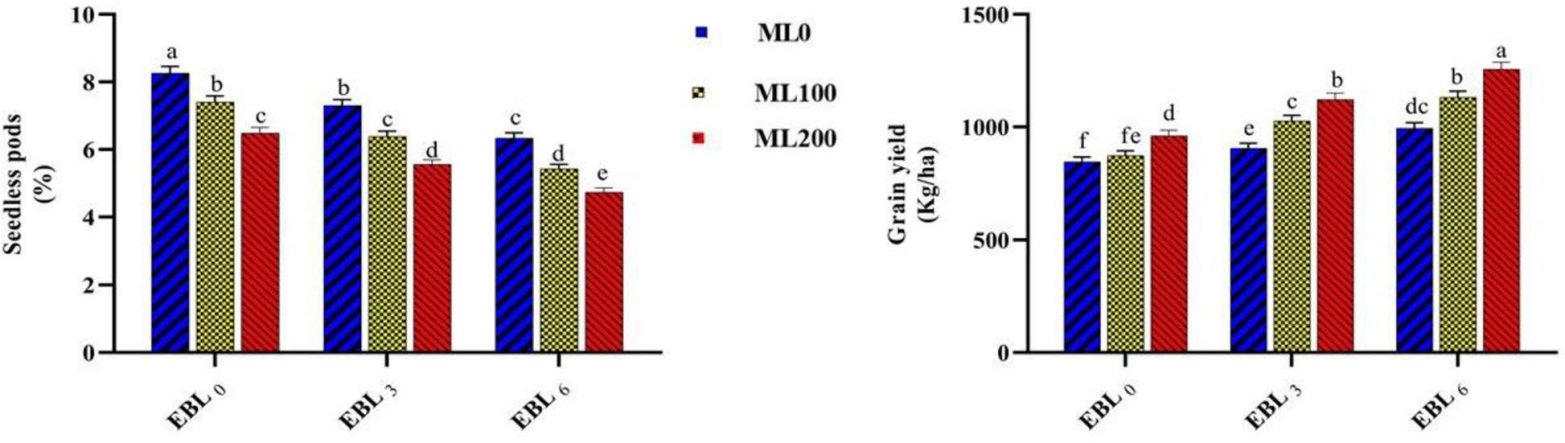
Illustrating the effects of various melatonin (ML) (0, 100, and 200 µM), and 24-epibrassinolide (EBL) (0, 3, and 6 µM) levels on the grain yield, and seedless pods (%). Duncan's test was utilized to compare means at a 1% confidence level, indicating that columns sharing identical letters are not statistically distinct

Our study's conclusions revealed that water deficit conditions significantly reduced chickpea yield and its components over both crop years. In terms of PH, number of main branches (NMB), NLB, number of seeds per plant (NSP), number of pods per plant (NPP), PWP, 100-seed weight (W100S) and pod (W100P), MBD, and grain yield (GY), it led to reductions of 8.5%, 20%, 11%, 30%, 31%, 39%, 17%, 14%, 20%, and 40%, respectively (Supplementary Table 3). Employing varying levels of EBL and ML individually and concurrently effectively mitigated the adverse impacts of water deficit conditions on these characteristics (Supplementary Tables 4, 5, and 11). Specifically, the simultaneous application of 6 µM EBL and 200 µM ML significantly boosted traits such as NMB, NSP, NPP, W100S, W100P, and GY by 45%, 30%, 30%, 17%, 19%, and 40%, respectively, compared to their absence. The highest values of GY (1256.56 kg/ha), W100S (39.52 g), NPP (44.55), NSP (61.90), W100P (45.22 g), and NMB (3.39) were recorded in the combined treatment, whereas the lowest of GY (846.05 kg/ha), W100S (33.99 g), NPP (34.30), NSP (48.10), W100P (38.55 g), and NMB (2.71) were observed when no elicitors were applied (Supplementary Table 11). Investigating the interaction effects of different irrigation levels *EBL on the GY and PSP revealed notable alterations in these traits influenced by the treatments. The highest and lowest values of GY (1454.65 kg/ha) and PSP (4.97%) were observed with the utilization of EBL (200 µM) and ML (6 µM) under normal irrigation conditions. Conversely, the lowest and highest values of these traits were 619.02 kg/ha and 8.50%, respectively, under water stress conditions without these elicitors' application. Certainly, applying these elicitors under water deficit conditions led to a 30% rise in GY and a 23% decline in PSP traits compared to the absence of EBL (Fig. 1). Chickpeas are often grown in rotation within farming systems in many regions, relying on residual soil moisture. Consequently, the crop experiences stress during its vegetative and reproductive growth stages, leading to significant yield losses [30]. This study attributes the noticeable decline in grain yield to the detrimental effects of water deficit conditions on traits such as NPP, NSP, PWP, W100S, and W100P. Additionally, the PSP increased, likely due to the adverse effects of water deficit conditions on flower formation and development, ultimately contributing significantly to the reduction in grain yield. In previous studies, the reduction in legume grain yield under drought stress has mainly been attributed to a decrease in pod number [20, 31], aligning with the findings of this study. Drought stress during the flowering phase primarily reduces chickpea yield by causing flower shedding and restricting pod development. Despite its importance, research concerning the effects of drought stress during the flowering stage in chickpeas is limited [32]. Moreover, this study highlights the significant contribution of water deficit conditions to a decline in specific vegetative traits such as PH, NMB, NLB, straw and stubble yield (SSY), and MBD. The decrease in these traits, coupled with NPP, NSP, PWP, W100S, and W100P, seems to have significantly diminished GY. Hussain et al. emphasized the adverse impact of water deficit during the flowering stage, leading to decreased pod length and size, ultimately affecting the plant's reproductive structures [33]. MT and EBL treatments have been demonstrated to stimulate germination, growth, biosynthesis of primary and secondary metabolites, maturation, and productivity of crops, along with overall plant development under exposure to abiotic stresses [1, 23, 34–36]. Similar findings were reported by Gholami et al., who observed increased water deficit tolerance in cowpea plants following the exogenous application of EBL [1].

During abiotic stresses, ROS accumulate in the tapetum, triggering premature degeneration and resulting in the production of abnormal or infertile pollen grains [37]. Pre-treatment with ML has been demonstrated to reduce pollen damage by decreasing ROS accumulation in anthers [38]. The findings of this investigation indicate that water deficit conditions significantly decreased chickpea seed yield through various mechanisms, with a key factor being the occurrence of seedless pods. Application of EBL and ML, either individually or combined, notably decreased the occurrence of seedless pods under both normal and stress conditions compared to not utilizing these elicitors. The effects of applying exogenous ML and EBL are comparable to genetically modified plants with upregulated biosynthetic genes for ML and EBL under abiotic stresses. However, in comparison to genetically engineered crops, the exogenous application of ML and EBL offers a more time-efficient and cost-effective approach to enhancing plant resistance to abiotic stresses [25, 39, 40].

### Comparative analysis of physiological, and biochemical traits affected by experimental treatments

Our research findings demonstrated a significant decrease of 43%, 55%, 46%, 40%, 37%, and 33% in the contents of chlorophyll a (Chl a), Chl b, total chlorophyll (Tot chl), carotenoid (Cart), relative water content (RWC), and stomatal conductance (SC) under water deficit conditions compared to the normal irrigation conditions. On the contrary, water deficit conditions led to an increase of 32%, 29%, 54%, 30%, 32%, 39%, 40%, 40%, 62%, 45%, 50%, 56%, and 51% in catalase (CAT), polyphenol oxidase (PPO), superoxide dismutase (SOD), APX, GPX, total soluble protein (So pro), malondialdehyde (MDA), EL, H_2_O_2_, proline (Prol), flavonoid (Flavo), phenol (Phe), and sugars (Sug) compared to the normal irrigation conditions (Supplementary Table 3).

**Fig. 2:**
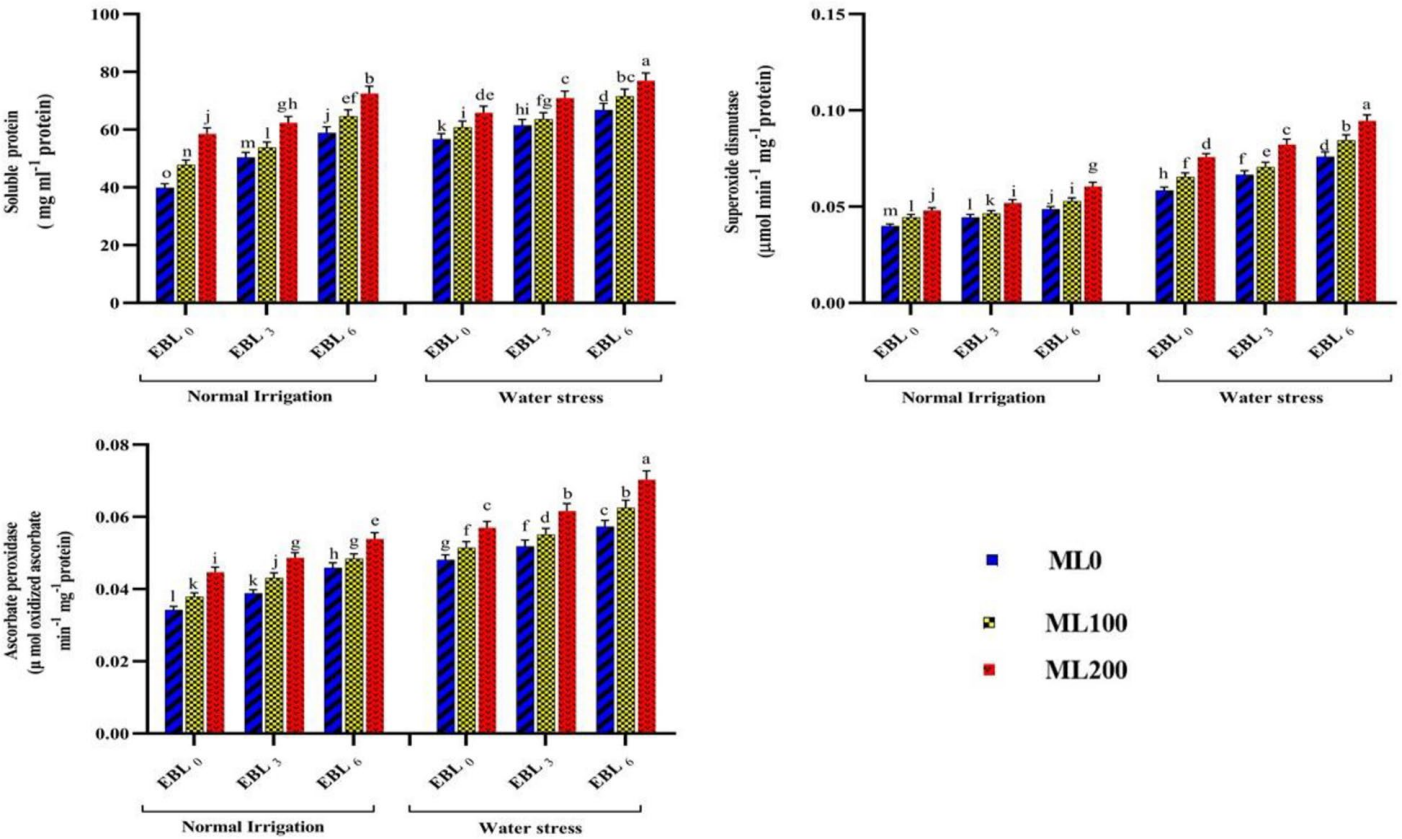
Illustrating the effects of different melatonin (ML) (0, 100, and 200 µM), and 24-epibrassinolide (EBL) (0, 3, and 6 µM) levels on total soluble protein and the activities of superoxide dismutase, and ascorbate peroxidase. Duncan's test was utilized to compare means at a 1% confidence level, indicating that columns sharing identical letters are not statistically distinct

**Fig. 3:**
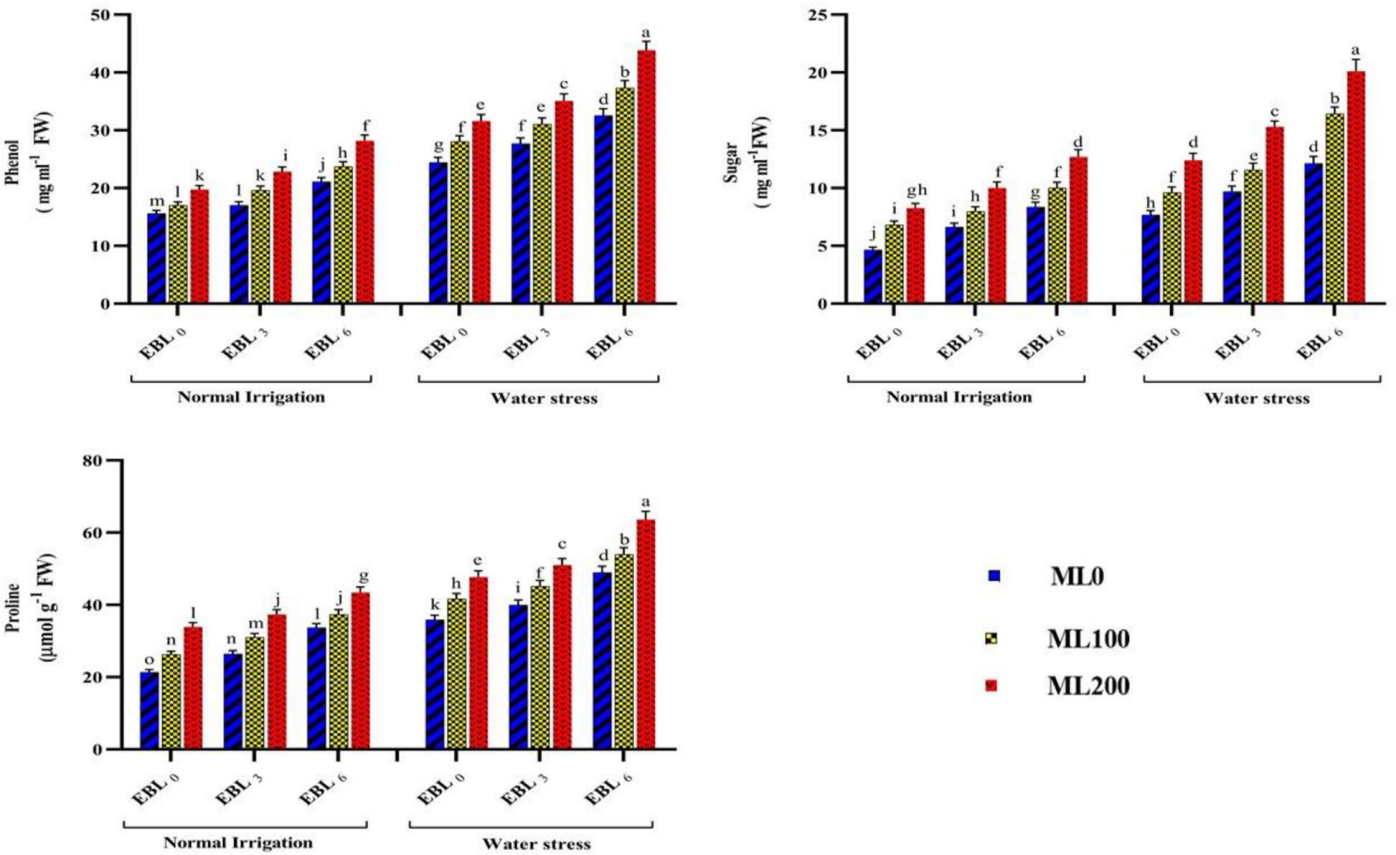
Illustrating the effects of different melatonin (ML) (0, 100, and 200 µM), and 24-epibrassinolide (EBL) (0, 3, and 6 µM) levels on the contents of proline, phenol, and sugars. Duncan's test was utilized to compare means at a 1% confidence level, indicating that columns sharing identical letters are not statistically distinct

Adding 6 µM of EBL and 200 µM of ML to chickpea plants increased the contents of Chl a, Tot chl, Cart, RWC, CAT, PPO, SOD, So pro, Prol, Flavo, Phe, and Sug by 47%, 49%, 47%, 22%, 63%, 31%, 57%, 44%, 80%, 115%, 80%, and 150%, respectively. On the contrary, the simultaneous application of these two elicitors led to a decrease in MDA (38%), H_2_O_2_ (34%), and EL (34%), compared to not utilizing them. The concurrent application of EBL and ML yielded the highest levels of Chl a (9.86 mg g^−1^ FW), Tot chl (13.96 mg g^−1^ FW), Cart (6.82 mg g^−1^ FW), RWC (68.72%), CAT (0.059 μmol of H_2_O_2_ decomposed min^−1^ mg^−1^ protein), PPO (0.080 μmol min^−1^ mg^−1^ protein), SOD (0.077 μmol min^−1^ mg^−1^ protein), So pro (74.73 mg ml^−1^ protein), Prol (53.53 μmol g^−1^ FW), Flavo (27.85 mg g^−1^ FW), Phe (36.03 mg g^−1^ FW), and Sug (16.40 mg g^−1^ FW) traits. Without utilizing these two elicitors, the lowest values for these attributes were 6.72, 9.36, 4.62, 57.08, 0.036, 0.058, 0.049, 48.29, 28.63, 12.60, 20.02, and 6.21, respectively. Furthermore, the group that did not receive EBL and ML treatments exhibited the highest levels of MDA (9.49 μmol g^−1^ FW), EL (43.23%), and H_2_O_2_ (10.73 μmol g^−1^ FW). In contrast, the simultaneous application of these two elicitors resulted in the lowest values for these traits (6.30, 25.79, and 7.30, respectively) (Table 1). Examining the combined impacts of varying irrigation, EBL, and ML levels on several physiological and biochemical parameters revealed considerable alterations in these treatment-impacted features. When EBL at level 3 (6 µM) and ML at level 3 (200 µM) were simultaneously applied under water deficit conditions, the highest values of SOD (0.094 μmol min^−1^ mg^−1^ protein), APX (0.070 μmol oxidized ascorbate min^−1^ mg^−1^ protein), Sopro (76.95 mg ml^−1^ protein), Prol (63.64 μmol g^−1^ FW), Phe (43.86 mg ml^−1^ FW), and Sug (20.12 mg g^−1^ FW) were observed. Conversely, under normal water conditions without the utilization of these two elicitors, the attributes reached their minimum values. However, under water deficit stress and treatment with level 3 EBL and ML, chickpea plants exhibited significant increases of 62%, 46%, 40%, 75%, 75%, and 280% in these traits compared to untreated conditions (Figs. 2 and 3). Elevated levels of ROS during abiotic stresses may cause considerable chlorophyll breakdown [30]. The current study found a significant decrease in photosynthetic pigment content, SC, and RWC in chickpea plants under water deficiency conditions. However, applying ML and EBL treatments resulted in the enhancement of these factors. Our current investigation found that exogenous EBL and ML treatments significantly boosted chlorophyll a and b synthesis, most likely due to increased expression of the CmCHLP and CmLHC genes, which encode proteins involved in chlorophyll biosynthesis [41, 42].

**Table 1:** The comparing mean interaction effects of varying levels of melatonin (ML) and 24-epibrassinolide (EBL) on evaluated traits of chickpea plants

**Treatment**		**Chl a**	**Tot chl**	**Cart**	**MDA**	**EL**	**PPO**	**CAT**	**SOD**	**So pro**	**Prol**	**Flavo**	**Phe**	**Sug**	**H**_**2**_**O**_**2**_	**RWC**
**EBL**	**ML**															
**0**	**0**	6.72 e	9.36 e	4.62 e	9.49 a	43.23 a	0.05835 h	0.0360 e	0.0492 g	48.29 h	28.63 h	12.60 g	20.02 g	6.21 g	10.73 a	57.08 f
	**1**	7.44 d	10.26 d	5.09 d	8.52 b b	39.10 b	0.06226 g	0.0404 d	0.0551 f	54.30 g	33.98 f	15.77 f	22.54 f	8.22 f	10.05 b	58.86 e
	**2**	8.03 c	11.18 c	5.530 c	7.38 c	32.94 de	0.06728 e	0.0462 c	0.0620 d	62.22 d	40.83 d	19.17 d	25.69 e	10.33 d	9.21 c	61.21 d
**1**	**0**	7.49 d	10.54 d	5.04 c	8.19 b	37.54 c	0.06241 g	0.0403 d	0.0555 f	55.89 f	33.19 g	15.75 f	22.37 f	8.16 f	9.84 b	59.39 e
	**1**	8.09 c	11.39	5.532 c	7.62 c	33.81 d	0.06571 f	0.0452 c	0.5860 e	58.74 e	38.10 e	17.83 e	25.35 e	9.76 e	9.14 c	62.86 c
	**2**	9.10 b	12.77 b	6.16 b	6.90 d	28.92 f	0.07221 c	0.0518 b	0.0671 c	66.65 c	44.21 c	22.18 c	28.96 c	12.70 c	8.32 d	65.23 b
**2**	**0**	8.19 c	11.44 c	5.46 c	7.32 c	32.50 e	0.06806 d	0.0459 c	0.0623 d	62.85 d	41.32 d	19.50 d	26.84 d	10.44 d	9.02 c	61.53 d
	**1**	9.23 b	12.94 b	5.99 b	6.90 d	29.30 f	0.07346 b	0.0525 b	0.0687 b	68.08 b	45.64 b	23.00 b	30.52 b	13.21 b	8.23 d	65.35 b
	**2**	9.86 a	13.96 a	6.82 a	6.30 e	25.79 g	0.08091 a	0.0590 a	0.0776 a	74.73 a	53.53 a	27.85 a	36.03 a	16.40 a	7.30 e	68.72 a

Columns sharing alike letters showed no significant differences

*Chl a* Chlorophyll a, *Tot chl* Total chlorophyll, *Cart* Carotenoid, *MDA* Malondialdehyde, *EL* Electrolyte leakage, *CAT* Catalase, *SOD* Superoxide dismutase, *PPO* Polyphenol oxidase, *So pro* Total soluble protein, *Prol* Proline, *Flavo* Flavonoid, *Phe* Phenol, *Sug* Sugar, *RWC* Relative water content, *H*_*2*_*O*_*2*_ Hydrogen peroxide

Previous studies have demonstrated that ML and EBL prevent chlorophyll from breaking under stress conditions by either boosting de novo chlorophyll synthesis or inhibiting chlorophyll degradation [42, 43]. They also modulate chemical components required for enzymatic activity in carbon dioxide fixation, such as rubisco [44, 45]. Abiotic stress-induced photoinhibition is lessened by the ML and EBL treatments, which also increase photosystem II efficiency, Calvin cycle enzyme performance, CO_2_ assimilation, and stomatal conductance in leaves [46, 47]. ML regulates aquaporin channels to enhance root water absorption, aiding stress tolerance, and prioritizes water status maintenance by increasing stomatal conductance, improving cell turgor and water retention, and regulating stomatal opening [48, 49]. Key life processes such as the maximum quantum efficiency of PSII, net photosynthetic rate, transpiration rate, stomatal conductance, and chlorophyll content are enhanced by the applications of EBL and ML [24, 47]. EBL has been proven to mitigate the adverse impacts of salt stress on *Solanum tuberosum* L. by increasing the levels of photosynthetic pigments, photosynthetic electron transport, and the maximum and effective quantum yields of photosystem II [50]. The study's findings indicated that employing ML and EBL enhanced SC, aiding the plant in regulating its water status during water scarcity. This enhancement in stomatal function and pigment retention led to higher photosynthetic efficiency. Previous reports have also highlighted similar improvements in *Cucumis sativus* and *Triticum aestivum* L. following EBL application under environmental stresses [51, 52]. Additionally, ML treatment managed transpiration rates effectively under high temperature and saline stresses, maintaining elevated levels of stomatal conductance and carbon dioxide uptake [53].

ROS_s_ play a crucial role as signaling molecules essential for regulating plant development and responses to diverse stimuli, notwithstanding their capacity to inflict harm on plants. This underscores the significance of varying reactivity levels, capacities, and ability to traverse biological membranes in shaping the dual nature of ROS. Oxidative stress arises in cells when ROS levels exceed the capacity of defense mechanisms to manage [54]. In our study, plants experiencing water scarcity showed higher oxidative stress, indicated by elevated levels of H_2_O_2_ and lipid peroxidation (MDA, and EL). This rise in oxidative stress triggers signaling pathways that disrupt the balance between ROS and the antioxidants that neutralize them, both enzymatic and non-enzymatic. Hence, plants rely on their inherent enzymatic and non-enzymatic antioxidant defenses to uphold the physiological function of ROS [54]. Based on our research, water deficit conditions led to an increase in total soluble protein levels and the activity of antioxidant enzymes. Interestingly, this rise was sustained even after being subjected to a combination of EBL and ML. BRs and ML have demonstrated noteworthy enhancements in the levels of soluble sugars and proteins in plants experiencing abiotic stresses [3, 23, 55].

As a potent antioxidant and redox balance regulator, many of ML's activities, especially its responses to stress tolerance, are derived from this role [15, 56]. Additionally, ML acts as a signaling molecule in plants, activating the mitogen-activated protein kinase (MAPK) pathway [15, 56] and subsequently inducing some genes associated with pathogen defense [57], protein quality maintenance [58], stomatal closure [59], and enhanced crop yield [60]. As a reflection of its diverse roles, ML's mode of action in plants is closely related to different plant hormones [61]. Arnao and Hernández Ruiz suggest that ML could act as a signaling molecule in abiotic stress conditions by upregulating anti-stress genes and increasing endogenous ML levels in response to stress [61].

In this study, the levels of oxidative damage, as indicated by H_2_O_2_, EL, and MDA, were elevated in chickpea plants subjected to water deficit conditions. Our study's results also indicate a decrease in oxidative damage following ML and EBL treatments under water deficiency conditions. Hence, by balancing ROS, treatments with ML and EBL can reduce oxidative damage and facilitate the repair of cellular membranes damaged by environmental stresses. The precise functions of ML and EBL in mitigating the detrimental impacts of abiotic stresses on various crops remain incompletely elucidated [25, 39]. ML and EBL regulate the levels of ROS/RNS and genes involved in the antioxidants production [62–64]. Pre-treatment of *Zea mays* seedlings with ML enhances levels of photosynthetic pigments, osmoprotectants accumulation, and RWC. This enhancement is linked to overall plant development and a reduction in ROS-induced oxidative damage [65]. The activation of antioxidant enzymes induced by ML might correlate with increased activity of mitochondrial ATP synthase and ATPase, as observed by Turk and Genisel, and Generozova et al. [66, 67]. This enhancement likely supported stressed plants in sustaining the requisite energy supply for normal enzyme function.

Our findings revealed that under water deficit conditions, the activities of SOD, POD, APX, GPX, and CAT were all heightened. However oxidative stress indicators like EL and MDA also significantly increased at the same time. This suggests that plants attempted to regulate ROS production under water deficit conditions by activating their antioxidant enzymes. However, these enzymes were not sufficiently effective in adequately scavenging ROS, resulting in damage to the stressed plants. Furthermore, ML and EBL enhanced the activity of antioxidant enzymes, leading to a significant decrease in H_2_O_2_ and MDA production, consistent with the findings reported by Imran et al. [68]. The reduction in ROS accumulation resulted in a notable decrease in ROS-induced damage, as evidenced by reductions in EL and MDA content in water-stressed plants treated with ML. The development of resilience to challenging environments through the exogenous application of ML appears to arise from both ML's strong antioxidative capabilities [69] and its critical function as a signaling molecule [70, 71]. In this study, observations revealed that EBL substantially boosted the activity of antioxidant enzymes in chickpea plants, suggesting its potential to regulate cellular redox changes by sustaining the activity of key antioxidant enzymes. The transitory apoplastic synthesis of H_2_O_2_ works as a molecular signal, triggering the BRs signaling cascade, and ultimately safeguarding the plant against the negative effects of abiotic stressors. BRs control the transcription of genes encoding proteins and enzymes required for stress resistance [39, 72].

We hypothesize that the enhanced resistance observed in chickpea plants treated with EBL may be attributed to oxidative stress-defense mechanisms. Numerous studies have demonstrated the potential of EBL application in improving tolerance to various abiotic stresses [73, 74] by maintaining a balance between ROS and antioxidants, as well as osmolyte accumulation. EBL, like melatonin, is produced in all plant cells, eliminating the need for long-distance transport; consequently, the de novo synthesis and turnover of EBL are pivotal in determining its levels. The EBL treatment significantly increases the contents of soluble carbohydrates and proteins, reducing osmotic stress caused by water deficiency [75, 76]. EBL modulates the expression of genes involved in sucrose, calcium ion, and the abscisic acid signaling pathways, antioxidant production, and membrane integrity, all crucial for enhancing plants' tolerance to abiotic stress [77]. Furthermore, it has been found that BRs interact with several other plant hormones to affect stress tolerance, plant growth, and development. For example, the simultaneous application of melatonin and BRs significantly increased *Festuca arundinacea*'s resilience to heat stress. This was demonstrated by an increase in chlorophyll content and antioxidant enzyme activities, as well as a decrease in MDA content and reactive ROS levels [78].

Our findings indicate a decrease in MDA and ROS levels, coupled with heightened activity of antioxidant enzymes, upon the application of exogenous EBL. This implies that EBL may be able to prevent membrane damage in chickpea plants exposed to water deficit conditions. Recent studies have proven that the application of exogenous EBL was associated with higher levels of hormones and antioxidant enzyme activities as well as transcript levels of defense-related genes [78]. Consequently, the enhancement of water deficit stress tolerance by EBL may be closely linked to its activation of the chickpea antioxidant defense system. This activation prevents the overproduction of ROS and MDA, thereby leading to an elevated membrane stability index [79]. Furthermore, chickpea plants treated with EBL and ML exhibited enhanced growth compared to untreated plants, highlighting the role of EBL and ML in plant responses to water deficit conditions.

Beyond their vital function of protecting plants from environmental challenges, BRs also play a pivotal role in sustainable crop production [22]. Hence, crop enhancement may be possible by modifying BR signaling, biosynthetic pathways, or sensory processes [80]. By activating CBFs (C-repeat binding factors), which regulate the expression of genes responding to stressful conditions, BRs reduce the excessive generation of ROS in plants under stress conditions. Furthermore, transcriptomic analysis has revealed that BRs regulate thousands of genes under stress conditions to enhance the plant defense system by improving the activities CAT and SOD [81]. Similarly, ML functions as a signaling molecule, following the pathway of BRs, with the ability to up-regulate several antioxidant enzymes [82] and act as a scavenger of superoxide (O^2−^) [83]. To summarize, ML controlled ROS levels through two mechanisms: firstly, by reducing ROS production by inhibiting the activity of ROS-producing enzymes, and secondly, by scavenging ROS by enhancing the antioxidant system's efficiency. This dual control over ROS allowed the stressed plants to resume their normal cellular functions [84]. In this study, the elevated levels of soluble sugars and proline induced by ML and EBL applications likely contributed to maintaining the improved hydration levels. These are attributed to their role in offering osmoprotection against abiotic stresses, as previously documented by [85, 86]. The sugars level and the expression of BR-related genes in numerous plants are interconnected [87]. The levels of BRs are associated with fluctuations in sugars concentration in Arabidopsis [88].

It has been shown in earlier studies that BRs increase the expression of genes involved in proline biosynthesis [89]. Furthermore, ML increases cell turgor and water-holding capacity to improve drought tolerance [48]. It also decreases water deficit in *Lupinus termis* by increasing nitrate reductase and antioxidant activity [90]. The application of exogenous ML was noted to elevate the endogenous levels of ML, sugars, and proline content, thereby enhancing seed germination rates under salt stress conditions. Elevated proline level in plants under water stress are commonly observed across numerous plant species [91]. Consistent with these findings, our study revealed a significant increase in proline accumulation under water deficit, regardless of the presence of EBL and/or ML.

Our comprehension of ML's unique functions in plant growth and development as well as defense mechanisms against stressors will be improved by more thorough research into the precise processes and interactions between EBR and ML. In this study, the application of EBL and ML together significantly altered proline and sugars accumulation, growth, photosynthetic traits, and the activities of various antioxidant enzymes in chickpea plants exposed to water deficit conditions. The increased levels of antioxidant enzymes, proline, and sugars accumulation indicate the mitigation of ROS induced by the concurrent application of EBL and ML, thereby providing tolerance against water deficit stress. This study underscores the effectiveness of co-applying EBL and ML as a viable strategy for alleviating water deficit stress in chickpea plants. It suggests the potential for utilizing this approach in sustainable agricultural practices, particularly in managing stress-prone soils. Furthermore, the study indicates that the simultaneous application of EBL and ML holds promising potential for enhancing crop yield under water deficit stress. Additionally, it may serve as a potent suppressor of oxidative stress in chickpea plants.

ML and EBL reduce ROS production in chickpea plants under water-deficit conditions through the utilization of various mechanisms. They upregulate antioxidant enzymes, which are crucial for scavenging ROS, and enhance levels of non-enzymatic antioxidants. They also influence the expression of stress-responsive genes, modulate stress signaling pathways, including abscisic acid signaling, improve water use efficiency, stabilize cell membranes by preventing lipid peroxidation, and enhance photosynthetic efficiency by protecting the photosynthetic apparatus and reducing photorespiratory loss. Through these combined actions, ML and EBL effectively reduce ROS production and enhance the overall stress tolerance of chickpea plants under water-deficit conditions [15, 81, 91, 92]. Despite notable advancements in recent years, there remain gaps in comprehending the impacts of exogenous ML and EBL on enhancing plants' resilience to abiotic stresses. Future research on the genetic and metabolic pathways involved in stress recovery, focusing on ML and EBL, should investigate specific molecular pathways, including key genes and proteins, and identify receptors and their signaling mechanisms. High-throughput RNA sequencing and genome-wide association studies (GWAS) can reveal differentially expressed genes and genetic loci associated with stress recovery mediated by these compounds. Proteomic and metabolomic analyses will help identify proteins and metabolic changes during treatment. Integrative omics combining genomics, transcriptomics, proteomics, and metabolomics, alongside comparative studies across species, will provide a comprehensive view. Functional genomics using CRISPR/Cas and RNAi to manipulate genes, along with overexpression [93] and knockout models, will elucidate the roles of specific genes [19, 92].

In our study, chickpea plants exposed to water deficit conditions exhibited increased oxidative stress, as indicated by higher levels of H_2_O_2_, MDA content, and EL. This was accompanied by the degradation of photosynthetic pigments, significant growth stunting, and excessive accumulation of ROS. The mechanisms through which ML and EBL mitigate oxidative stress primarily involve the interaction of various defensive response pathways [81, 91, 92, 94]. Applying EBR and ML significantly alleviates oxidative stress in chickpea plants by reducing ROS levels, maintaining photosynthetic pigments, and upregulating genes linked to enzymatic (CAT, SOD, POD, APX, and GPX) and non-enzymatic (flavonoids and carotenoids) antioxidant production. Furthermore, EBL and ML reduce MDA content and EL while notably increasing the levels of osmoprotectants, including proline, total soluble proteins, and sugars. It's been observed that BRs and ML control DNA methylation, a key factor in a plant's salt tolerance [15, 95]. For instance, seed priming with EBR led to DNA methylation and improved tolerance to salt [95]. Moreover, ML engages with epigenetic mechanisms, influencing gene expression via DNA methylation, histone modifications, and miRNA regulation. These modifications play a crucial role in shaping stress-responsive gene networks, which help plants respond to different kinds of stress. However, comprehending the intricate interplay through which ML orchestrates redox regulation and epigenetic signaling to develop innovative strategies for enhancing plant resilience against diverse abiotic stresses remains unknown [15].

### Comparative analysis of quality and nutritional characteristics affected by experimental treatments

**Fig. 4:**
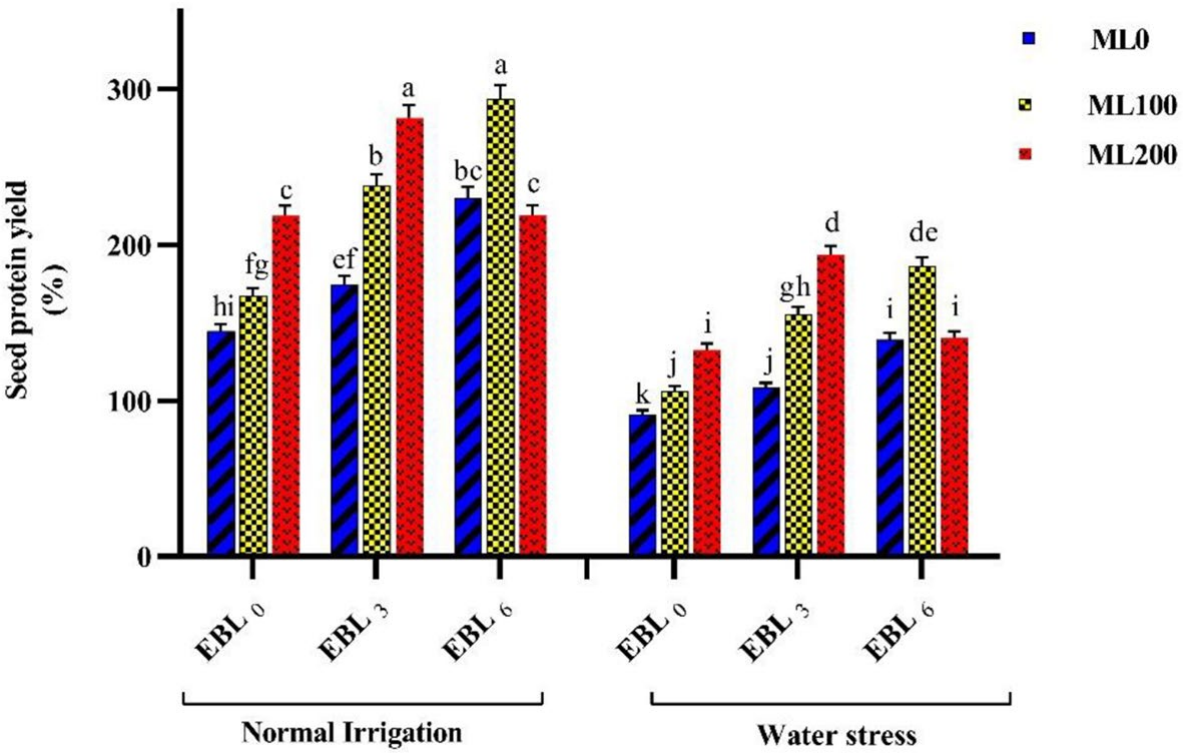
Illustrating the effects of different melatonin (ML) (0, 100, and 200 µM), and 24-epibrassinolide (EBL) (0, 3, and 6 µM) levels on the seed protein yield (SPY). Duncan's test was utilized to compare means at a 1% confidence level, indicating that columns sharing identical letters are not statistically distinct

The findings of this study verified that under water deficit conditions, the values of seed oil (%Oil), seed oil yield (SOY), and seed protein yield (SPY) traits decreased by 20%, 55%, and 36%, respectively. On the other hand, the levels of seed protein (%Pro) and seed nitrogen (%N) rose by 20% and 19%, respectively (Supplementary Table 3). Examining the interaction effects of different levels of ML*EBL on %Oil, SOY, SPY, %Pro, and %N revealed notable alterations in these traits influenced by the treatments. When EBL (6 µM) and ML (100 µM) were applied, the maximum values of %Oil (6.59), SOY (79.56 kg/ha), SPY (239.97 kg/ha), %Pro (20.89), and %N (3.34) were observed. Although no significant difference was observed for all these traits between plants treated with level 2 of EBL (3 µM) combined with level 3 of ML (200 µM) and plants treated with level 3 of EBL (6 µM) combined with level 2 of ML (100 µM). On the other hand, the lowest values observed for these attributes were 4.7%, 43 kg/ha, 117.8 kg/ha, 13.53%, and 2.16% for %Oil, SOY, SPY, %Pro, and 2.16%, respectively, without the application of EBL and ML. Certainly, applying level 3 of EBL and level 3 of ML on chickpea plants led to a 40%, 80%, 100%, 50%, and 57% rise in the values of %Oil, SOY, and SPY traits, respectively, compared to the absence of EBL and ML (Supplementary Table 11). Evaluation of the interaction effects of various EBL, and ML levels on the SPY revealed notable alterations in these traits influenced by the treatments. Under normal water conditions, the maximum value of SPY (293.67 kg/ha) was found when level 2 of ML (100 µM) and level 3 of EBL (6 µM) were applied simultaneously. Conversely, the lowest value of SPY was 91.12 kg/ha under water deficit conditions without these two elicitors' application. Indeed, treating chickpea plants with levels 3 of EBL (6 µM) and 2 of ML (100 µM) under normal water conditions resulted in a respective increase of 100% in this trait compared to whenthey were not utilized (Fig. 4).

Several strategies can be considered to increase the effectiveness of ML and EBL in enhancing plant resistance to abiotic stresses: optimizing dosage and application methods by fine-tuning the concentration and timing of ML and EBL application; investigating synergistic interactions by examining how ML and EBL interact with other stress-alleviating agents or hormones to create combinations that enhance plant resilience; utilizing advanced delivery systems such as nanoparticles or microencapsulation to improve targeted delivery and uptake of ML and EBL by plants; employing genetic engineering techniques to enhance endogenous production of ML or modulate the plant's responsiveness to EBL to amplify their protective effects; and conducting extensive field trials to validate the efficacy of ML and EBL under various environmental conditions and crop species. Together, these strategies seek to increase the effectiveness of ML and EBL in strengthening plant resistance to abiotic stressors, ultimately contributing to sustainable crop production [39, 61, 64, 71, 81].

### Principal component analysis

Principal component analysis (PCA) was used in this study to identify important physiological, biochemical, and growth variables associated with chickpea plant tolerance to water deficit conditions [96]. The significance of statistical instruments including PCA, analysis of variance, and correlation types is considered in evaluating the relationship between drought, growth regulators, and agronomic, physiological, and biochemical characteristics [97]. The outcomes of the PCA revealed that the first three components accounted for 96.98% of the overall variations. The first component contributed 63.4% to the total variance, encompassing crucial traits such as Chl a, Chl b, Tot chl, Cart, H_2_O_2_, RWC, PH, NMB, NLB, NSP, NPP, PWP, W100S, W100P, SSY, harvest index (HI), MBD, SC, PSP, GY, %Oil, SOY, and SPY. Given that most of the attributes associated with this component connected to yield and its components, it is fitting to label it as a "yield component" component. Concurrently, the second component, which accounted for 29.8% of the total variations, included antioxidant enzymes, So pro, MDA, EL, Prol, Flavo, Phe, and Sug. As almost all attributes linked to this component relate to enzymatic and non-enzymatic antioxidants, it can fittingly be termed a "defense components" component. The third component, which accounted for only 3.78% of the total variations, included %N and %Pro (Table 2).

**Table 2:** Eigenvalues, eigen vectors and cumulative variance of the investigated traits in this study

**Traits**	**Components**			**Traits**	**Components**		
	**1**	**2**	**3**		**1**	**2**	**3**
**Chl a**^**a**^	**.987**	.111	-.021	**NMB**	**.946**	.264	-.140
**Chl b**	**.986**	.054	-.031	**NLB**	**.893**	.425	-.023
**Tot chl**	**.988**	.092	-.025	**NSP**	**.990**	.107	-.032
**Cart**	**.985**	.141	-.077	**NPP**	**.992**	.099	-.064
**Cat**	-.479	**.867**	-.067	**PWP**	**.997**	.051	-.042
**SOD**	-.683	**.720**	-.089	**W100S**	**.978**	.172	-.062
**GPX**	-.475	**.873**	-.052	**W100P**	**.940**	.317	-.091
**APX**	-.539	**.833**	-.074	**SSY**	**.886**	.431	-.074
**PPO**	-.613	**.777**	-.098	**HI**	**.760**	-.526	.207
**So pro**	-.296	**.902**	-.043	**MBD**	**.930**	.344	-.053
**MDA**	-.549	**-.675**	.025	**SC**	**.987**	.145	-.054
**EL**	-.429	**-.720**	.039	**PSP**	**-.781**	-.605	.027
**H**_**2**_**O**_**2**_	**-.977**	-.166	.061	**GY**	**.986**	.121	-.049
**Prol**	-.534	**.834**	-.093	**%N**	-.375	.533	**.757**
**Flavo**	-.459	**.878**	-.125	**%Pro**	-.375	.533	**.757**
**Phe**	-.604	**.783**	-.125	**%Oil**	**.790**	.267	.543
**Sug**	-.365	**.913**	-.136	**SOY**	**.965**	.148	.164
**RWC**	**.997**	-.019	-.040	**SPY**	**.828**	.351	.405
**PH**	**.929**	.345	-.019	**-**	**-**	-	-
**Eigenvalues**	**23.65**	**10.39**	**1.13**	**Eigenvalues**	**23.65**	**10.39**	**1.13**
**Cumulative of variance (%)**	**63.4**	**93. 2**	**96.98**	**Cumulative of variance (%)**	**63.4**	**93. 2**	**96.98**

^a^Eigenvalues are significant ≥ 0.50

In their investigation of *Phaseolus vulgaris* L., [98] noted that the first and second components represented 38% and 20% of the total diversity, respectively, while the third component contributed 16%. The first component comprised traits such as pigment content, number of pods, length of pods, and weight of seeds. Conversely, the second component exhibited the highest coefficients for MDA, plant height, number of seeds, and proline content. Also in other research, the first six and the first two components explained 97.8%, and 57.16% of the total variance, respectively. The first component explained 37.45% of the diversity and included proline, sugars, phenol, total protein, flavonoids, antioxidant enzymes, and leaf length. On the other hand, characteristics including pigment content, MDA, number of pods, weight of pods, and seed yield were included in the second component, which accounted for 19.71% of the variation [1]. The provided examples illustrate the utility of PCA in identifying essential traits linked to plants' stress tolerance. These findings also illustrate the potential of PCA in guiding breeding programs aimed at developing stress-tolerant cultivars.

### PCA biplot analysis

**Fig. 5:**
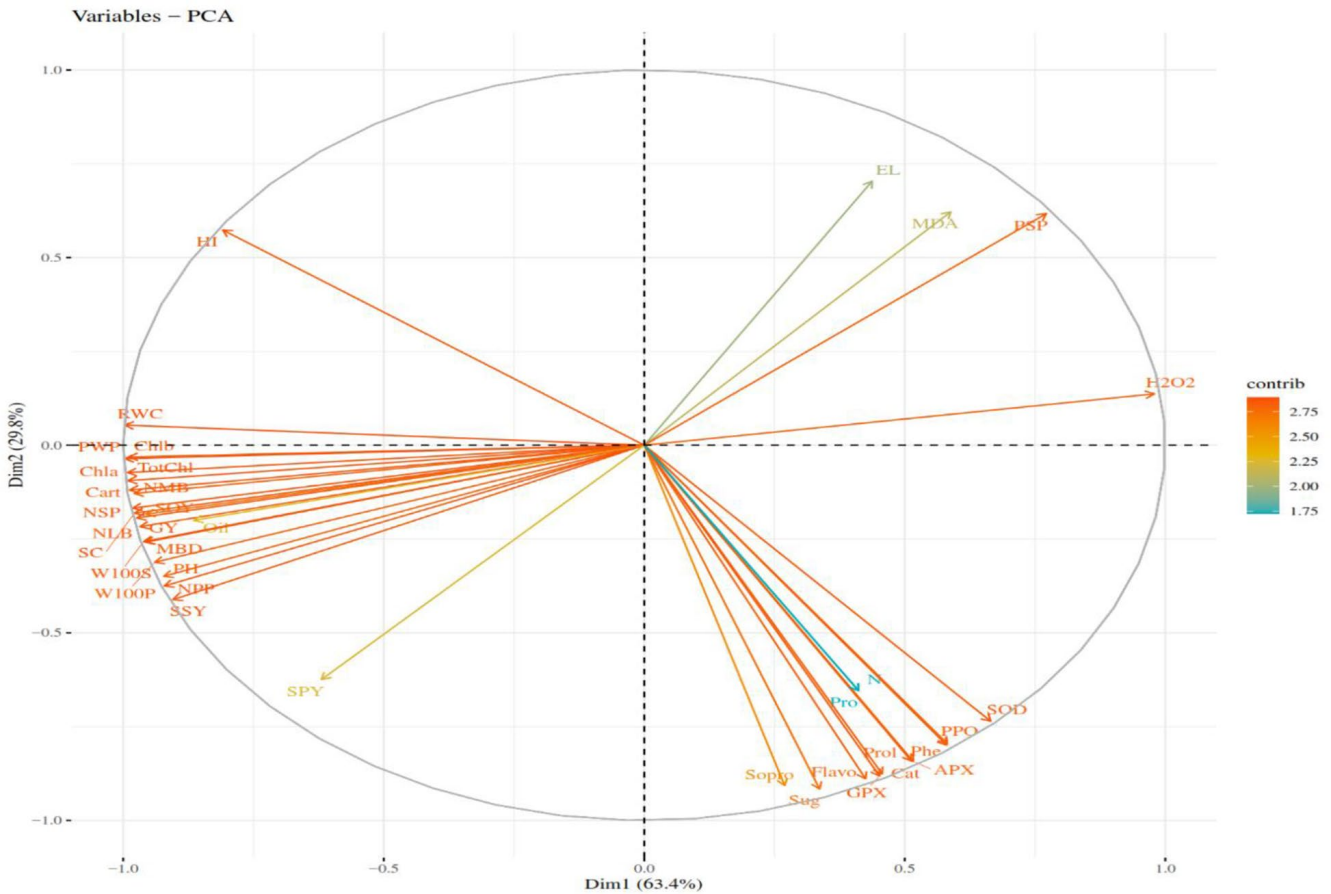
PCA biplots depicting relationships between the traits measured. PH: Plant height, NMB: Number of main branches, NLB: Number of lateral branches, NSP: Number of seeds per plant, NPP: Number of pods per plant, PWP: Pod weight per plant, W100S: 100-seed weight, W100P: 100-pod weight, SSY: Straw and stubble yield, HI: Harvest index, MBD: Main branch diameter, PSP: Percentage of seedless pods, GY: Grain Yield, SC: Stomatal Conductance, Chl a: Chlorophyll a, Chl b: Chlorophyll b, Tot Chl: Total chlorophyll, Cart: Carotenoid, MDA: Malondialdehyde, EL: Electrolyte leakage, CAT: Catalase, SOD: Superoxide dismutase, GPX: Guaiacol peroxidase, APX: Ascorbate peroxidase, PPO: Polyphenol oxidase, Tot pro: Total soluble protein, Prol: Proline, Flavo: Flavonoid, Phe: Phenol, Sug: Sugar, RWC: Relative water content, %N: Seed nitrogen, %Pro: Seed protein, %Oil: Seed oil, SOY: Seed oil yield, SPY: Seed protein yield

The PCA biplot emerges as a highly efficient multivariate analysis tool for evaluating genotypic yield and trait interactions. Extensive utilization by numerous researchers has been observed in evaluating trait correlations across various crop species using this method. Through PCA biplots, researchers have gained new insights into drought tolerance mechanisms and plant responses under drought-stress conditions [32, 99]. The PCA biplot reveals a noteworthy positive correlation between the Chl a, Chl b, To chl, Cart, H_2_O_2_, RWC, PH, NMB, NLB, NSP, NPP, PWP, W100S, W100P, SSY, HI, MBD, SC, PSP, %Oil, SOY, SPY, and GY traits. Notably, these traits accounted for a significant portion of the variation as the first component in the PCA analysis (Fig. 5). Furthermore, the PCA analysis revealed a strong positive association between the So pro, antioxidant enzymes, Prol, Flavo, Phe, Sug, %N, and %Pro features, which were included as the second and third components in the PCA analysis. Moreover, a negative and significant correlation was detected between MDA, EL, PSP, and H_2_O_2_ traits with GY (Fig. 5). These findings are consistent with those reported by Sachdeva et al., who, utilizing PCA biplot and correlation analyses, identified strong positive correlations between RWC, chlorophyll index, and secondary branches with seed yield. The results of this investigation are consistent with other studies providing a strong and positive correlation between proline concentration in the PCA biplot and antioxidant enzymes [100]. Furthermore, in keeping with the findings of our investigation, other researches [32, 101] demonstrated a negative and substantial connection between EL and MDA with antioxidant enzymes and proline concentration. Consistent with the current research's findings, Rani et al.'s investigation demonstrated a positive and substantial association between MDA, EA, and H_2_O_2_ in leaves, anthers, and ovules of chickpea plants under drought stress. These traits signify damage induced by water deficits in chickpea plants, displaying adverse associations with other traits like yield and biochemical characteristics [101]. The positive and strong correlations between GY and RWC, pigment content, and %Oil imply that these characteristics may serve as useful indicators for selecting genotypes with consistent yield in stressful environments. In addition to yield components, these positively associated physio-biochemical characteristics collectively contribute significantly to chickpea plants' ability to withstand water deficits. Hence, they can serve as valuable indicators for assessing the ability of chickpea varieties to withstand water deficits. PCA was employed by Lazic et al. to assess plant responses to experimental queries concerning rapeseed [102].

### Correlation analysis

Evaluating the correlation coefficients among different traits can refine decision-making regarding indirect selection indicators and the elimination of undesirable traits. Trait correlations may arise from pleiotropy or strong linkage between genes governing these traits [103, 104]. The correlation analysis's findings indicated a significant positive correlation between GY with Chl a (0.69**), Chl b (0.59**), Cart (0.69**), RWC (0.77***), PH (0.66**), NMB (0.78***), NLB (0.72***), NSP (0.799***), NPP (0.79***), PWP (0.69**), W100S (0.79***), W100P (0.77***), SSY (0.84***), HI (0.56**), MBD (0.76***), SC (0.79***), SPY (0.74***), and SOY (0.80***) traits. Furthermore, a negative correlation was documented between GY with traits MDA (-0.57*), EL (-0.49*), PSP (-0.72***), H_2_O_2_ (-0.77***)_,_ Phe (-0.49*), SOD (-0.50*), and PPO (-0.49*) (Table 3). Significant positive and negative correlations between seed yield and various traits, including plant height, protein content, RWC, sub-branches, and biomass in chickpea plants subjected to drought stress conditions, have been established in previous research [100, 105]. Moreover, previous studies have demonstrated a robust correlation between the number of seeds per plant and per pod with yield, making them viable indirect selection indices for genotypes with high yield. Under stress conditions, legumes display a range of morpho-physiological and biochemical changes, emphasizing the importance of assessing drought-related traits for chickpea breeders to identify drought-tolerant genotypes. Furthermore, plant selection with water absorption abilities from the soil is a vital strategy for fortifying genetic resilience to drought [106, 107]. The current investigation revealed a significant positive correlation between GY with traits such as NMB, NLB, PH, and NBD, indicating their potential as indirect selection criteria in future breeding programs for drought-stressed chickpea plants. Furthermore, increased NMB and NLB may enhance the leaf area index, possibly accelerating growth rates by elevating levels of photosynthetic pigments and enhancing photosynthesis efficiency [108, 109].

**Table 3:** The correlation coefficients among the examined traits in this investigation

	**Chl a**	**Chl b**	**Tot chl**	**Cart**	**Cat**	**SOD**	**GPX**	**APX**	**PPO**	**So pro**
**Chl a**	1									
**Chl b**	0.90^***^	1								
**Tot chl**	0.90^***^	0.93^***^	1							
**Cart**	0.90^***^	0.92^***^	0.91^***^	1						
**Cat**	-0.36^ ns^	-0.40^ ns^	-0.38^ ns^	-0.34^ ns^	1					
**SOD**	-0.59^**^	-0.54^**^	-0.58^**^	-0.56^*^	0.90^***^	1				
**GPX**	-0.36^ ns^	-0.40^ ns^	-0.37^ ns^	-0.33^ ns^	0.91^***^	0.90^***^	1			
**APX**	-0.42^ ns^	-0.47^*^	-0.44^ ns^	-0.40^ ns^	0.91^***^	0.91^***^	0.89^***^	1		
**PPO**	-0.50^*^	-0.54^*^	-0.52^*^	-0.48^*^	0.90^***^	0.91^***^	0.90^***^	0.89^***^	1	
**So pro**	-0.16^ ns^	-0.19^*^	-0.17^*^	-0.15^ ns^	0.90^***^	0.80^***^	0.91^***^	0.93^***^	0.91^***^	1
**MDA**	-0.57^*^	-0.51^*^	-0.55^*^	-0.63^**^	-0.27^ ns^	-0.14^ ns^	-0.29^ ns^	-0.22^ ns^	-0.14^ ns^	-0.31^ ns^
**EL**	-0.44^ ns^	-0.38^ ns^	-0.42^ ns^	-0.52^*^	-0.36^ ns^	-0.26^ ns^	-0.38^ ns^	-0.32^ ns^	-0.25^ ns^	-0.37^ ns^
**H**_**2**_**O**_**2**_	-0.89^***^	-0.85^***^	-0.86^***^	-0.88^***^	0.33^ ns^	0.53^*^	0.32^ ns^	0.39^ ns^	0.47^*^	0.16^ ns^
**Prol**	-0.42^ ns^	-0.46^*^	-0.44^ ns^	-0.40^ ns^	0.79^***^	0.77^***^	0.79^***^	0.79^***^	0.78^***^	0.83^***^
**Flavo**	-0.35^ ns^	-0.40^ ns^	-0.37^ ns^	-0.31^ ns^	0.68^**^	0.75^***^	0.78^***^	0.78^***^	0.77^***^	0.82^***^
**Phe**	-0.50^*^	-0.54^*^	-0.52^*^	-0.47^*^	0.67^**^	0.78^***^	0.77^***^	0.78^***^	0.79^***^	0.78^***^
**Sug**	-0.26^ ns^	-0.31^ ns^	-0.27^ ns^	-0.21^ ns^	0.67^**^	0.72^***^	0.77^***^	0.86^***^	0.84^***^	0.71^***^
**RWC**	0.87^***^	0.87^***^	0.89^***^	0.78^***^	-0.49^*^	-0.58^**^	-0.49^*^	-0.55^*^	-0.52^*^	-0.32^ ns^
**PH**	0.75^***^	0.74^***^	0.75^***^	0.76^***^	-0.13^ ns^	-0.39^ ns^	-0.13^ ns^	-0.20^ ns^	-0.29^ ns^	0.05^ ns^
**NMB**	0.77^***^	0.76^***^	0.76^***^	0.77^***^	-0.20^*^	-0.44^ ns^	-0.20^ ns^	-0.27^ ns^	-0.35^ ns^	-0.01^ ns^
**NLB**	0.72^***^	0.69^**^	0.71^***^	0.73^***^	-0.06^ ns^	-0.29^ ns^	-0.05^ ns^	-0.13^ ns^	-0.21^ ns^	0.10^ ns^
**NSP**	0.69^**^	0.58^**^	0.59^**^	0.69^**^	-0.37^ ns^	-0.50^*^	-0.37^ ns^	-0.43^ ns^	-0.51^*^	-0.19^ ns^
**NPP**	0.68^**^	0.58^**^	0.58^**^	0.69^**^	-0.38^ ns^	-0.51^*^	-0.38^ ns^	-0.44^ ns^	-0.52^*^	-0.20^ ns^
**PWP**	0.69^**^	0.59^**^	0.59^**^	0.69^**^	-0.42^ ns^	-0.60^**^	-0.42^ ns^	-0.48^*^	-0.56^*^	-0.23^ ns^
**W100S**	0.69^**^	0.68^**^	0.59^**^	0.69^**^	-0.30^ ns^	-0.50^*^	-0.30^ ns^	-0.37^ ns^	-0.45^ ns^	-0.11^ ns^
**W100P**	0.66^**^	0.55^**^	0.56^**^	0.67^**^	-0.16^ ns^	-0.40^ ns^	-0.15^ ns^	-0.23^ ns^	-0.31^ ns^	0.02^ ns^
**SSY**	0.73^***^	0.62^**^	0.73^***^	0.73^***^	-0.03^ ns^	-0.29^ ns^	-0.03^ ns^	-0.10^ ns^	-0.18^ ns^	0.17^ ns^
**HI**	0.57^*^	0.69^**^	0.58^**^	0.55^**^	-0.54^*^	-0.59^**^	-0.52^*^	-0.47^*^	-0.49^*^	-0.53^*^
**MBD**	0.66^**^	0.65^**^	0.66^**^	0.67^**^	-0.13^ ns^	-0.38^ ns^	-0.12^ ns^	-0.20^ ns^	-0.28^ ns^	0.06^ ns^
**SC**	0.69^**^	0.67^**^	0.68^**^	0.69^**^	-0.34^ ns^	-0.56^*^	-0.33^ ns^	-0.40^ ns^	-0.48^*^	-0.16^ ns^
**PSP**	-0.52^*^	-0.58^**^	-0.51^*^	-0.65^**^	-0.13^ ns^	0.08^ ns^	-0.14^ ns^	-0.07^ ns^	0.02^ ns^	-0.27^ ns^
**GY**	0.69^**^	0.59^**^	0.59^**^	0.69^**^	-0.35^ ns^	-0.50^*^	-0.35^ ns^	-0.41^ ns^	-0.49^*^	-0.15^ ns^
**%N**	-0.32^ ns^	-0.36^ ns^	-0.33^ ns^	-0.35^ ns^	0.49^*^	0.50^*^	0.49^*^	0.50^*^	0.57^*^	0.56^*^
**%Pro**	-0.32^ ns^	-0.36^ ns^	-0.33^ ns^	-0.35^ ns^	0.49^*^	0.51^*^	0.50^*^	0.59^**^	0.57^*^	0.56^*^
**%Oil**	0.59^**^	0.57^**^	0.58^**^	0.67^**^	-0.18^ ns^	-0.39^ ns^	-0.17^ ns^	-0.24^ ns^	-0.33^ ns^	-0.03^ ns^
**SOY**	0.57^**^	0.57^**^	0.57^**^	0.65^**^	-0.33^ ns^	-0.57^*^	-0.33^ ns^	-0.39^ ns^	-0.48^*^	-0.13^ ns^
**SPY**	0.66^**^	0.62^**^	0.65^**^	0.63^**^	-0.10^ ns^	-0.35^ ns^	-0.10^ ns^	-0.17^ ns^	-0.26^ ns^	0.08^ ns^
	**MDA**	**EL**	**H**_**2**_**O**_**2**_	**Prol**	**Flavo**	**Phe**	**Sug**	**RWC**	**PH**	**NMB**
**MDA**	1									
**EL**	0.88^***^	1								
**H**_**2**_**O**_**2**_	0.70^**^	0.59^**^	1							
**Prol**	-0.24^ ns^	-0.34^ ns^	0.38^ ns^	1						
**Flavo**	-0.34^ ns^	-0.44^ ns^	0.29^ ns^	0.78^***^	1					
**Phe**	-0.19^ ns^	-0.30^ ns^	0.45^ ns^	0.78^***^	0.88^***^	1				
**Sug**	-0.43^ ns^	-0.52^*^	0.18^*^	0.66^**^	0.88^***^	0.85^***^	1			
**RWC**	-0.55^*^	-0.43^ ns^	-0.77^***^	-0.54^*^	-0.46^*^	-0.51^**^	-0.37^ ns^	1		
**PH**	-0.71^***^	-0.61^**^	-0.75^***^	-0.20^ ns^	-0.12^ ns^	-0.29^ ns^	-0.02^ ns^	0.71^**^	1	
**NMB**	-0.65^**^	-0.55^*^	-0.76^***^	-0.26^ ns^	-0.18^ ns^	-0.34^ ns^	-0.08^ ns^	0.64^**^	0.67^**^	1
**NLB**	-0.70^**^	-0.61^**^	-0.64^**^	-0.12^ ns^	-0.03^ ns^	-0.20^ ns^	0.05^ ns^	0.68^**^	0.57^**^	0.75^***^
**NSP**	-0.60^**^	-0.49^*^	-0.88^***^	-0.43^ ns^	-0.35^ ns^	-0.50^*^	-0.25^ ns^	0.68^**^	0.56^**^	0.67^**^
**NPP**	-0.61^**^	-0.50^*^	-0.89^***^	-0.44^ ns^	-0.36^ ns^	-0.51^*^	-0.25^ ns^	0.79^***^	0.55^**^	0.67^**^
**PWP**	-0.56^*^	-0.44^ ns^	-0.88^***^	-0.48^*^	-0.40^ ns^	-0.45^*^	-0.31^ ns^	0.79^***^	0.54^**^	0.66^**^
**W100S**	-0.62^**^	-0.51^*^	-0.77^***^	-0.36^ ns^	-0.29^ ns^	-0.44^ ns^	-0.19^ ns^	0.57^**^	0.67^**^	0.68^**^
**W100P**	-0.70^**^	-0.6^**^	-0.696^**^	-0.22^ ns^	-0.14^ ns^	-0.30^ ns^	-0.04^ ns^	0.53^**^	0.69^**^	0.69^**^
**SSY**	-0.71^***^	-0.61^**^	-0.72^***^	-0.10^ ns^	-0.01^ ns^	-0.18^ ns^	0.06^ ns^	0.67^**^	0.68^**^	0.57^**^
**HI**	-0.09^ ns^	0.008^ ns^	-0.55^**^	-0.66^**^	-0.73^***^	-0.68^**^	-0.56^**^	0.56^**^	0.49^*^	0.56^*^
**MBD**	-0.69^**^	-0.59^**^	-0.75^***^	-0.19^ ns^	-0.11^ ns^	-0.28^ ns^	-0.02^ ns^	0.71^***^	0.68^**^	0.58^**^
**SC**	-0.64^**^	-0.53^*^	-0.79^***^	-0.39^ ns^	-0.31^ ns^	-0.47^*^	-0.21^ ns^	0.78^***^	0.66^**^	0.77^***^
**PSP**	0.80^***^	0.74^***^	0.68^**^	-0.08^ ns^	-0.17^ ns^	-0.004^ ns^	-0.27^ ns^	-0.57^**^	-0.52^*^	-0.69^***^
**GY**	-0.57^*^	-0.49^*^	-0.77^***^	-0.41^ ns^	-0.34^ ns^	-0.49^*^	-0.24^ ns^	0.77^***^	0.66^**^	0.78^***^
**%N**	-0.13^ ns^	-0.18^ ns^	0.32^ ns^	0.57^*^	0.54^*^	0.54^*^	0.51^*^	-0.41^ ns^	-0.17^ ns^	-0.31^ ns^
**%Pro**	-0.13^ ns^	-0.18^ ns^	0.32^ ns^	0.57^*^	0.54^*^	0.54^*^	0.51^*^	-0.41^ ns^	-0.17^ ns^	-0.31^ ns^
**%Oil**	-0.62^**^	-0.53^*^	-0.58^**^	-0.25^ ns^	-0.19^ ns^	-0.33^ ns^	-0.11^ ns^	0.56^**^	0.51^*^	0.73^***^
**SOY**	-0.57^*^	-0.45^ ns^	-0.74^***^	-0.40^ ns^	-0.33^ ns^	-0.48^*^	-0.24^ ns^	0.64^**^	0.64^**^	0.63^**^
**SPY**	-0.62^**^	-0.52^*^	-0.63^**^	-0.17^ ns^	-0.12^ ns^	-0.27^ ns^	-0.04^ ns^	0.69^**^	0.68^**^	0.72^***^
	**NLB**	**NSP**	**NPP**	**PWP**	**W100S**	**W100P**	**SSY**	**HI**	**MBD**	**SC**
**NLB**	1									
**NSP**	0.72^***^	1								
**NPP**	0.72^***^	0.79^***^	1							
**PWP**	0.71^***^	0.79^***^	0.79^***^	1						
**W100S**	0.74^***^	0.79^***^	0.78^***^	0.78^***^	1					
**W100P**	0.77^***^	0.77^***^	0.76^***^	0.85^***^	0.88^***^	1				
**SSY**	0.77^***^	0.72^***^	0.72^***^	0.71^***^	0.75^***^	0.78^***^	1			
**HI**	0.45^ ns^	0.67^**^	0.69^**^	0.62^**^	0.54^*^	0.52^*^	0.41^ ns^	1		
**MBD**	0.77^***^	0.76^***^	0.75^***^	0.85^***^	0.77^***^	0.79^***^	0.88^***^	0.51^*^	1	
**SC**	0.54^*^	0.59^**^	0.59^**^	0.69^**^	0.59^**^	0.67^**^	0.63^**^	0.57^**^	0.57^**^	1
**PSP**	-0.86^***^	-0.73^***^	-0.63^**^	-0.60^**^	-0.66^**^	-0.72^***^	-0.73^***^	-0.28^ ns^	-0.72^***^	-0.66^**^
**GY**	0.72^***^	0.79^***^	0.79^***^	0.69^**^	0.79^***^	0.77^***^	0.84^***^	0.56^*^	0.76^***^	0.79^***^
**%N**	-0.12^ ns^	-0.33^ ns^	-0.36^ ns^	-0.37^ ns^	-0.32^ ns^	-0.24^ ns^	-0.15^ ns^	-0.40^ ns^	-0.20^ ns^	-0.33^ ns^
**%Pro**	-0.12^ ns^	-0.33^ ns^	-0.36^ ns^	-0.37^ ns^	-0.32^ ns^	-0.24^ ns^	-0.15^ ns^	-0.40^ ns^	-0.20^ ns^	-0.33^ ns^
**%Oil**	0.70^**^	0.69^**^	0.67^**^	0.67^**^	0.68^**^	0.67^**^	0.66^**^	0.56^*^	0.69^**^	0.59^**^
**SOY**	0.71^**^	0.77^***^	0.75^***^	0.76^***^	0.76^***^	0.74^***^	0.72^***^	0.66^**^	0.74^***^	0.76^***^
**SPY**	0.76^***^	0.75^***^	0.62^**^	0.72^***^	0.75^***^	0.65^**^	0.86^***^	0.49^*^	0.67^**^	0.64^**^
	**PSP**		**GY**		**%N**		**%Pro**	**%Oil**	**SOY**	**SPY**
**PSP**	1									
**GY**	-0.72^***^		1							
**%N**	-0.007^ ns^		-0.34^ ns^		1					
**%Pro**	-0.007^ ns^		-0.34^ ns^		0.95^***^		1			
**%Oil**	-0.67^**^		0.60^**^		0.25^ ns^		0.25^ ns^	1		
**SOY**	-0.72^***^		0.80^***^		-0.16^ ns^		-0.16^ ns^	0.89^***^	1	
**SPY**	-0.73^***^		0.74^***^		0.18^ ns^		0.18^ ns^	0.76^***^	0.70^**^	1

*PH* Plant height, *NMB* Number of main branches, *NLB* Number of lateral branches, *NSP* Number of seeds per plant, *NPP* Number of pods per plant, *PWP* Pod weight per plant, *W100S* 100-seed weight, *W100P* 100-pod weight, *SSY* Straw and stubble yield, *HI* Harvest index, *MBD* Main branch diameter, *PSP* Percentage of seedless pods, *GY* Grain Yield, *SC* Stomatal Conductance, *Chl a* Chlorophyll a, *Chl b* Chlorophyll b, *Tot chl* Total chlorophyll, *Cart* Carotenoid, *MDA* Malondialdehyde, *EL* Electrolyte leakage, *CAT* Catalase, *SOD* Superoxide dismutase, *GPX* Guaiacol peroxidase, *APX* Ascorbate peroxidase, *PPO* Polyphenol oxidase, *So pro* Total soluble protein, *Prol* Proline, *Flavo* Flavonoid, *Phe* Phenol, *Sug* Sugar, *H*_*2*_*O*_*2*_ Hydrogen peroxide, *RWC* Relative water content, *%N* Seed nitrogen, *%Pro* Seed protein, *%Oil* Seed oil, *SOY* Seed oil yield, *SPY* Seed protein yield

^*^Correlation is significant at the 0.05 level

^**^Correlation is significant at the 0.01 level

***Correlation is significant at the 0.001 level

A robust and statistically significant association was found in this study between GY and yield components. This outcome suggested that treating plants with EBL and ML led to heightened dry matter accumulation and yield components, ultimately leading to enhanced yield. Enhanced length and breadth of leaves, indicative of better leaf development, contribute to the positive effects on plant growth and production. Moreover, the optimization of pigment content likely contributed to the enhancement of photosynthetic efficiency and biomass production. These results highlight the potential of EBL and ML as useful components to support crop productivity in farming systems [1, 46]. Given the robust and significant correlation between GY and traits assessed in this study, selection based on these traits holds promise for enhancing GY under water stress conditions. The direct and indirect effects of desired traits on seed yield must be considered in breeding programs.

Breeders can determine which traits to prioritize in their breeding programs by carefully examining the direct and indirect effects of various characteristics associated with seed yield. This approach ensures that breeding programs concentrate on developing varieties harboring multiple desirable traits that collectively augment seed yield potential [110, 111]. In the current study, notable positive or negative correlations were observed between GY and yield components with %Oil and %Pro, SOY, and SPY. Sellami et al.'s findings indicated that, apart from protein yield, which showed a significant correlation with seed yield (0.96**), there were no notable correlations between yield components and qualitative features. The results are inconsistent with our current investigation, as we observed a significant correlation between yield and several yield components with chickpea quality traits under water deficit conditions [112]. This correlation could be pivotal in breeding programs focused on selecting and cultivating genotypes with superior quality attributes. Developing drought-tolerant chickpea varieties has posed a significant challenge for breeders [113]. This challenge arises from the scarcity of reliable selection indices, which predominantly rely on morphological and physiological responses and can be efficiently utilized [114]. Discovering new genetic resources capable of thriving in drought-stressed conditions is paramount. Nonetheless, relying solely on early-stage growth traits might not suffice to attain high yields or drought tolerance throughout the plant life cycle [32, 115].

### Cluster analysis

**Fig. 6:**
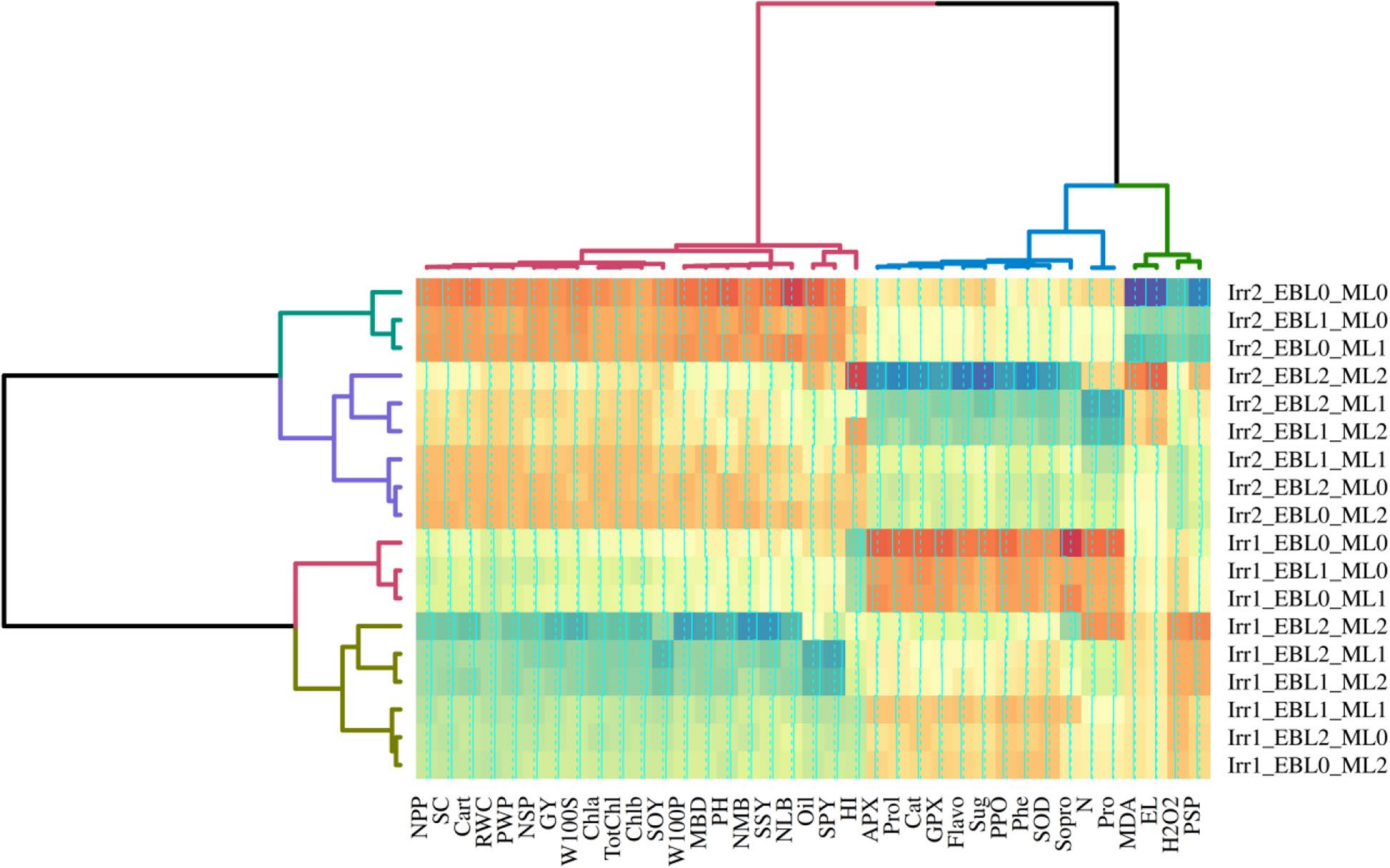
Heatmap generated from cluster analysis of treatments and examined traits employing the ward method. Irr: Different levels of irrigation (Irr1: normal irrigation; Irr2: water deficit conditions), EBL: Different concetrations of 24-epibrassinolide (0, 3, and 6 µM), ML: Different concetrations of melatonin (0, 100, and 200 µM)

The cluster analysis conducted on the treatments and traits examined in this study yielded significant insights. A two-dimensional heatmap was generated to illustrate the categorization of traits into three main groups and treatments into two main groups. One notable finding was that all normal irrigation treatments (8 days) were grouped together, whereas water deficit conditions treatments formed separate clusters. The heatmap analysis revealed that group 2 comprised physiological, biochemical, and qualitative traits such as Sug, Flavo, Prol, Phe, So Pro, antioxidant enzymes, %N, and %Pro. In contrast, group 1 included a combination of yield, yield components, and certain qualitative traits related to photosynthesis. Furthermore, the traits associated with the vulnerability of the cell membrane (EL, MDA, and H_2_O_2_) of chickpea plants (Fig. 6) and PSP were included in group 3. The findings in this section displayed full alignment with the outcomes of the correlation analysis, PCA, and PCA biplot, as these analyses affirmed the strong and direct relationships among these three categories of traits. Furthermore, the experimental treatments were distinctly categorized into two groups based on irrigation treatments: normal irrigation and water deficit conditions.

One intriguing pattern was found in each irrigation treatment concerning the combined effects of ML and EBL. For example, in the normal irrigation group, treatments that involved the combined effects of different levels of the two elicitors were divided into three groups. The first group consisted of scenarios in which neither elicitor was used, plus the application of level 2 of EBL and level 1 of ML, or vice versa. The second group included level 3 of both elicitors, level 2 of EBL, and level 3 of ML, or vice versa. In the meanwhile, level 2 of both elicitors and level 1 of EBL with level 3 of ML, or vice versa, made up the third group (Fig. 6). Dadasoglu et al. separated the experimental treatments using a heatmap analysis, considering the combined impacts of various salinity levels and melatonin. The study effectively categorized salinity treatments into three distinct groups (0, 75, and 150 mM) and melatonin treatments (0, 50, and 100 µM) into completely separable groups [116].

## Conclusion

This study investigated the decline in chickpea plants resilience to water deficit stress, characterized by reduced photosynthesis, inhibited growth, and increased ROS accumulation. However, the application of ML and EBL effectively mitigated these effects by enhancing antioxidant defenses and reducing ROS production. Additionally, ML and EBL treatments enhanced the growth of chickpea plants, bolstered photosynthetic efficiency, and facilitated the recovery of growth traits. Further research is necessary to uncover the genetic and metabolic pathways involved in stress recovery, aided by ML and EBL. The authors propose several techniques for future investigations into plant responses to ML and EBL. These include transcriptomic analysis using high-throughput RNA sequencing to scrutinize gene expression alterations, proteomic profiling employing mass spectrometry to pinpoint signaling proteins, and metabolomic studies to assess changes in metabolite profiles. We suggest utilizing functional genomics techniques like ChIP-Seq and ATAC-Seq to examine chromatin structure and transcription factor binding, genome editing technologies like CRISPR/CAS to generate mutant plant lines, and combining omics data with bioinformatics tools for thorough analysis. Together, these techniques provide a comprehensive understanding of plant responses to ML and EBL at the molecular level.

## Methods

### Experimental design and treatment application

The experiments were conducted during the March seasons of 2022 and 2023 at the Department of Agriculture and Plant Breeding, Faculty of Agriculture, Shahrood University of Technology, Iran. The plant material was obtained under the supervision and permission of the Ministry of Agriculture Jihad of Tehran, Iran, as well as national guidelines, with all authors complying with all local and national guidelines. Before commencing chickpea planting, the analysis of the farm soil was conducted, revealing a sandy loam texture and low organic matter content. The findings of the soil analysis conducted in the chickpea cultivation area are presented in Table 4.

**Table 4:** The analysis of physicochemical characteristics of farm soil

**Measured parameters**	Sand%	Silt%	Clay%	Organic carbon %	N %	K Ppm	P ppm	Zn ppm	Electrical conductivity (dS/m)
**Measured values**	20.1	49.2	30.7	0.4	0.1	280	10	1.1	1.5

The study employed a randomized complete block design with a factorial split-plot arrangement, replicated three times. The main plots included two irrigation periods (every 8 days and water deficit conditions), while the sub-plots featured varying concentrations of EBL (0, 3, and 6 µM) and ML (0, 100, and 200 µM). These concentrations were chosen based on preliminary experiments. The research was carried out in a cold, arid region with an annual average rainfall of 150 mm and an altitude of 1,360 m above sea level. Each plot comprised five rows, each measuring six meters in length. The rows were spaced 35 cm apart, with the plants within each row spaced 15 cm apart. Throughout plant growth and development, standard agricultural practices were also implemented to control weeds, pests, and diseases. A leaky irrigation system was installed in the field, and a water meter was placed at the beginning of the water inlet pathway to precisely track and quantify water consumed. Before spraying, a 0.1% surfactant was added to the stock solution. The Sigma-Merck Company provided the EBL (CAS number: 78821–43-9) and ML (CAS number: 78821–43-9) utilized in this study. At the flowering stage, EBL and ML were sprayed. The irrigation treatments included regular irrigation every eight days and water deficit stress, induced by halting irrigation before the onset of flowering. The physiological and morphological traits were assessed during the stages of post-flowering and complete seed ripening, respectively.

### The evaluation of morphological, yield and its component traits

Thirty plants were chosen from the middle three rows, leaving out the front and last 50 cm of each row. Numerous attributes such as PH, NMB, NLB, NSP, NPP, GY, PWP, W100S, W100P, SSY, HI, NBD, and PSP were evaluated. A very accurate electronic balance with a sensitivity of 0.001 g and a digital caliper were utilized to guarantee an exact evaluation of morphological features and yield components.

### The assessment of physiological and biochemical traits

#### Measurement of stomatal conductance

The SC measurements were conducted on the leaves utilizing an SC-1 leaf porometer (Decagon Devices, Pullman, WA, USA). The porometer was calibrated as per the instruction manual, and readings were obtained from both the adaxial and abaxial sides of a minimum of 20 distinct leaves at each specific time point [117].

#### Relative water content

The RWC was determined by selecting 80 fully developed leaves from the upper portion of each treated plant according to the method introduced by Kirnak et al. [118].

#### Pigment content

To quantify the levels of pigment, 0.25 g of leaves were homogenized in 5 ml of 85% acetone. The resulting mixture underwent centrifugation at 4000 rpm and 4 °C for 20 min. The supernatant's volume was then adjusted to 10 ml using 85% acetone, and the absorbance of the supernatant was measured at wavelengths of 480, 649, and 665 nm (UV-1800; Shimadzu Corporation, Kyoto, Japan) [119].

#### Measurement of malondialdehyde content

The MDA was determined using the Stewart and Bewley [120] method, involving spectrophotometric measurement of the samples' absorbance at 532 nm and 600 nm.

#### Measurement of electrolyte leakage index

Leaf discs (50 leaves), each measuring 0.15 by 0.15 cm, were put on a rotary shaker for 30 min after being immersed in distilled water. The EC meter (Weilheim, Germany) was used to measure the electrical conductivity (Ec1). The second electrical conductivity (Ec2) was then measured by shaking the leaf samples for 30 min in a hot water bath [121]. The membrane damage index resulting from cold and heat stress was computed using the formula: %EL = Ec1/Ec2 × 100.

#### Total soluble protein content and antioxidant enzymes assay

After applying the desired treatments, fully developed leaves located in the middle half of the stem were harvested using autoclaved scissors, immediately cooled using liquid nitrogen to stop all reactions, and transferred to a freezer at minus 80 °C.

#### Total soluble protein content assay

The total soluble protein content was assessed using the Bradford method, employing bovine serum albumin as a standard and a calibration curve [122].

#### Superoxide dismutase activity

The SOD activity was determined according to the procedure outlined by Acar et al., which measures the inhibition of the photochemical reduction of nitroblue tetrazolium (NBT) spectrophotometrically at 560 nm [123].

#### Catalase activity

The activity of CAT was determined using Aebi's method, which involves using H_2_O_2_ as the substrate and measuring the change in absorbance at 240 nm over 5 min [124].

#### Guaiacol peroxidase activity

The GPX activity was measured according to de Azevedo Neto et al. [125], using guaiacol as a substrate and determining the absorbance of the formed tetraguaiacol at 470 nm over 5 min [125].

#### Ascorbate peroxidase activity

The APX activity was assessed based on the method described by Madhusudhan et al. [126], utilizing H_2_O_2_ as the substrate and ascorbic acid as the reductant. The decrease in absorbance at 290 nm was monitored due to the oxidation of ascorbic acid [126].

#### Polyphenol oxidase activity

The PPO activity was assessed following the method outlined by Ogel et al., using pyrocatechol as the substrate, and measuring the O-quinone product at 420 nm [127].

#### Proline content assay

Bate's procedure [128] was used to determine proline concentration by measuring the absorbance of the organic phase at 520 nm and comparing it to a proline standard curve.

#### Measurement of total phenol content

Total phenol content was determined following the method of McDonald et al. [129]. The concentration was calculated by comparing absorbance values against a gallic acid standard curve of varying concentrations. The sample's absorbance was measured at 765 nm using a UV-1800 spectrophotometer.

#### Total soluble sugars

Schlegel's procedure [130] was employed to determine the concentration of total soluble sugars, with absorbance measurements of the samples conducted at a wavelength of 485 nm using a spectrophotometer.

#### Total flavonoid content assay

The flavonoid content was evaluated following the method outlined by Quettier-Deleu et al. [131], with absorbance measurements of the reaction mixture taken at 415 nm using a spectrophotometer..

#### Hydrogen peroxidase content assay

Initially, 5 mL of 1% trichloroacetic acid and 0.2 g of charcoal were combined with 0.5 g of powdered leaf tissue. Following centrifugation at 4000 rpm for 15 min, the mixture underwent filtration. Then, the prepared solutions (1 mL each) were blended with 1 mL of colorimetric reagent. Subsequently, 8 mg of catalase was introduced into these solutions, which were then incubated at 25 °C for 10 min. Finally, absorbance was recorded at 390 nm using a spectrophotometer [132].

### Evaluation of nutritional properties

#### Nitrogen and protein percentage

A mixture of acids consisting of 2.7 g of salicylic acid and 100 mL of sulfuric acid containing 3.5 g of selenium was added to 3 g of powdered seeds to start the digestion process. A digester oven (Gerhardt Company, Germany) and a fully automatic Kjeltec Analysis Vapodest 45S (Gerhardt Company, Germany) were used for distillation and titration, respectively. Subsequent steps were carried out following the Kjeldahl method [133]. Lastly, nitrogen and protein percentages were computed using the following formulas:Seed nitrogen% = (A × 6.4), where A = volume of 0.1 normal hydrochloric acid consumed in mL.

Seed protein % = 6.25 × nitrogen percentage [134].

#### Oil percentage determination

To extract oil, 5 g of powdered seeds were combined with 100 mL of N-hexane, occupying less than half the volume of the soxhlet flask within the thimble section of the soxhlet apparatus from Gerhardt Company, Germany. Subsequent procedures were carried out following Jensen's method [135]. To ensure the production of pristine oil, a vacuum evaporation apparatus was used to remove the solvent at reduced temperatures under AOCS Official Method-93 guidelines. Based on each sample's dry weight ratio, the oil content percentage (oil%) was calculated [136].

#### Statistical analyses

The data collected over two years of experimental work were analyzed using a randomized complete block design (RCBD). Significance was determined through analysis of variance (ANOVA) and Duncan multiple range test (DMRT) at *p* < 0.01 using SPSS V20 software (IBM, Armonk, NY, USA). The results are presented as mean values ± standard deviation (SD) based on triplicate observations. PCA and PCA-based biplots were generated using the FactoMineR package [137] within R studio software [137]. Pearson correlation analysis was conducted using the Corrplot package [138] in R studio software. Heatmap analysis was performed using agglomerative hierarchical clustering with STAR V2.0.1.

### Supplementary Information

Supplementary Material 1.

## Data Availability

The data generated or analyzed in this study are included in this article. Other materials that support the findings of this study are available from the corresponding author on reasonable request.
