# Supplementary material for: Synergistic effects of melatonin and 24-epibrassinolide on chickpea water deficit tolerance
Source: BMC Plant Biol. 2024 Jul 15;24:671. doi: 10.1186/s12870-024-05380-2 (PMC11247889; doi:10.1186/s12870-024-05380-2)
Supplement: Supplementary file 1 — Supplementary Material 1. [file 12870_2024_5380_MOESM1_ESM.docx]

**Table S1**. Variance analysis of evaluated traits of chickpea plants under different levels of water deficit, 24-epibrassinolide, and melatonin in two different year crops.

| **SOV** | **df** | **PH** | **NMB** | **NLB** | **NSP** | **NPP** | **PWP** | **W100S** | **W100P** | **SSY** | **HI** |
| --- | --- | --- | --- | --- | --- | --- | --- | --- | --- | --- | --- |
| **Year (Y)** | 1 | 654.65^***^ | 2.16^***^ | 611.09^***^ | 5900.32^***^ | 4041.69^***^ | 930.91^***^ | 618.84^***^ | 1454.98^***^ | 48853.78^***^ | 0.56^***^ |
| **Y*Rep** | 4 | 1.02^ns^ | 0.004^ns^ | 0.41^ns^ | 4.16^ns^ | 2.84^ns^ | 1.128^ns^ | 0.43^ns^ | 1.40^ns^ | 13.14^ns^ | 0.0001^ns^ |
| **Irr** | 1 | 456.74^***^ | 10.48^***^ | 154.80^***^ | 12447.44^***^ | 6466.86^***^ | 2254.63^***^ | 1373.75^***^ | 843.88^***^ | 21420.75^***^ | 0.04^***^ |
| **Y* Irr** | 1 | 28.11^**^ | 0.22^**^ | 1.22^ns^ | 686.29^***^ | 1894.08^***^ | 8.85^ns^ | 81.64^***^ | 0.11^ns^ | 907.12^*^ | 0.001^*^ |
| **Y* Irr *Rep** | 4 | 0.32^ns^ | 0.003^ns^ | 0.19^ns^ | 4.71^ns^ | 6.24^*^ | 1.81^ns^ | 0.76^ns^ | 0.43^ns^ | 54.40^ns^ | 0.0001^ns^ |
| **EBL** | 2 | 57.92^***^ | 1.24^***^ | 34.13^***^ | 581.70^***^ | 249.72^***^ | 67.95^***^ | 88.11^***^ | 102.04^***^ | 4859.70^***^ | 0.0009^**^ |
| **ML** | 2 | 64.82^***^ | 0.75^***^ | 22.89^***^ | 401.60^***^ | 214.84^***^ | 56.60^***^ | 70.12^***^ | 93.81^***^ | 3804.84^***^ | 0.002^***^ |
| **Y*EBL** | 2 | 1.49^ns^ | 0.07^**^ | 3.27^***^ | 5.32^ns^ | 0.39^ns^ | 0.65^ns^ | 0.06^ns^ | 0.31^ns^ | 185.14^**^ | 0.0002^ns^ |
| **Y*ML** | 2 | 3.36^*^ | 0.0009^ns^ | 0.97^ns^ | 1.74^ns^ | 0.22^ns^ | 0.06^ns^ | 0.11^ns^ | 0.84^ns^ | 142.06^**^ | 0.0005^*^ |
| **Irr * EBL** | 2 | 2.02^ns^ | 0.01^ns^ | 2.01^**^ | 7.15^ns^ | 5.01^ns^ | 0.54^ns^ | 0.33^ns^ | 0.61^ns^ | 75.11^ns^ | 0.0002^ns^ |
| **Irr *ML** | 2 | 0.50^ns^ | 0.01^ns^ | 0.004^ns^ | 5.59^ns^ | 3.07^ns^ | 0.24^ns^ | 0.84^ns^ | 0.16^ns^ | 104.86^*^ | 0.0005^*^ |
| **EBL *ML** | 4 | 0.36^ns^ | 0.06^***^ | 0.10^ns^ | 19.64^**^ | 6.30^*^ | 0.73^ns^ | 2.53^**^ | 2.95^*^ | 27.91^ns^ | 0.0005^**^ |
| **Y* Irr * EBL** | 2 | 1.91^ns^ | 0.001^ns^ | 3.92^***^ | 11.09^ns^ | 6.72^ns^ | 0.61^ns^ | 1.64^ns^ | 3.26^*^ | 68.92^ns^ | 0.001^**^ |
| **Y* Irr *ML** | 2 | 0.19^ns^ | 0.005^ns^ | 0.21^ns^ | 30.26^***^ | 2.61^ns^ | 0.15^ns^ | 0.71^ns^ | 1.28^ns^ | 2.009^ns^ | 0.0003^ns^ |
| **Y* EBL *ML** | 4 | 0.28^ns^ | 0.003^ns^ | 0.21^ns^ | 1.85^ns^ | 0.78^ns^ | 0.01^ns^ | 0.02^ns^ | 0.34^ns^ | 6.38^ns^ | 0.0007^**^ |
| **Irr * EBL *ML** | 4 | 0.05^ns^ | 0.006^ns^ | 0.55^ns^ | 6.07^ns^ | 0.95^ns^ | 0.24^ns^ | 1.06^ns^ | 0.25^ns^ | 12.76^ns^ | 0.0005^**^ |
| **Y* Irr * EBL *ML** | 4 | 0.99^ns^ | 0.002^ns^ | 0.21^ns^ | 6.68^ns^ | 1.85^ns^ | 0.58^ns^ | 0.28^ns^ | 0.09^ns^ | 24.43^ns^ | 0.0006^**^ |
| **Error** | 64 | 0.0089 | 0.008 | 0.40 | 3.79 | 2.38 | 0.73 | 0.68 | 0.82 | 28.45 | 0.0001 |

**Irr:** Different levels of water deficit**, EBL:** Different concentrations of 24-epibrassinolide**, ML:** Different concentrations of melatonin, **PH:** Plant height, **NMB:** Number of main branches, **NLB:** Number of lateral branches, **NSP:** Number of seeds per plant, **NPP:** Number of pods per plant, **PWP:** Pod weight per plant, **W100S:** 100-seed weight, **W100P:** 100-pod weight, **SSY:** Straw and stubble yield, **HI:** Harvest index.

* and ** Significantly at the probability level of %5 and %1, respectively.

**Table S1** (continued).

| **SOV** | **df** | **MBD** | **PSP** | **GY** | **SC** | **Chl a** | **Chl b** | **Tot chl** | **Cart** | **MDA** | **EL** |
| --- | --- | --- | --- | --- | --- | --- | --- | --- | --- | --- | --- |
| **Year (Y)** | 1 | 12.27^***^ | 46.30^***^ | 4667067.35^***^ | 116020.09^***^ | 44.65^***^ | 7.73^***^ | 89.54^***^ | 17.35^***^ | 43.08^***^ | 2110.17^***^ |
| **Y*Rep** | 4 | 0.007^ns^ | 0.14^ns^ | 1192.93^ns^ | 56.11^ns^ | 0.17 ^ns^ | 0.00^ns^ | 0.23^ns^ | 0.17^*^ | 0.05^ns^ | 0.04^ns^ |
| **Irr** | 1 | 24.03^***^ | 71.47^***^ | 9556853.64^***^ | 197568.48^***^ | 563.84^***^ | 160.46^***^ | 1325.90^***^ | 218.77^***^ | 22.00^***^ | 313.11^***^ |
| **Y* Irr** | 1 | 0.11^*^ | 0.01^ns^ | 978810.93^***^ | 12921.40^***^ | 4.21^*^ | 1.05^***^ | 9.48^*^ | 1.44^**^ | 1.14^*^ | 5.60^*^ |
| **Y* Irr *Rep** | 4 | 0.008^ns^ | 0.08^ns^ | 747.95^ns^ | 161.91^**^ | 0.49^**^ | 0.00^ns^ | 0.52^**^ | 0.06^ns^ | 0.06^ns^ | 0.71^ns^ |
| **EBL** | 2 | 3.43^***^ | 31.80^***^ | 494065.32^***^ | 10320.85^***^ | 25.82^***^ | 6.13^***^ | 56.99^***^ | 9.17^***^ | 23.87^***^ | 767.24^***^ |
| **ML** | 2 | 2.91^***^ | 26.11^***^ | 352445.99^***^ | 8202.89^***^ | 21.03^***^ | 3.90^***^ | 43.02^***^ | 11.56^***^ | 19.65^***^ | 659.81^***^ |
| **Y*EBL** | 2 | 0.02^ns^ | 0.26^ns^ | 14107.49^**^ | 314.91^***^ | 0.17^ns^ | 0.04^ns^ | 0.36^ns^ | 0.18^ns^ | 0.12^ns^ | 14.89^***^ |
| **Y*ML** | 2 | 0.02^ns^ | 0.17^ns^ | 1454.71^ns^ | 59.61^ns^ | 0.08^ns^ | 0.02^ns^ | 0.19^ns^ | 0.01^ns^ | 0.12^ns^ | 10.82^***^ |
| **Irr * EBL** | 2 | 0.05^**^ | 3.09^***^ | 26234.15^***^ | 142.86^*^ | 1.43^***^ | 1.17^***^ | 5.19^***^ | 0.02^ns^ | 12.39^***^ | 522.15^***^ |
| **Irr *ML** | 2 | 0.02^ns^ | 1.67^***^ | 3489.53^ns^ | 54.63^ns^ | 0.90^***^ | 0.64^***^ | 3.05^***^ | 0.01^ns^ | 10.07^***^ | 459.34^***^ |
| **EBL *ML** | 4 | 0.02^ns^ | 0.04^ns^ | 19119.22^***^ | 79.03^ns^ | 0.28^*^ | 0.10^ns^ | 0.62^**^ | 0.18^*^ | 0.95^**^ | 10.32^***^ |
| **Y* Irr * EBL** | 2 | 0.02^ns^ | 0.01^ns^ | 10084.02^**^ | 48.83^ns^ | 0.05^ns^ | 0.00^ns^ | 0.10^ns^ | 0.00^ns^ | 0.16^ns^ | 9.20^**^ |
| **Y* Irr *ML** | 2 | 0.01^ns^ | 0.05^ns^ | 3282.05^ns^ | 11.34^ns^ | 0.00 ^ns^ | 0.00^ns^ | 0.02^ns^ | 0.00^ns^ | 0.11^ns^ | 6.53^**^ |
| **Y* EBL *ML** | 4 | 0.003^ns^ | 0.07^ns^ | 1321.17^ns^ | 4.6614^ns^ | 0.01^ns^ | 0.00^ns^ | 0.01^ns^ | 0.01^ns^ | 0.00^ns^ | 0.13^ns^ |
| **Irr * EBL *ML** | 4 | 0.02^ns^ | 0.02^ns^ | 3871.98^ns^ | 40.05^ns^ | 0.16^ns^ | 0.06^ns^ | 0.19^ns^ | 0.00 ^ns^ | 0.36^ns^ | 8.31^***^ |
| **Y* Irr * EBL *ML** | 4 | 0.01^ns^ | 0.07^ns^ | 1446.49^ns^ | 17.92^ns^ | 0.01^ns^ | 0.00^ns^ | 0.01^ns^ | 0.00^ns^ | 0.00^ns^ | 0.06^ns^ |
| **Error** | 64 | 0.009 | 0.14 | 1952.75 | 35.82 | 0.10 | 0.06 | 0.13 | 0.06 | 0.188 | 1.29 |

**Irr:** Different levels of water deficit**, EBL:** Different concetrations of 24-epibrassinolide**, ML:** Different concetrations of melatonin, **MBD:** Main branch diameter, **PSP:** Percentage of seedless pods, **GY:** Grain Yield, **SC**: Stomatal Conductance, **Chl a:** Chlorophyll a, **Chl b:** Chlorophyll b, **Tot chl:** Total chlorophyll, **Cart:** Carotenoid, **MDA**: Malondialdehyde, **EL**: Electrolyte leakage,

* and ** Significantly at the probability level of %5 and %1, respectively.

**Table S1** (continued).

| **SOV** | **df** | **CAT** | **SOD** | **GPX** | **APX** | **PPO** | **So pro** | **Prol** | **Flavo** | **Phe** |
| --- | --- | --- | --- | --- | --- | --- | --- | --- | --- | --- |
| **Year (Y)** | 1 | 0.0006^***^ | 0.0010^***^ | 0.00099^***^ | 0.0018^***^ | 0.0052^***^ | 1124.55^***^ | 1908.44^***^ | 170.61*** | 294.42*** |
| **Y*Rep** | 4 | 0.0000005^ns^ | 0.00000044^ns^ | 0.0000015^ns^ | 0.0000016^ns^ | 0.0000025^*^ | 0.27^ns^ | 2.41^*^ | 0.09ns | 0.61ns |
| **Irr** | 1 | 0.0044^***^ | 0.018^***^ | 0.0046^***^ | 0.0047^***^ | 0.0077^***^ | 2454.83^***^ | 6271.01^***^ | 1673.98*** | 3800.66*** |
| **Y* Irr** | 1 | 0.00001^**^ | 0.000050^***^ | 0.000022^**^ | 0.000031^***^ | 0.000094^**^ | 6.80^**^ | 63.11^***^ | 2.80ns | 12.42** |
| **Y* Irr *Rep** | 4 | 0.0000006^ns^ | 0.00000043^ns^ | 0.00000031^ns^ | 0.00000021^ns^ | 0.0000013^ns^ | 0.17^ns^ | 0.35^ns^ | 0.71ns | 0.50ns |
| **EBL** | 2 | 0.0012^***^ | 0.0018^***^ | 0.0013^***^ | 0.0010^***^ | 0.0012^***^ | 1689.61^***^ | 1428.01^***^ | 534.17*** | 654.49*** |
| **ML** | 2 | 0.0012^***^ | 0.0015^***^ | 0.0012^***^ | 0.00092^***^ | 0.0010^***^ | 1361.20^***^ | 1267.84^***^ | 462.08*** | 463.67*** |
| **Y*EBL** | 2 | 0.000006^*^ | 0.0000060^**^ | 0.00000515^*^ | 0.0000070^***^ | 0.0000095^***^ | 4.68^ns^ | 11.74^***^ | 4.02*** | 2.08** |
| **Y*ML** | 2 | 0.000002^ns^ | 0.0000024^ns^ | 0.000012^***^ | 0.0000060^**^ | 0.000011^***^ | 3.77^ns^ | 13.91^***^ | 1.99* | 8.03*** |
| **Irr * EBL** | 2 | 0.000006^*^ | 0.00016^***^ | 0.0000083^**^ | 0.0000040^*^ | 0.0000020^ns^ | 79.15^***^ | 31.72^***^ | 15.59*** | 21.36*** |
| **Irr *ML** | 2 | 0.000001^ns^ | 0.00015^***^ | 0.0000031^ns^ | 0.0000028^*^ | 0.00000077^ns^ | 61.20^***^ | 4.89^**^ | 18.60*** | 19.49*** |
| **EBL *ML** | 4 | 0.000007^**^ | 0.000015^***^ | 0.0000025^ns^ | 0.00000060^ns^ | 0.000013^***^ | 11.66^***^ | 3.03^**^ | 4.40*** | 10.46*** |
| **Y* Irr * EBL** | 2 | 0.00000001^ns^ | 0.0000012^ns^ | 0.00000098^ns^ | 0.00000003^ns^ | 0.0000045^**^ | 0.21^ns^ | 0.53^ns^ | 0.78ns | 0.56ns |
| **Y* Irr *ML** | 2 | 0.0000001^ns^ | 0.00000069^ns^ | 0.00000097^ns^ | 0.00000002^ns^ | 0.0000010^ns^ | 0.16^ns^ | 1.94^ns^ | 0.26ns | 0.00ns |
| **Y* EBL *ML** | 4 | 0.0000004^ns^ | 0.00000051^ns^ | 0.00000095^ns^ | 0.00000000^ns^ | 0.00000018^ns^ | 0.03^ns^ | 0.86^ns^ | 0.24ns | 0.79ns |
| **Irr * EBL *ML** | 4 | 0.000005^*^ | 0.0000043^**^ | 0.0000023^ns^ | 0.0000091^***^ | 0.0000014^ns^ | 10.96^***^ | 8.49^***^ | 0.81ns | 1.43** |
| **Y* Irr * EBL *ML** | 4 | 0.00000002^ns^ | 0.00000031^ns^ | 0.0000014^ns^ | 0.00000006^ns^ | 0.0000010^ns^ | 0.030^ns^ | 0.86^ns^ | 0.30ns | 0.21ns |
| **Error** | 64 | 0.00000160 | 0.00000114 | 0.0000011 | 0.00000084 | 0.00000073 | 1.49 | 0.71 | 0.41 | 0.36 |

**Irr:** Different levels of water deficit**, EBL:** Different concetrations of 24-epibrassinolide**, ML:** Different concetrations of melatonin, **CAT:** Catalase, **SOD:** Superoxide dismutase, **GPX:** Guaiacol peroxidase, **APX:** Ascorbate peroxidase, **PPO:** Polyphenol oxidase, **So Pro**: Total soluble protein, **Prol**: Proline, **Flavo**: Flavonoid, **Phe**: Phenol, **Sug**: Sugar.

* and ** Significantly at the probability level of %5 and %1, respectively.

| **SOV** | **df** | **Phe** | **Sug** | **H_2_O_2_** | **RWC** | **%N** | **%Pro** | **%Oil** | **SOY** | **SPY** |
| --- | --- | --- | --- | --- | --- | --- | --- | --- | --- | --- |
| **Year (Y)** | 1 | 294.42*** | 147.00^***^ | 49.47^***^ | 988.21^***^ | 49.33^***^ | 1927.03^***^ | 150.56^***^ | 64093.47^***^ | 641247.05^***^ |
| **Y*Rep** | 4 | 0.61ns | 0.12^ns^ | 0.46^ns^ | 1.28^ns^ | 0.01^ns^ | 0.56^ns^ | 0.10^ns^ | 31.28^ns^ | 60.29^ns^ |
| **Irr** | 1 | 3800.66*** | 511.13^***^ | 527.57^***^ | 21132.51^***^ | 6.57^***^ | 256.86^***^ | 34.43^***^ | 60551.79^***^ | 170238.31^***^ |
| **Y* Irr** | 1 | 12.42** | 4.96^***^ | 4.36^**^ | 26.07^*^ | 0.05^ns^ | 2.03^ns^ | 0.01^ns^ | 10733.45^***^ | 78629.03^***^ |
| **Y* Irr *Rep** | 4 | 0.50ns | 0.03^ns^ | 0.15^ns^ | 1.24^ns^ | 0.02^ns^ | 1.02^ns^ | 0.20^ns^ | 50.69^ns^ | 118.50^ns^ |
| **EBL** | 2 | 654.49*** | 237.82^***^ | 29.52^***^ | 341.94^***^ | 1.90^***^ | 74.56^***^ | 5.64^***^ | 3838.58^***^ | 34871.01^***^ |
| **ML** | 2 | 463.67*** | 214.93^***^ | 22.61^***^ | 295.01^***^ | 1.72^***^ | 67.40^***^ | 4.88^***^ | 2770.63^***^ | 26193.23^***^ |
| **Y*EBL** | 2 | 2.08** | 1.82^***^ | 0.40^ns^ | 1.38^ns^ | 0.06^ns^ | 2.42^ns^ | 0.45^*^ | 518.88^***^ | 2783.69^***^ |
| **Y*ML** | 2 | 8.03*** | 4.01^***^ | 0.11^ns^ | 2.25^ns^ | 0.03^ns^ | 1.32^ns^ | 0.28^ns^ | 265.64^**^ | 1608.68^***^ |
| **Irr * EBL** | 2 | 21.36*** | 14.11^***^ | 2.74^***^ | 15.45^***^ | 0.01^ns^ | 0.74^ns^ | 0.04^ns^ | 316.60^***^ | 1464.16^***^ |
| **Irr *ML** | 2 | 19.49*** | 13.01^***^ | 3.23^***^ | 11.55^***^ | 0.03^ns^ | 1.55^ns^ | 0.002^ns^ | 144.43^*^ | 552.48^*^ |
| **EBL *ML** | 4 | 10.46*** | 3.21^***^ | 0.04^ns^ | 7.77^***^ | 2.73^***^ | 106.99^***^ | 4.31^***^ | 532.13^***^ | 12809.10^***^ |
| **Y* Irr * EBL** | 2 | 0.56ns | 0.14^ns^ | 0.03^ns^ | 19.97^***^ | 0.02^ns^ | 0.86^ns^ | 0.13^ns^ | 130.0006^*^ | 747.62^*^ |
| **Y* Irr *ML** | 2 | 0.00ns | 0.75^*^ | 0.00^ns^ | 26.31^***^ | 0.01^ns^ | 0.54^ns^ | 0.01^ns^ | 7.10^ns^ | 297.14^ns^ |
| **Y* EBL *ML** | 4 | 0.79ns | 0.44^ns^ | 0.06^ns^ | 2.41^ns^ | 0.15^**^ | 5.96^**^ | 0.35^*^ | 93.61^ns^ | 2377.40^***^ |
| **Irr * EBL *ML** | 4 | 1.43** | 2.65^***^ | 0.18^ns^ | 0.96^ns^ | 0.02^ns^ | 0.92^ns^ | 0.05^ns^ | 65.88^ns^ | 653.61^**^ |
| **Y* Irr * EBL *ML** | 4 | 0.21ns | 0.32^ns^ | 0.22^ns^ | 1.92^ns^ | 0.07^ns^ | 2.91^ns^ | 0.07^ns^ | 25.41^ns^ | 1061.27^***^ |
| **Error** | 64 | 0.36 | 0.22 | 0.29 | 1.17 | 0.03 | 1.21 | 0.13 | 40.37 | 159.72 |

**Table S1** (continued).

**Irr:** Different levels of water deficit**, EBL:** Different concetrations of 24-epibrassinolide**, ML:** Different concetrations of melatonin, **H_2_O_2:_** Hydrogen peroxide, **RW**C: Relative water content, **%N**: Seed nitrogen, **%Pro**: Seed protein, **%Oil:** Seed oil, **SOY:** Seed oil yield, **SPY:** Seed protein yield.

Columns with similar letters did not differ significantly.

**Table S2.** The mean comparison of simple effect of year on evaluated traits of chickpea plants

| **Year (Y)** | **PH** | **NMB** | **NLB** | **NSP** | **NPP** | **PWP** | **W100S** | **W100P** | **SSY** | **HI** | **MBD** | **PSP** | **GY** |
| --- | --- | --- | --- | --- | --- | --- | --- | --- | --- | --- | --- | --- | --- |
| **1** | 49.80 a | 3.11 a | 23.95 a | 61.56 a | 44.89 a | 22.32 a | 38.58 a | 45.05 a | 116.07 b | 0.73 a | 4.67 a | 5.78 b | 1220.98 a |
| **2** | 44.87 b | 2.82 b | 19.19 b | 46.78 b | 32.66 b | 16.44 b | 33.79 b | 37.71 b | 158.61 a | 0.59 b | 4.00 b | 7.09 a | 805.22 b |

**Table S2** (continued).

| **Year (Y)** | **SC** | **Chl a** | **Chl b** | **Tot chl** | **Cart** | **MDA** | **EL** | **CAT** | **SOD** | **GPX** | **APX** | **PPO** |
| --- | --- | --- | --- | --- | --- | --- | --- | --- | --- | --- | --- | --- |
| **1** | 253.13 a | 8.88 a | 3.56 a | 12.45 a | 5.98 a | 6.99 b | 29.26 b | 0.048 a | 0.06 a | 0.04 b | 0.05 a | 0.07 a |
| **2** | 187.58 b | 7.59 b | 3.03 b | 10.63 b | 5.18 b | 8.26 a | 38.10 a | 0.044 b | 0.05 b | 0.05 a | 0.04 b | 0.06 b |

**Table S2** (continued).

| **Year (Y)** | **So pro** | **Prol** | **Flavo** | **Phe** | **Sug** | **H_2_O_2_** | **RWC** | **N** | **Pro** | **Oil** | **SOY** | **SPY** |
| --- | --- | --- | --- | --- | --- | --- | --- | --- | --- | --- | --- | --- |
| **1** | 64.53 a | 35.73 b | 18.03 b | 28.13 a | 9.44 b | 8.42 b | 65.27 a | 3.41 a | 21.33 a | 6.83 a | 86.25 a | 255.88 a |
| **2** | 64.53 a | 44.14 a | 20.55 a | 24.83 b | 11.77 a | 9.77 a | 59.22 b | 2.06 b | 12.88 b | 4.47 b | 37.52 b | 101.77 b |

**PH:** Plant height, **NMB:** Number of main branches, **NLB:** Number of lateral branches, **NSP:** Number of seeds per plant, **NPP:** Number of pods per plant, **PWP:** Pod weight per plant, **W100S:** 100-seed weight, **W100P:** 100-pod weight, **SSY:** Straw and stubble yield, **HI:** Harvest index, **MBD:** Main branch diameter, **PSP:** Percentage of seedless pods, **GY:** Grain Yield, **SC**: Stomatal Conductance, **Chl a:** Chlorophyll a, **Chl b:** Chlorophyll b, **Tot Chl:** Total chlorophyll, **Cart:** Carotenoid, **MDA**: Malondialdehyde, **EL**: Electrolyte leakage, **CAT:** Catalase, **SOD:** Superoxide dismutase, **GPX:** Guaiacol peroxidase, **APX:** Ascorbate peroxidase, **PPO:** Polyphenol oxidase, **Tot Pro**: Total soluble protein, **Prol**: Proline, **Flavo**: Flavonoid, **Phe**: Phenol, **Sug**: Sugar, **RW**C: Relative water content, **%N**: Seed nitrogen, **%Pro**: Seed protein, **%Oil:** Seed oil, **SOY:** Seed oil yield, **SPY:** Seed protein yield.

Mean comparison was performed by duncan method at 5% probability. Columns with similar letters did not differ significantly.

**Table S3.** The mean comparison of simple effect of various levels of irrigation on evaluated traits of chickpea plants

| **Irrigation (Irr)** | PH | NMB | NLB | NSP | NPP | PWP | W100S | W100P | SSY | HI | MBD | PSP | GY |
| --- | --- | --- | --- | --- | --- | --- | --- | --- | --- | --- | --- | --- | --- |
| **1** | 49.39 a | 3.28 a | 22.76 a | 64.91 a | 46.51 a | 23.95 a | 39.75 a | 44.18 a | 151.42 a | 0.68 a | 4.80 a | 5.62 b | 1310.57 a |
| **2** | 45.28 b | 2.65 b | 20.37 b | 43.44 b | 31.04 b | 14.81 b | 32.62 b | 38.59 b | 123.25 b | 0.64 b | 3.86 b | 7.25 a | 715.63 b |

**Table S3** (continued).

| **Irrigation (Irr)** | **SC** | **Chl a** | **Chl b** | **Tot Chl** | **Cart** | **MDA** | **EL** | **CAT** | **SOD** | **GPX** | **APX** | **PPO** |
| --- | --- | --- | --- | --- | --- | --- | --- | --- | --- | --- | --- | --- |
| **1** | 263.13 a | 10.52 a | 4.51 a | 15.04 a | 7.00 a | 7.17 b | 31.98 b | 0.03 b | 0.04 b | 0.04 b | 0.04 b | 0.05 b |
| **2** | 177.59 b | 5.95 b | 2.08 b | 8.03 b | 4.16 b | 9.98 a | 45.38 a | 0.52 a | 0.07 a | 0.05 a | 0.07 a | 0.07 a |

**Table S3** (continued).

| **Irrigation (Irr)** | **So pro** | **Prol** | **Flavo** | **Phe** | **Sug** | **H_2_O_2_** | **RWC** | **N** | **Pro** | **Oil** | **SOY** | **SPY** |
| --- | --- | --- | --- | --- | --- | --- | --- | --- | --- | --- | --- | --- |
| **1** | 56.54 b | 32 b | 15.35 b | 20.55 b | 8.43 b | 6.88 b | 76.23 a | 2.49 b | 15.56 b | 6.22 a | 85.56 a | 218.52 a |
| **2** | 78 a | 47.56 a | 23.23 a | 32.41 a | 12.78 a | 11.30 a | 48.26 b | 2.98 a | 18.65 a | 5.09 b | 38.21 b | 139.12 b |

**PH:** Plant height, **NMB:** Number of main branches, **NLB:** Number of lateral branches, **NSP:** Number of seeds per plant, **NPP:** Number of pods per plant, **PWP:** Pod weight per plant, **W100S:** 100-seed weight, **W100P:** 100-pod weight, **SSY:** Straw and stubble yield, **HI:** Harvest index, **MBD:** Main branch diameter, **PSP:** Percentage of seedless pods, **GY:** Grain Yield, **SC**: Stomatal Conductance, **Chl a:** Chlorophyll a, **Chl b:** Chlorophyll b, **Tot Chl:** Total chlorophyll, **Cart:** Carotenoid, **MDA**: Malondialdehyde, **EL**: Electrolyte leakage, **CAT:** Catalase, **SOD:** Superoxide dismutase, **GPX:** Guaiacol peroxidase, **APX:** Ascorbate peroxidase, **PPO:** Polyphenol oxidase, **Tot Pro**: Total soluble protein, **Prol**: Proline, **Flavo**: Flavonoid, **Phe**: Phenol, **Sug**: Sugar, **RW**C: Relative water content, **%N**: Seed nitrogen, **%Pro**: Seed protein, **%Oil:** Seed oil, **SOY:** Seed oil yield, **SPY:** Seed protein yield.

Mean comparison was performed by DUNCAN method at 5% probability. Columns with similar letters did not differ significantly.

**Table S4.** The mean comparison of simple effect of different 24-epibrassinolide levels on evaluated traits of chickpea plants

| **24-epibrassinolide (EBL)** | **PH** | **NMB** | **NLB** | **NSP** | **NPP** | **PWP** | **W100S** | **W100P** | **SSY** | **HI** | **MBD** | **PSP** | **GY** |
| --- | --- | --- | --- | --- | --- | --- | --- | --- | --- | --- | --- | --- | --- |
| **0** | 45.97 c | 2.80 c | 20.52 c | 49.86 c | 36.07 c | 17.98 c | 34.70 c | 39.70 c | 125.97 c | 0.67 a | 4.03 c | 7.38 a | 893.39 c |
| **1** | 47.56 b | 2.93 b | 21.73 b | 54.83 b | 38.91 b | 19.43 b | 36.04 b | 41.38 b | 136.86 b | 0.66 b | 4.33 b | 6.42 b | 1018.41 b |
| **2** | 48.48 a | 3.16 a | 22.45 a | 57.82 a | 41.34 a | 20.73 a | 37.82 a | 43.07 a | 149.19 a | 0.66 b | 4.64 a | 5.50 c | 1127.50 a |

**Table S4** (continued).

| **24-epibrassinolide (EBL)** | **SC** | **Chl a** | **Chl b** | **Tot Chl** | **Cart** | **MDA** | **EL** | **CAT** | **SOD** | **GPX** | **APX** | **PPO** |
| --- | --- | --- | --- | --- | --- | --- | --- | --- | --- | --- | --- | --- |
| **0** | 203.85 c | 7.40 c | 2.86 c | 10.26 c | 5.08 c | 8.47 a | 38.42 a | 0.04 c | 0.05 c | 0.04 c | 0.04 c | 0.06 c |
| **1** | 219.54 b | 8.23 b | 3.34 b | 11.57 b | 5.57 b | 7.57 b | 33.42 b | 0.04 b | 0.06 b | 0.04 b | 0.04 b | 0.06 b |
| **2** | 237.68 a | 9.09 a | 3.68 a | 12.78 a | 6.09 a | 6.84 c | 29.20 c | 0.05 a | 0.06 a | 0.05 a | 0.05 a | 0.07 a |

**Table S4** (continued).

| **24-epibrassinolide (EBL)** | **So pro** | **Prol** | **Flavo** | **Phe** | **Sug** | **H_2_O_2_** | **RWC** | **N** | **Pro** | **Oil** | **SOY** | **SPY** |
| --- | --- | --- | --- | --- | --- | --- | --- | --- | --- | --- | --- | --- |
| **0** | 54.94 c | 34.48 c | 15.84 c | 22.75 c | 8.26 c | 10.00 a | 59.05 c | 2.47 c | 15.48 c | 5.20 b | 50.38 c | 143.31 c |
| **1** | 60.43 b | 38.50 b | 18.58 b | 25.56 b | 10.21 b | 9.10 b | 62.49 b | 2.91 a | 18.22 a | 5.91 a | 64.94 b | 191.78 b |
| **2** | 68.55 a | 46.83 a | 23.45 a | 31.13 a | 13.35 a | 8.18 c | 65.20 a | 2.81 b | 17.60 b | 5.86 a | 70.34 a | 201.37 c |

**PH:** Plant height, **NMB:** Number of main branches, **NLB:** Number of lateral branches, **NSP:** Number of seeds per plant, **NPP:** Number of pods per plant, **PWP:** Pod weight per plant, **W100S:** 100-seed weight, **W100P:** 100-pod weight, **SSY:** Straw and stubble yield, **HI:** Harvest index, **MBD:** Main branch diameter, **PSP:** Percentage of seedless pods, **GY:** Grain Yield, **SC**: Stomatal Conductance, **Chl a:** Chlorophyll a, **Chl b:** Chlorophyll b, **Tot Chl:** Total chlorophyll, **Cart:** Carotenoid, **MDA**: Malondialdehyde, **EL**: Electrolyte leakage, **CAT:** Catalase, **SOD:** Superoxide dismutase, **GPX:** Guaiacol peroxidase, **APX:** Ascorbate peroxidase, **PPO:** Polyphenol oxidase, **Tot Pro**: Total soluble protein, **Prol**: Proline, **Flavo**: Flavonoid, **Phe**: Phenol, **Sug**: Sugar, **RW**C: Relative water content, **%N**: Seed nitrogen, **%Pro**: Seed protein, **%Oil:** Seed oil, **SOY:** Seed oil yield, **SPY:** Seed protein yield.

Mean comparison was performed by DUNCAN method at 5% probability. Columns with similar letters did not differ significantly.

**Table S5.** The mean comparison of simple effect of different melatonin levels on evaluated traits of chickpea plants

| **Melatonin (ML)** | **PH** | **NMB** | **NLB** | **NSP** | **NPP** | **PWP** | **W100S** | **W100P** | **SSY** | **HI** | **MBD** | **PSP** | **GY** |
| --- | --- | --- | --- | --- | --- | --- | --- | --- | --- | --- | --- | --- | --- |
| **0** | 45.96 c | 2.82 c | 20.76 c | 50.75 c | 36.32 c | 18.15 c | 34.88 c | 39.76 c | 127.41 c | 0.67 a | 4.05 c | 7.30 a | 915.55 c |
| **1** | 47.41 b | 2.96 b | 21.59 b | 54.33 b | 38.79 b | 19.34 b | 36.03 b | 41.40 b | 136.66 b | 0.67 a | 4.33 b | 6.41 b | 1010.37 b |
| **2** | 48.64 a | 3.11 a | 22.35 a | 57.43 a | 41.21 a | 20.65 a | 37.65 a | 42.99 a | 147.94 a | 0.65 b | 4.62 a | 5.60 c | 1113.38 a |

**Table S5** (continued).

| **Melatonin (ML)** | **SC** | **Chl a** | **Chl b** | **Tot Chl** | **Cart** | **MDA** | **EL** | **CAT** | **SOD** | **GPX** | **APX** | **PPO** |
| --- | --- | --- | --- | --- | --- | --- | --- | --- | --- | --- | --- | --- |
| **0** | 205.40 c | 7.47 c | 2.98 c | 10.45 c | 5.04 c | 8.34 a | 37.76 a | 0.04 c | 0.05 c | 0.04 c | 0.04 c | 0.06 c |
| **1** | 220.08 b | 8.25 b | 3.28 b | 11.53 b | 5.53 b | 7.68 b | 34.07 b | 0.04 b | 0.06 b | 0.04 b | 0.04 b | 0.06 b |
| **2** | 235.59 a | 9.00 a | 3.63 a | 12.63 a | 6.17 a | 6.86 c | 29.22 c | 0.05 a | 0.06 a | 0.05 a | 0.05 a | 0.07 a |

**Table S5** (continued).

| **Melatonin (ML)** | **So pro** | **Prol** | **Flavo** | **Phe** | **Sug** | **H_2_O_2_** | **RWC** | **N** | **Pro** | **Oil** | **SOY** | **SPY** |
| --- | --- | --- | --- | --- | --- | --- | --- | --- | --- | --- | --- | --- |
| **0** | 55.68 c | 34.38 c | 15.94 c | 23.07 c | 8.27 c | 9.86 a | 59.33 c | 2.49 c | 15.59 c | 5.23 b | 52.03 c | 147.90 c |
| **1** | 60.37 b | 39.24 b | 18.87 b | 26.14 b | 10.40 b | 9.14 b | 62.36 b | 2.92 a | 18.25 a | 5.92 a | 64.80 b | 191.07 b |
| **2** | 67.87 a | 46.19 a | 23.07 a | 30.23 a | 13.14 a | 8.28 c | 65.05 a | 2.79 b | 17.48 b | 5.81 a | 68.83 a | 197.50 a |

**PH:** Plant height, **NMB:** Number of main branches, **NLB:** Number of lateral branches, **NSP:** Number of seeds per plant, **NPP:** Number of pods per plant, **PWP:** Pod weight per plant, **W100S:** 100-seed weight, **W100P:** 100-pod weight, **SSY:** Straw and stubble yield, **HI:** Harvest index, **MBD:** Main branch diameter, **PSP:** Percentage of seedless pods, **GY:** Grain Yield, **SC**: Stomatal Conductance, **Chl a:** Chlorophyll a, **Chl b:** Chlorophyll b, **Tot Chl:** Total chlorophyll, **Cart:** Carotenoid, **MDA**: Malondialdehyde, **EL**: Electrolyte leakage, **CAT:** Catalase, **SOD:** Superoxide dismutase, **GPX:** Guaiacol peroxidase, **APX:** Ascorbate peroxidase, **PPO:** Polyphenol oxidase, **Tot Pro**: Total soluble protein, **Prol**: Proline, **Flavo**: Flavonoid, **Phe**: Phenol, **Sug**: Sugar, **RW**C: Relative water content, **%N**: Seed nitrogen, **%Pro**: Seed protein, **%Oil:** Seed oil, **SOY:** Seed oil yield, **SPY:** Seed protein yield.

Mean comparison was performed by DUNCAN method at 5% probability. Columns with similar letters did not differ significantly.

**Table S6.** The mean comparison of interaction effects of different levels of water deficit and year crops on evaluated traits of chickpea plants

| **Teatment** | | **PH** | **NMB** | **NLB** | **NSP** | **NPP** | **PWP** | **W100S** | **W100P** | **SSY** | **HI** | **MBD** | **PSP** | **GY** |
| --- | --- | --- | --- | --- | --- | --- | --- | --- | --- | --- | --- | --- | --- | --- |
| **Year** | **Irr** |  |  |  |  |  |  |  |  |  |  |  |  |  |
| **1** | **1** | 52.37 a | 3.37 a | 25.04 a | 74.82 a | 56.82 a | 27.17 a | 43.02 a | 47.82 a | 127.25 c | 0.75 a | 5.11 a | 4.95 d | 1613.65 a |
|  | **2** | 47.23 b | 2.84 c | 22.85 b | 48.31 c | 32.97 c | 17.46 c | 34.15 c | 42.29 b | 104.88 d | 0.72 b | 4.23 c | 6.61 b | 828.31 c |
| **2** | **1** | 46.42 c | 3.18 b | 20.49 c | 55.00 b | 36.21 b | 20.73 b | 36.49 b | 40.54 c | 175.59 a | 0.61 c | 4.50 b | 6.29 c | 1007.49 b |
|  | **2** | 43.33 d | 2.46 d | 17.88 d | 38.57 d | 29.11 d | 12.16 d | 31.10 d | 34.88 d | 141.63 b | 0.57 d | 3.49 d | 7.89 a | 602.95 d |

**Table S6** (continued).

| **Teatment** | | **SC** | **Chl a** | **Chl b** | **Tot Chl** | **Cart** | **MDA** | **EL** | **CAT** | **SOD** | **GPX** | **APX** | **PPO** |
| --- | --- | --- | --- | --- | --- | --- | --- | --- | --- | --- | --- | --- | --- |
| **Year** | **Irr** |  |  |  |  |  |  |  |  |  |  |  |  |
| **1** | **1** | 306.84 a | 11.36 a | 4.88 a | 16.25 a | 7.52 a | 6.64 d | 27.79 d | 0.04 c | 0.05 c | 0.03 d | 0.04 c | 0.06 c |
|  | **2** | 199.42 c | 6.40 c | 2.24 c | 8.65 c | 4.44 c | 7.34 c | 30.74 c | 0.05 a | 0.07 a | 0.05 b | 0.06 a | 0.08 a |
| **2** | **1** | 219.41 b | 9.68 b | 40.15 b | 13.83 b | 6.49 b | 7.70 b | 36.17 b | 0.03 d | 0.04 d | 0.04 c | 0.04 d | 0.05 d |
|  | **2** | 155.75 d | 5.51 d | 1.91 d | 7.42 d | 3.87 d | 8.81 a | 40.03 a | 0.05 b | 0.07 b | 0.05 a | 0.05 b | 0.06 b |

**Table S6** (continued).

| **Teatment** | | **So pro** | **Prol** | **Flavo** | **Phe** | **Sug** | **H_2_O_2_** | **RWC** | **N** | **Pro** | **Oil** | **SOY** | **SPY** |
| --- | --- | --- | --- | --- | --- | --- | --- | --- | --- | --- | --- | --- | --- |
| **Year** | **Irr** |  |  |  |  |  |  |  |  |  |  |  |  |
| **1** | **1** | 59.51 c | 28.88 d | 14.26 d | 21.86 c | 7.48 d | 6.41 d | 79.75 a | 3.18 b | 19.92 b | 7.39 a | 119.89 a | 322.56 a |
|  | **2** | 69.55 a | 42.59 b | 21.81 b | 34.40 a | 11.40 b | 10.42 b | 50.79 c | 3.63 a | 22.73 a | 6.28 b | 52.60 b | 189.19 b |
| **2** | **1** | 53.56 d | 35.75 c | 16.45 c | 19.24 d | 9.38 c | 7.36 c | 72.72 b | 1.79 d | 11.20 d | 5.05 c | 51.23 b | 114.49 c |
|  | **2** | 62.60 b | 52.52 a | 24.65 a | 30.42 b | 14.16 a | 12.18 a | 45.72 d | 2.33 c | 14.56 c | 3.90 d | 23.81 c | 89.05 d |

**PH:** Plant height, **NMB:** Number of main branches, **NLB:** Number of lateral branches, **NSP:** Number of seeds per plant, **NPP:** Number of pods per plant, **PWP:** Pod weight per plant, **W100S:** 100-seed weight, **W100P:** 100-pod weight, **SSY:** Straw and stubble yield, **HI:** Harvest index, **MBD:** Main branch diameter, **PSP:** Percentage of seedless pods, **GY:** Grain Yield, **SC**: Stomatal Conductance, **Chl a:** Chlorophyll a, **Chl b:** Chlorophyll b, **Tot Chl:** Total chlorophyll, **Cart:** Carotenoid, **MDA**: Malondialdehyde, **EL**: Electrolyte leakage, **CAT:** Catalase, **SOD:** Superoxide dismutase, **GPX:** Guaiacol peroxidase, **APX:** Ascorbate peroxidase, **PPO:** Polyphenol oxidase, **Tot Pro**: Total soluble protein, **Prol**: Proline, **Flavo**: Flavonoid, **Phe**: Phenol, **Sug**: Sugar, **RW**C: Relative water content, **%N**: Seed nitrogen, **%Pro**: Seed protein, **%Oil:** Seed oil, **SOY:** Seed oil yield, **SPY:** Seed protein yield.

Mean comparison was performed by DUNCAN method at 5% probability. Columns with similar letters did not differ significantly.

| **Treatment** | | **PH** | **NMB** | **NLB** | **NSP** | **NPP** | **PWP** | **W100S** | **W100P** | **SSY** | **HI** | **MBD** | **PSP** | **GY** |
| --- | --- | --- | --- | --- | --- | --- | --- | --- | --- | --- | --- | --- | --- | --- |
| **Year** | **EBL** |  |  |  |  |  |  |  |  |  |  |  |  |  |
| **1** | **0** | 48.21 c | 2.89 d | 22.58 c | 56.91 c | 42.13 c | 20.80 c | 37.05 c | 43.47 c | 107.27 f | 0.74 a | 4.33 c | 6.67 c | 1082.40 c |
|  | **1** | 50.08 b | 3.09 b | 24.14 b | 62.15 b | 45.15 b | 22.33 b | 38.47 b | 45.04 b | 114.72 e | 0.73 a | 4.68 b | 5.72 e | 1224.55 b |
|  | **2** | 51.11 a | 3.34 a | 25.11 a | 65.63 a | 47.40 a | 23.81 a | 40.23 a | 46.65 a | 126.22 d | 0.73 a | 4.99 a | 4.95 f | 1355.99 a |
| **2** | **0** | 43.74 f | 2.71 f | 18.46 f | 42.82 f | 30.02 f | 15.16 f | 32.35 f | 35.94 f | 144.66 c | 0.60 b | 3.72 e | 8.10 a | 704.37 f |
|  | **1** | 45.03 e | 2.77 e | 19.31 e | 47.51 e | 32.67 e | 16.53 e | 33.62 e | 37.71 e | 159.00 b | 0.59 c | 3.97 d | 7.11 b | 812.28 e |
|  | **2** | 45.86 d | 2.99 c | 19.79 d | 50.02 d | 35.28 d | 17.64 d | 35.41 d | 39.49 d | 172.16 a | 0.58 c | 4.29 c | 6.06 d | 899.01 d |

**Table S7.** The mean comparison of interaction effects of different levels of 24-epibrassinolide and year crops on evaluated traits of chickpea plants

**Table S7** (continued).

| **Treatment** | | **SC** | **Chl a** | **Chl b** | **Tot Chl** | **Cart** | **MDA** | **EL** | **CAT** | **SOD** | **GPX** | **APX** | **PPO** |
| --- | --- | --- | --- | --- | --- | --- | --- | --- | --- | --- | --- | --- | --- |
| **Year** | **EBL** |  |  |  |  |  |  |  |  |  |  |  |  |
| **1** | **0** | 233.62 c | 7.98 d | 3.10 d | 11.08 d | 5.41d | 7.78 c | 33.33 c | 0.042 d | 0.058 d | 0.039 e | 0.049 d | 0.069 c |
|  | **1** | 252.41 b | 8.85 b | 3.61 b | 12.46 b | 5.97 b | 6.94 e | 29.06 d | 0.048 c | 0.063 c | 0.044 d | 0.053 b | 0.073 b |
|  | **2** | 273.37 a | 9.81 a | 3.98 a | 13.80 a | 6.56 a | 6.27 f | 25.39 e | 0.05 a | 0.07 a | 0.051 b | 0.061 a | 0.081 a |
| **2** | **0** | 174.07 f | 6.81 f | 2.63 e | 9.45 f | 4.75 f | 9.16 a | 43.51 a | 0.03 e | 0.052 f | 0.045 d | 0.041 f | 0.055 f |
|  | **1** | 186.68 e | 7.60 e | 3.07 d | 10.67 e | 5.18 e | 8.20 b | 37.78 b | 0.043 d | 0.057 e | 0.050 c | 0.045 e | 0.060 a |
|  | **2** | 201.99 d | 8.37 c | 3.39 c | 11.76 c | 5.61 c | 7.41 d | 33.01 c | 0.049 b | 0.065 b | 0.058 a | 0.051 c | 0.066 d |

**Table S7** (continued).

| **Treatment** | | **So pro** | **Prol** | **Flavo** | **Phe** | **Sug** | **H_2_O_2_** | **RWC** | **N** | **Pro** | **Oil** | **SOY** | **SPY** |
| --- | --- | --- | --- | --- | --- | --- | --- | --- | --- | --- | --- | --- | --- |
| **Year** | **EBL** |  |  |  |  |  |  |  |  |  |  |  |  |
| **1** | **0** | 57.83 d | 30.76 f | 14.77 f | 24.28 d | 7.31 e | 9.25 c | 61.88 c | 3.17 c | 19.84 c | 6.27 b | 70.44 c | 210.22 c |
|  | **1** | 63.61 c | 34.44 e | 17.52 d | 27.06 c | 9.06 d | 8.37 e | 65.72 b | 3.61 a | 22.62 a | 7.20 a | 90.69 b | 273.56 b |
|  | **2** | 72.16 a | 42.00 c | 21.81 b | 33.06 a | 11.95 b | 7.63 f | 68.21 a | 3.44 b | 21.53 b | 7.03 a | 97.61 a | 283.84 a |
| **2** | **0** | 52.05 e | 38.20 d | 16.92 e | 21.22 e | 9.20 d | 10.75 a | 56.21e | 1.78 e | 11.12 e | 4.13 d | 30.31 e | 76.40 f |
|  | **1** | 57.25 d | 42.56 c | 19.63 c | 24.07 d | 11.36 b | 9.82 b | 59.27 d | 2.21 d | 13.83 d | 4.61 c | 39.19 d | 110.00 e |
|  | **2** | 64.95 b | 51.66 a | 25.09 a | 29.20 b | 14.75 a | 8.74 d | 62.18 c | 2.18 d | 13.68 d | 4.68 c | 43.07 d | 118.89 d |

**PH:** Plant height, **NMB:** Number of main branches, **NLB:** Number of lateral branches, **NSP:** Number of seeds per plant, **NPP:** Number of pods per plant, **PWP:** Pod weight per plant, **W100S:** 100-seed weight, **W100P:** 100-pod weight, **SSY:** Straw and stubble yield, **HI:** Harvest index, **MBD:** Main branch diameter, **PSP:** Percentage of seedless pods, **GY:** Grain Yield, **SC**: Stomatal Conductance, **Chl a:** Chlorophyll a, **Chl b:** Chlorophyll b, **Tot Chl:** Total chlorophyll, **Cart:** Carotenoid, **MDA**: Malondialdehyde, **EL**: Electrolyte leakage, **CAT:** Catalase, **SOD:** Superoxide dismutase, **GPX:** Guaiacol peroxidase, **APX:** Ascorbate peroxidase, **PPO:** Polyphenol oxidase, **Tot Pro**: Total soluble protein, **Prol**: Proline, **Flavo**: Flavonoid, **Phe**: Phenol, **Sug**: Sugar, **RW**C: Relative water content, **%N**: Seed nitrogen, **%Pro**: Seed protein, **%Oil:** Seed oil, **SOY:** Seed oil yield, **SPY:** Seed protein yield.

Mean comparison was performed by DUNCAN method at 5% probability. Columns with similar letters did not differ significantly.

**Table S8.** The mean comparison of interaction effects of different levels of melatonin and year crops on evaluated traits of chickpea plants

| **Treatment** | | **PH** | **NMB** | **NLB** | **NSP** | **NPP** | **PWP** | **W100S** | **W100P** | **SSY** | **HI** | **MBD** | **PSP** | **GY** |
| --- | --- | --- | --- | --- | --- | --- | --- | --- | --- | --- | --- | --- | --- | --- |
| **Year** | **ML** |  |  |  |  |  |  |  |  |  |  |  |  |  |
| **1** | **0** | 48.08 c | 2.96 c | 22.97 c | 58.39 c | 42.36 c | 21.13 c | 37.21 c | 43.61 c | 108.11 f | 0.74 a | 4.36 c | 6.58 c | 1117.80 c |
|  | **1** | 49.95 b | 3.10 b | 23.98 b | 61.66 b | 44.91 b | 22.28 b | 38.43 b | 44.97 b | 115.44 e | 0.74 a | 4.67 b | 5.74 e | 1216.99 b |
|  | **2** | 51.36 a | 3.26 a | 24.89 a | 64.64 a | 47.41 a | 23.55 a | 40.11 a | 46.58 a | 124.66 d | 0.72 b | 4.98 a | 5.02 f | 1328.16 a |
| **2** | **0** | 43.83 f | 2.68 e | 18.55 f | 43.12 f | 30.28 f | 15.17 f | 32.54 f | 35.91 f | 146.72 c | 0.60 c | 3.74 f | 8.02 a | 713.30 f |
|  | **1** | 44.87 e | 2.82 d | 19.20 e | 47.01 e | 32.68 e | 16.40 e | 33.64 e | 37.82 e | 157.88 b | 0.59 dc | 3.99 e | 7.07 b | 803.76 e |
|  | **2** | 45.92 d | 2.96 c | 19.82 d | 50.22 d | 35.01 d | 17.76 d | 35.20 d | 39.40 d | 171.22 a | 0.58 d | 4.26 d | 6.18 d | 898.61 d |

**Table S8** (continued).

| **Treatment** | | **SC** | **Chl a** | **Chl b** | **Tot Chl** | **Cart** | **MDA** | **EL** | **CAT** | **SOD** | **GPX** | **APX** | **PPO** |
| --- | --- | --- | --- | --- | --- | --- | --- | --- | --- | --- | --- | --- | --- |
| **Year** | **ML** |  |  |  |  |  |  |  |  |  |  |  |  |
| **1** | **0** | 236.80 c | 8.07 d | 3.22 c | 11.29 d | 5.45 c | 7.65 c | 32.80 c | 0.042 e | 0.058 d | 0.040 d | 0.049 d | 0.069 c |
|  | **1** | 253.06 b | 8.88 b | 3.54 b | 12.43 b | 5.91 b | 7.05 d | 29.62 d | 0.048 c | 0.063 c | 0.044 c | 0.053 b | 0.073 b |
|  | **2** | 269.53 a | 9.69 a | 3.93 a | 13.62 a | 6.59 a | 6.29 e | 25.36 e | 0.055 a | 0.072 a | 0.051 b | 0.060 a | 0.081 a |
| **2** | **0** | 174.01 f | 6.87 f | 2.73 e | 9.60 f | 4.63 e | 9.02 a | 42.71 a | 0.038 f | 0.052 f | 0.045 c | 0.042 f | 0.056 f |
|  | **1** | 187.09 e | 7.62 e | 3.01 d | 10.63 e | 5.16 d | 8.31 b | 38.51 b | 0.043 d | 0.057 e | 0.051 b | 0.045 e | 0.060 e |
|  | **2** | 201.65 d | 8.30 c | 3.34 b | 11.64 c | 5.75 b | 7.43 c | 33.08 c | 0.049 b | 0.065 b | 0.057 a | 0.051 c | 0.065 d |

**Table S8** (continued).

| **Treatment** | | **So pro** | **Prol** | **Flavo** | **Phe** | **Sug** | **H_2_O_2_** | **RWC** | **N** | **Pro** | **Oil** | **SOY** | **SPY** |
| --- | --- | --- | --- | --- | --- | --- | --- | --- | --- | --- | --- | --- | --- |
| **Year** | **ML** |  |  |  |  |  |  |  |  |  |  |  |  |
| **1** | **0** | 58.61 c | 30.82 f | 14.94 f | 24.72 d | 7.47 d | 9.12 c | 62.08 c | 3.17 c | 19.86 c | 6.32 b | 73.26 c | 217.28 b |
|  | **1** | 63.55 b | 35.00 e | 17.56 d | 27.32 c | 9.16 c | 8.47 d | 65.60 b | 3.62 a | 22.64 a | 7.18 a | 90.57 b | 272.68 a |
|  | **2** | 71.44 a | 41.38 c | 21.61 b | 32.35 a | 11.68 b | 7.65 e | 68.13 a | 3.43 b | 21.49 b | 7.00 a | 94.91 a | 277.67 a |
| **2** | **0** | 52.75 e | 37.94 d | 16.94 e | 21.42 e | 9.07 c | 10.60 a | 56.58 e | 1.81 e | 11.32 e | 4.14 d | 30.80 e | 78.51 d |
|  | **1** | 57.20 d | 43.49 b | 20.18 c | 24.96 d | 11.63 b | 9.80 b | 59.11 d | 2.21 d | 13.86 d | 4.65 c | 39.02 d | 109.46 c |
|  | **2** | 64.30 b | 50.99 a | 24.53 a | 28.10 b | 14.60 a | 8.91 c | 61.97 c | 2.15 d | 13.46 d | 4.63 c | 42.76 d | 117.33 c |

**PH:** Plant height, **NMB:** Number of main branches, **NLB:** Number of lateral branches, **NSP:** Number of seeds per plant, **NPP:** Number of pods per plant, **PWP:** Pod weight per plant, **W100S:** 100-seed weight, **W100P:** 100-pod weight, **SSY:** Straw and stubble yield, **HI:** Harvest index, **MBD:** Main branch diameter, **PSP:** Percentage of seedless pods, **GY:** Grain Yield, **SC**: Stomatal Conductance, **Chl a:** Chlorophyll a, **Chl b:** Chlorophyll b, **Tot Chl:** Total chlorophyll, **Cart:** Carotenoid, **MDA**: Malondialdehyde, **EL**: Electrolyte leakage, **CAT:** Catalase, **SOD:** Superoxide dismutase, **GPX:** Guaiacol peroxidase, **APX:** Ascorbate peroxidase, **PPO:** Polyphenol oxidase, **Tot Pro**: Total soluble protein, **Prol**: Proline, **Flavo**: Flavonoid, **Phe**: Phenol, **Sug**: Sugar, **RW**C: Relative water content, **%N**: Seed nitrogen, **%Pro**: Seed protein, **%Oil:** Seed oil, **SOY:** Seed oil yield, **SPY:** Seed protein yield.

Mean comparison was performed by DUNCAN method at 5% probability. Columns with similar letters did not differ significantly

**Table S9.** The mean comparison of interaction effects of different levels of water deficit and 24-epibrassinolide on evaluated traits of chickpea plants

| **Treatment** | | **PH** | **NMB** | **NLB** | **NSP** | **NPP** | **PWP** | **W100S** | **W100P** | **SSY** | **HI** | **MBD** | **PSP** | **GY** |
| --- | --- | --- | --- | --- | --- | --- | --- | --- | --- | --- | --- | --- | --- | --- |
| **Irr** | **EBL** |  |  |  |  |  |  |  |  |  |  |  |  |  |
| **1** | **0** | 48.15 c | 3.10 c | 21.98 c | 60.36 c | 44.01 c | 22.45 c | 38.17 c | 42.55 c | 139.16 c | 0.69 a | 4.46 c | 6.27 c | 1167.75 c |
|  | **1** | 49.34 b | 3.23 b | 22.85 b | 65.29 b | 46.88 b | 23.97 b | 39.61 b | 44.02 b | 150.16 b | 0.68 a | 4.83 b | 5.63 d | 1309.32 b |
|  | **2** | 50.69 a | 3.50 a | 23.46 a | 69.07 a | 48.64 a | 25.43 a | 41.48 a | 45.96 a | 164.94 a | 0.67 b | 5.13 a | 4.97 e | 1454.65 a |
| **2** | **0** | 43.80 e | 2.50 f | 19.06 f | 39.37 f | 28.14 f | 13.52 f | 31.23 f | 36.85 f | 112.77 f | 0.65 c | 3.60 f | 8.50 a | 619.02 f |
|  | **1** | 45.77 d | 2.63 e | 20.60 e | 44.36 e | 30.94 e | 14.90 e | 32.48 e | 38.73 e | 123.55 e | 0.64 dc | 3.82 e | 7.21 b | 727.50 e |
|  | **2** | 46.27 d | 2.83 d | 21.45 d | 46.57 d | 34.03 d | 16.02 d | 34.15 d | 40.18 d | 133.44 d | 0.64 d | 4.16 d | 6.04 c | 800.36 d |

**Table S9** (continued).

| **Treatment** | | **SC** | **Chl a** | **Chl b** | **Tot Chl** | **Cart** | **MDA** | **EL** | **CAT** | **SOD** | **GPX** | **APX** | **PPO** |
| --- | --- | --- | --- | --- | --- | --- | --- | --- | --- | --- | --- | --- | --- |
| **Irr** | **EBL** |  |  |  |  |  |  |  |  |  |  |  |  |
| **1** | **0** | 247.97 c | 9.47 c | 3.88 c | 13.35 c | 6.53 c | 7.41 c | 32.78 c | 0.034 f | 0.044 f | 0.036 f | 0.038 f | 0.054 f |
|  | **1** | 263.25 b | 10.55 b | 4.61 b | 15.16 b | 7.00 b | 7.14 dc | 31.99 d | 0.039 e | 0.047 e | 0.041 e | 0.043 e | 0.058 e |
|  | **2** | 278.16 a | 11.55 a | 5.05 a | 16.61 a | 7.48 a | 6.96 de | 31.16 e | 0.045 d | 0.054 d | 0.047 d | 0.049 d | 0.065 d |
| **2** | **0** | 159.73 f | 5.33 f | 1.85 f | 7.18 f | 3.63 f | 9.52 a | 44.06 a | 0.046 c | 0.066 c | 0.049 c | 0.052 c | 0.070 c |
|  | **1** | 175.83 e | 5.90 e | 2.06 e | 7.97 e | 4.14 e | 8.00 b | 34.85 b | 0.052 b | 0.073 b | 0.053 b | 0.056 b | 0.075 b |
|  | **2** | 197.20 d | 6.63 d | 2.32 d | 8.95 d | 4.70 d | 6.72 e | 27.24 f | 0.059 a | 0.085 a | 0.061 a | 0.063 a | 0.082 a |

**Table S9** (continued).

| **Treatment** | | **So pro** | **Prol** | **Flavo** | **Phe** | **Sug** | **H_2_O_2_** | **RWC** | **N** | **Pro** | **Oil** | **SOY** | **SPY** |
| --- | --- | --- | --- | --- | --- | --- | --- | --- | --- | --- | --- | --- | --- |
| **Irr** | **EBL** |  |  |  |  |  |  |  |  |  |  |  |  |
| **1** | **0** | 48.76 e | 27.19 f | 12.64 e | 17.46 f | 6.58 f | 7.52 d | 73.70 c | 2.24 e | 14.02 e | 5.80 b | 71.06 c | 176.70 c |
|  | **1** | 55.52 d | 31.61 e | 14.44 d | 19.84 e | 8.23 e | 6.86 e | 76.47 b | 2.64 dc | 16.52 dc | 6.46 a | 88.67 b | 231.36 b |
|  | **2** | 65.33 b | 38.15 d | 18.98 c | 24.34 d | 10.47 c | 6.26 f | 78.54 a | 2.58 d | 16.15 d | 6.39 a | 96.96 a | 247.51 a |
| **2** | **0** | 61.11 c | 41.77 c | 19.05 c | 28.04 c | 9.93 d | 12.47 a | 44.40 f | 2.71 c | 16.94 c | 4.59 d | 29.69 e | 109.92 e |
|  | **1** | 65.33 b | 45.39 b | 22.72 b | 31.28 b | 12.19 b | 11.33 b | 48.52 e | 3.19 a | 19.93 a | 5.35 c | 41.20 d | 152.21 d |
|  | **2** | 71.77 a | 55.51 a | 27.92 a | 37.92 a | 16.23 a | 10.11 c | 51.86 d | 3.05 b | 19.06 b | 5.32 c | 43.72 d | 155.23 d |

**PH:** Plant height, **NMB:** Number of main branches, **NLB:** Number of lateral branches, **NSP:** Number of seeds per plant, **NPP:** Number of pods per plant, **PWP:** Pod weight per plant, **W100S:** 100-seed weight, **W100P:** 100-pod weight, **SSY:** Straw and stubble yield, **HI:** Harvest index, **MBD:** Main branch diameter, **PSP:** Percentage of seedless pods, **GY:** Grain Yield, **SC**: Stomatal Conductance, **Chl a:** Chlorophyll a, **Chl b:** Chlorophyll b, **Tot Chl:** Total chlorophyll, **Cart:** Carotenoid, **MDA**: Malondialdehyde, **EL**: Electrolyte leakage, **CAT:** Catalase, **SOD:** Superoxide dismutase, **GPX:** Guaiacol peroxidase, **APX:** Ascorbate peroxidase, **PPO:** Polyphenol oxidase, **Tot Pro**: Total soluble protein, **Prol**: Proline, **Flavo**: Flavonoid, **Phe**: Phenol, **Sug**: Sugar, **RW**C: Relative water content, **%N**: Seed nitrogen, **%Pro**: Seed protein, **%Oil:** Seed oil, **SOY:** Seed oil yield, **SPY:** Seed protein yield.

Mean comparison was performed by DUNCAN method at 5% probability. Columns with similar letters did not differ significantly

**Table 10.** The mean comparison of interaction effects of different levels of water deficit and melatonin on evaluated traits of chickpea plants

| **Treatment** | | **PH** | **NMB** | **NLB** | **NSP** | **NPP** | **PWP** | **W100S** | **W100P** | **SSY** | **HI** | **MBD** | **SPS** | **GY** |
| --- | --- | --- | --- | --- | --- | --- | --- | --- | --- | --- | --- | --- | --- | --- |
| **Irr** | **ML** |  |  |  |  |  |  |  |  |  |  |  |  |  |
| **1** | **0** | 47.98 c | 3.14 c | 21.96 c | 61.84 c | 44.37 c | 22.66 c | 38.35 c | 42.55 c | 139.55 c | 0.69 a | 4.49 c | 6.27 c | 1203.90 c |
|  | **1** | 49.37 b | 3.25 b | 22.77 b | 65.14 b | 46.49 b | 23.87 b | 39.52 b | 44.13 b | 151.44 b | 0.68 ba | 4.81 b | 5.60 d | 1421.29 b |
|  | **2** | 50.83 a | 3.43 a | 23.56 a | 67.74 a | 48.67 a | 25.32 a | 41.40 a | 45.85 a | 163.27 a | 0.68 b | 5.11 a | 5.00 e | 1421.29 a |
| **2** | **0** | 43.94 f | 2.50 f | 19.56 f | 39.66 f | 28.27 f | 13.64 f | 31.40 f | 36.97 f | 115.27 f | 0.65 c | 3.61 f | 8.33 a | 627.19 f |
|  | **1** | 45.45 e | 2.67 e | 20.40 e | 43.53 e | 31.00 e | 14.80 e | 32.55 e | 38.67 e | 121.88 e | 0.65 c | 3.85 e | 7.22 b | 714.23 e |
|  | **2** | 46.45 d | 2.78 d | 21.15 d | 47.12 d | 33.74 d | 15.99 d | 33.91 d | 40.12 d | 132.61 d | 0.63 d | 4.13 d | 6.20 c | 805.47 d |

**Table 10** (continued).

| **Treatment** | | **SC** | **Chl a** | **Chl b** | **Tot Chl** | **Cart** | **MDA** | **EL** | **CAT** | **SOD** | **GPX** | **APX** | **PPO** |
| --- | --- | --- | --- | --- | --- | --- | --- | --- | --- | --- | --- | --- | --- |
| **Irr** | **ML** |  |  |  |  |  |  |  |  |  |  |  |  |
| **1** | **0** | 249.60 c | 9.60 c | 4.05 c | 13.66 c | 6.48 c | 7.41 c | 32.66 c | 0.034 f | 0.044 f | 0.036 f | 0.039 f | 0.054 f |
|  | **1** | 262.17 b | 10.52 b | 4.51 b | 15.03 b | 6.94 b | 7.13 dc | 32.03 c | 0.039 e | 0.048 e | 0.041 e | 0.043 e | 0.058 e |
|  | **2** | 277.62 a | 11.45 a | 4.98 a | 16.43 a | 7.59 a | 6.98 de | 31.24 d | 0.045 d | 0.053 d | 0.047 d | 0.049 d | 0.064 d |
| **2** | **0** | 161.21 f | 5.33 f | 1.90 e | 7.23 f | 3.59 f | 9.26 a | 42.85 a | 0.047 c | 0.067 c | 0.048 c | 0.052 c | 0.071 c |
|  | **1** | 177.99 e | 5.98 e | 2.04 e | 8.03 e | 4.13 e | 8.23 b | 36.11 b | 0.052 b | 0.073 b | 0.054 b | 0.056 b | 0.075 b |
|  | **2** | 193.56 d | 6.54 d | 2.29 d | 8.84 d | 4.75 d | 6.74 e | 27.20 e | 0.058 a | 0.084 a | 0.061 a | 0.063 a | 0.082 a |

**Table 10** (continued).

| **Treatment** | | **So pro** | **Prol** | **Flavo** | **Phe** | **Sug** | **H_2_O_2_** | **RWC** | **N** | **Pro** | **Oil** | **SOY** | **SPY** |
| --- | --- | --- | --- | --- | --- | --- | --- | --- | --- | --- | --- | --- | --- |
| **Irr** | **ML** |  |  |  |  |  |  |  |  |  |  |  |  |
| **1** | **0** | 49.71 f | 27.16 f | 12.59 f | 17.91 f | 6.68 f | 7.39 d | 73.88 c | 2.24 e | 14.01 e | 5.79 b | 73.40 c | 183.08 b |
|  | **1** | 55.41 e | 31.56 e | 15.14 e | 20.14 e | 8.26 e | 6.86 e | 76.35 b | 2.64 dc | 16.52 dc | 6.49 a | 89.46 b | 232.95 a |
|  | **2** | 64.49 c | 38.24 d | 18.33 d | 23.60 d | 10.35 c | 6.40 f | 78.47 a | 2.58 d | 16.16 d | 6.37 a | 93.83 a | 239.54 a |
| **2** | **0** | 61.64 d | 41.60 c | 19.28 c | 28.24 c | 9.86 d | 12.33 a | 44.78 f | 2.74 c | 17.17 c | 4.68 d | 30.6 e | 112.72 d |
|  | **1** | 65.33 b | 46.93 b | 22.60 b | 32.14 b | 12.54 b | 11.41 b | 48.37 e | 3.19 a | 19.98 a | 5.34 c | 40.13 d | 149.18 c |
|  | **2** | 71.25 a | 54.14 a | 27.80 a | 36.86 a | 15.94 a | 10.16 c | 51.63 d | 3.00 b | 18.79 b | 5.25 c | 43.83 d | 155.45 c |

**PH:** Plant height, **NMB:** Number of main branches, **NLB:** Number of lateral branches, **NSP:** Number of seeds per plant, **NPP:** Number of pods per plant, **PWP:** Pod weight per plant, **W100S:** 100-seed weight, **W100P:** 100-pod weight, **SSY:** Straw and stubble yield, **HI:** Harvest index, **MBD:** Main branch diameter, **PSP:** Percentage of seedless pods, **GY:** Grain Yield, **SC**: Stomatal Conductance, **Chl a:** Chlorophyll a, **Chl b:** Chlorophyll b, **Tot Chl:** Total chlorophyll, **Cart:** Carotenoid, **MDA**: Malondialdehyde, **EL**: Electrolyte leakage, **CAT:** Catalase, **SOD:** Superoxide dismutase, **GPX:** Guaiacol peroxidase, **APX:** Ascorbate peroxidase, **PPO:** Polyphenol oxidase, **Tot Pro**: Total soluble protein, **Prol**: Proline, **Flavo**: Flavonoid, **Phe**: Phenol, **Sug**: Sugar, **RW**C: Relative water content, **%N**: Seed nitrogen, **%Pro**: Seed protein, **%Oil:** Seed oil, **SOY:** Seed oil yield, **SPY:** Seed protein yield.

Mean comparison was performed by DUNCAN method at 5% probability. Columns with similar letters did not differ significantly

| **Treatment** | | **PH** | **NMB** | **NLB** | **NSP** | **NPP** | **PWP** | **W100S** | **W100P** | **SSY** | **HI** | **MBD** | **PSP** | **GY** |
| --- | --- | --- | --- | --- | --- | --- | --- | --- | --- | --- | --- | --- | --- | --- |
| **EBL** | **ML** |  |  |  |  |  |  |  |  |  |  |  |  |  |
| **0** | **0** | 44.80 f | 2.71 f | 19.68 f | 48.10 g | 34.30 e | 16.98 e | 33.99 d | 38.55 e | 117.50 e | 0.67 a | 3.79 e | 8.268 a | 846.05 f |
|  | **1** | 45.96 e | 2.80 e | 20.58 e | 49.27 gf | 36.04 d | 17.90 d | 34.35 d | 39.69 d | 124.83 d | 0.67 ba | 4.00 d | 7.40 b | 872.90 fe |
|  | **2** | 47.16 dc | 2.88 d | 21.31 dc | 52.22 ed | 37.89 c | 19.07 c | 35.76 c | 40.87 c | 135.58 c | 0.66 bc | 4.29 c | 6.49 c | 961.21 d |
| **1** | **0** | 46.08 e | 2.80 e | 21.04 de | 50.79 ef | 36.56 d | 18.27 d | 34.46 d | 39.80 d | 127.25 d | 0.67 ba | 4.04 d | 7.30 b | 906.06 e |
|  | **1** | 47.76 c | 2.95 d | 21.66 c | 55.53 c | 38.99 c | 19.35 c | 35.99 c | 41.45 c | 136.41 c | 0.66 bc | 4.33 c | 6.39 c | 1026.81 c |
|  | **2** | 48.83 b | 3.05 c | 22.48 b | 58.16 b | 41.18 b | 20.66 b | 37.68 b | 42.88 b | 146.91 b | 0.66 c | 4.60 b | 5.56 d | 1122.37 b |
| **2** | **0** | 47.00 d | 2.95 d | 21.56 c | 53.38 d | 38.11 c | 19.19 c | 36.18 c | 40.94 c | 137.50 c | 0.66 bc | 4.31 c | 6.34 c | 994.53 dc |
|  | **1** | 48.51 b | 3.15 b | 22.52 b | 58.20 b | 41.35 b | 20.76 b | 37.76 b | 43.05 b | 148.75 b | 0.67 ba | 4.66 b | 5.43 d | 1131.42 b |
|  | **2** | 49.93 a | 3.39 a | 23.27 a | 61.90 a | 44.55 a | 22.23 a | 39.52 a | 45.22 a | 161.33 a | 0.64 d | 4.96 a | 4.74 e | 1256.56 a |

**Table S11.** The mean comparison of interaction effects of different melatonin levels and 24-epibrassinolide on evaluated traits of chickpea plants

**Table 11** (continued).

| **Treatment** | | **SC** | **Chl a** | **Chl b** | **Tot Chl** | **Cart** | **MDA** | **EL** | **CAT** | **SOD** | **GPX** | **APX** | **PPO** |
| --- | --- | --- | --- | --- | --- | --- | --- | --- | --- | --- | --- | --- | --- |
| **EBL** | **ML** |  |  |  |  |  |  |  |  |  |  |  |  |
| **0** | **0** | 191.65 e | 6.72 e | 2.63 e | 9.36 e | 4.62 e | 9.49 a | 43.23 a | 0.03 e | 0.04 g | 0.03 f | 0.04 g | 0.05 h |
|  | **1** | 203.68 d | 7.44 d | 2.82 e | 10.26 d | 5.09 d | 8.52 b b | 39.10 b | 0.0404 d | 0.05 f | 0.04 e | 0.04 f | 0.06 g |
|  | **2** | 216.21 c | 8.03 c | 3.14 dc | 11.18 c | 5.530 c | 7.38 c | 32.94 de | 0.046 c | 0.06 d | 0.04 c | 0.05 d | 0.06 e |
| **1** | **0** | 204.12 d | 7.49 d | 3.05 d | 10.54 d | 5.04 c | 8.19 b | 37.54 c | 0.0403 d | 0.05 f | 0.04 e | 0.04 f | 0.06 g |
|  | **1** | 218.87 c | 8.09 c | 3.30 c | 11.39 | 5.532 c | 7.62 c | 33.81 d | 0.0452 c | 0.58 e | 0.04 d | 0.04 e | 0.06 f |
|  | **2** | 235.64 b | 9.10 b | 3.66 b | 12.77 b | 6.16 b | 6.90 d | 28.92 f | 0.05 b | 0.06 c | 0.05 b | 0.05 b | 0.07 c |
| **2** | **0** | 220.44 c | 8.19 c | 3.25 dc | 11.44 c | 5.46 c | 7.32 c | 32.50 e | 0.0459 c | 0.06 d | 0.04 c | 0.05 c | 0.06 d |
|  | **1** | 237.68 b | 9.23 b | 3.71 b | 12.94 b | 5.99 b | 6.90 d | 29.30 f | 0.0525 b | 0.06 b | 0.05 b | 0.05 b | 0.07 b |
|  | **2** | 254.93 a | 9.86 a | 4.10 a | 13.96 a | 6.82 a | 6.30 e | 25.79 g | 0.0590 a | 0.07 a | 0.06 a | 0.06 a | 0.08 a |

**Table 11** (continued).

| **Treatment** | | **So pro** | **Prol** | **Flavo** | **Phe** | **Sug** | **H_2_O_2_** | **RWC** | **N** | **Pro** | **Oil** | **SOY** | **SPY** |
| --- | --- | --- | --- | --- | --- | --- | --- | --- | --- | --- | --- | --- | --- |
| **EBL** | **ML** |  |  |  |  |  |  |  |  |  |  |  |  |
| **0** | **0** | 48.29 h | 28.63 h | 12.60 g | 20.02 g | 6.21 g | 10.73 a | 57.08 f | 2.16 e | 13.53 e | 4.70 d | 43.00 f | 117.80 e |
|  | **1** | 54.30 g | 33.98 f | 15.77 f | 22.54 f | 8.22 f | 10.05 b | 58.86 e | 2.44 d | 15.26 d | 5.19 c | 48.98 e | 136.58 d |
|  | **2** | 62.22 d | 40.83 d | 19.17 d | 25.69 e | 10.33 d | 9.21 c | 61.21 d | 2.82 c | 17.65 c | 5.71 b | 59.15 d | 175.56 c |
| **1** | **0** | 55.89 f | 33.19 g | 15.75 f | 22.37 f | 8.16 f | 9.84 b | 59.39 e | 2.45 d | 15.34 d | 5.24 c | 51.35 e | 141.30 d |
|  | **1** | 58.74 e | 38.10 e | 17.83 e | 25.35 e | 9.76 e | 9.14 c | 62.86 c | 2.97 b | 18.59 b | 5.97 b | 65.85 cb | 196.66 b |
|  | **2** | 66.65 c | 44.21 c | 22.18 c | 28.96 c | 12.70 c | 8.32 d | 65.23 b | 3.31 a | 20.74 a | 6.50 a | 77.62 a | 237.39 a |
| **2** | **0** | 62.85 d | 41.32 d | 19.50 d | 26.84 d | 10.44 d | 9.02 c | 61.53 d | 2.86 cb | 17.89 cb | 5.76 b | 61.73 cd | 184.59 c |
|  | **1** | 68.08 b | 45.64 b | 23.00 b | 30.52 b | 13.21 b | 8.23 d | 65.35 b | 3.34 a | 20.89 a | 6.59 a | 79.56 a | 239.97 a |
|  | **2** | 74.73 a | 53.53 a | 27.85 a | 36.03 a | 16.40 a | 7.30 e | 68.72 a | 2.24 e | 14.04 e | 5.22 c | 69.73 b | 179.55 c |

**PH:** Plant height, **NMB:** Number of main branches, **NLB:** Number of lateral branches, **NSP:** Number of seeds per plant, **NPP:** Number of pods per plant, **PWP:** Pod weight per plant, **W100S:** 100-seed weight, **W100P:** 100-pod weight, **SSY:** Straw and stubble yield, **HI:** Harvest index, **MBD:** Main branch diameter, **PSP:** Percentage of seedless pods, **GY:** Grain Yield, **SC**: Stomatal Conductance, **Chl a:** Chlorophyll a, **Chl b:** Chlorophyll b, **Tot Chl:** Total chlorophyll, **Cart:** Carotenoid, **MDA**: Malondialdehyde, **EL**: Electrolyte leakage, **CAT:** Catalase, **SOD:** Superoxide dismutase, **GPX:** Guaiacol peroxidase, **APX:** Ascorbate peroxidase, **PPO:** Polyphenol oxidase, **Tot Pro**: Total soluble protein, **Prol**: Proline, **Flavo**: Flavonoid, **Phe**: Phenol, **Sug**: Sugar, **RW**C: Relative water content, **%N**: Seed nitrogen, **%Pro**: Seed protein, **%Oil:** Seed oil, **SOY:** Seed oil yield, **SPY:** Seed protein yield. Mean comparison was performed by DUNCAN method at 5% probability. Columns with similar letters did not differ significantly

| **Traetment** | | | **PH** | **NMB** | NLB | **NSP** | NPP | **PWP** | **W100S** | **W100P** | **SSY** | **HI** | **MBD** | **PSP** | **GY** |
| --- | --- | --- | --- | --- | --- | --- | --- | --- | --- | --- | --- | --- | --- | --- | --- |
| **Year** | **Irr** | **EBL** |  |  |  |  |  |  |  |  |  |  |  |  |  |
| **1** | **1** | **0** | 51.13 c | 3.13 c | 24.31 c | 70.17 c | 54.55 b | 25.52 c | 41.18 c | 46.59 c | 116.88 g | 0.76 a | 4.72 c | 5.53 e | 1445.74 c |
|  |  | **1** | 52.15 b | 3.35 b | 25.06 b | 74.50 b | 57.51 a | 27.30 b | 42.88 b | 47.65 b | 124.22 f | 0.76 a | 5.13 b | 4.91 f | 1597.92 b |
|  |  | **2** | 52.15 a | 3.63 a | 25.74 a | 79.80 a | 58.40 a | 28.70 a | 44.99 a | 49.21 a | 140.66 e | 0.74 b | 5.47 a | 4.43 g | 1797.29 a |
|  | **2** | **0** | 45.28 f | 2.64 e | 20.86 e | 43.66 g | 29.71 g | 16.09 h | 32.91 h | 40.34 f | 97.66 j | 0.72 c | 3.95 f | 7.82 b | 719.06 h |
|  |  | **1** | 48.02 d | 2.83 d | 23.22 d | 49.80 f | 32.80 fe | 17.37 g | 34.05 g | 42.44 e | 105.22 i | 0.71 c | 4.23 e | 6.53 d | 851.17 g |
|  |  | **2** | 48.40 d | 3.05 c | 24.48 cb | 51.46 f | 36.40 d | 18.93 f | 35.48 f | 44.09 d | 111.77 h | 0.73 c | 4.51 d | 5.47 e | 914.70 f |
| **2** | **1** | **0** | 45.17 f | 3.06 c | 19.66 f | 50.55 f | 33.47 e | 19.38 f | 35.16 f | 38.51 g | 161.44 c | 0.62 d | 4.19 c | 7.01 c | 889.76 gf |
|  |  | **1** | 46.53 e | 3.12 c | 20.64 e | 56.08 e | 36.26 d | 20.64 e | 36.33 e | 40.40 f | 176.11 b | 0.61 d | 4.53 d | 6.34 d | 1020.73 e |
|  |  | **2** | 47.56 d | 3.36 b | 21.17 e | 58.35 d | 38.89 c | 22.17 d | 37.98 d | 42.70 e | 189.22 a | 0.61 d | 4.78 c | 5.52 e | 686.03 h |
|  |  | **0** | 42.31 h | 2.35 f | 17.26 h | 35.08 j | 26.57 h | 10.94 j | 29.55 j | 33.36 j | 127.88 f | 0.58 e | 3.25 i | 9.18 a | 518.99 j |
|  | **2** | **1** | 43.53 g | 2.43 f | 17.98 g | 38.93 i | 29.08 g | 12.43 i | 30.91 i | 35.02 i | 141.88 e | 0.56 f | 3.42 h | 7.88 b | 603.84 i |
|  |  | **2** | 44.15 g | 2.62 e | 18.41 g | 41.68 h | 31.66 f | 13.11 i | 32.83 h | 36.27 h | 155.11 d | 0.56 f | 3.81 g | 6.61 d | 686.03 h |

**Table S12.** The mean comparison of interaction effects of **d**ifferent levels of water deficit, year and 24-epibrassinolide on evaluated traits of chickpea plants

**Table 12** (continued).

| **Traetment** | | | **SC** | **Chl a** | **Chl b** | **Tot Chl** | **Cart** | **MDA** | **EL** | **CAT** | **SOD** | **GPX** | **APX** | **PPO** |
| --- | --- | --- | --- | --- | --- | --- | --- | --- | --- | --- | --- | --- | --- | --- |
| **Year** | **Irr** | **EBL** |  |  |  |  |  |  |  |  |  |  |  |  |
| **1** | **1** | **0** | 287.45 c | 10.20 d | 4.20 d | 14.04 d | 6.97 c | 6.90 gf | 28.44 g | 0.03 g | 0.04 i | 0.03 h | 0.04 j | 0.05 i |
|  |  | **1** | 307.19 b | 11.39 b | 4.98 b | 16.38 b | 7.53 b | 6.60 gh | 27.82 gh | 0.04 f | 0.05 h | 0.03 h | 0.04 h | 0.06 g |
|  |  | **2** | 325.88 a | 12.50 a | 5.46 a | 17.96 a | 8.07 a | 6.43 hi | 27.10 h | 0.04 d | 0.05 f | 0.04 gf | 0.05 e | 0.07 e |
|  | **2** | **0** | 179.79 h | 5.76 i | 2.00 h | 7.76 i | 3.85 h | 8.65 b | 38.22 c | 0.04 c | 0.07 d | 0.04 f | 0.05 d | 0.07 c |
|  |  | **1** | 197.62 g | 6.31 h | 2.23 g | 8.55 h | 4.42 g | 7.27 ef | 30.31 f | 0.05 b | 0.07 c | 0.05 e | 0.06 b | 0.08 b |
|  |  | **2** | 220.86 e | 7.12 g | 2.51 f | 9.63 g | 5.06 f | 6.11 i | 23.68 i | 0.06 a | 0.08 a | 0.05 b | 0.06 a | 0.09 a |
| **2** | **1** | **0** | 208.48 f | 8.73 f | 3.57 e | 12.30 f | 6.09 e | 7.93 c | 37.12 d | 0.03 h | 0.04 k | 0.03 h | 0.03 l | 0.04 l |
|  |  | **1** | 219.31 e | 9.71 e | 4.24 d | 13.95 e | 6.48 d | 7.69 cd | 36.16 de | 0.03 g | 0.04 j | 0.04 fg | 0.04 k | 0.05 k |
|  |  | **2** | 230.45 d | 10.61 c | 4.64 c | 15.25 c | 6.89 c | 7.49 de | 35.23 e | 0.04 e | 0.05 g | 0.05 e | 0.04 i | 0.05 j |
|  |  | **0** | 139.66 k | 4.89 j | 1.70 i | 6.59 k | 3.41 i | 10.38 a | 49.91 a | 0.04 e | 0.06 e | 0.05 d | 0.04 h | 0.06 h |
|  | **2** | **1** | 154.05 j | 5.49 i | 1.90 hi | 7.400 j | 3.87 h | 8.72 b | 39.40 b | 0.04 cd | 0.06 d | 0.05 c | 0.05 f | 0.06 f |
|  |  | **2** | 173.54 i | 6.13 h | 2.13 gh | 8.27 h | 4.33 g | 7.33 de | 30.79 f | 0.05 b | 0.08 b | 0.06 a | 0.05 c | 0.07 d |

**Table 12** (continued).

| **Traetment** | | | **So pro** | **Prol** | **Flavo** | **Phe** | **Sug** | **H_2_O_2_** | **RWC** | **N** | **Pro** | **Oil** | **SOY** | **SPY** |
| --- | --- | --- | --- | --- | --- | --- | --- | --- | --- | --- | --- | --- | --- | --- |
| **Year** | **Irr** | **EBL** |  |  |  |  |  |  |  |  |  |  |  |  |
| **1** | **1** | **0** | 51.33 g | 24.20 j | 11.66 i | 18.79 h | 5.85 i | 6.94 gh | 77.84 c | 2.93 e | 18.33 e | 6.88 b | 99.98 c | 266.31 c |
|  |  | **1** | 58.44 e | 28.22 i | 13.72 h | 20.90 g | 7.23 h | 6.35 ij | 80.02 b | 3.38 dc | 21.14 dc | 7.67 a | 123.31 b | 339.60 b |
|  |  | **2** | 68.77 b | 34.22 g | 17.40 f | 25.90 e | 9.35 f | 5.93 j | 81.39 a | 3.24 d | 20.29 d | 7.61 a | 136.39 a | 361.76 a |
|  | **2** | **0** | 64.33 c | 37.33 f | 17.88 f | 29.77 d | 8.76 g | 11.55 c | 45.93 j | 3.41 c | 21.34 c | 5.65 d | 40.91 e | 154.13 e |
|  |  | **1** | 68.77 b | 40.66 e | 21.33 d | 33.22 c | 10.88 e | 10.40 d | 51.42 h | 3.85 a | 24.09 a | 6.73 cb | 58.06 d | 207.52 d |
|  |  | **2** | 75.55 a | 49.77 b | 26.22 b | 40.22 a | 14.55 b | 9.33 e | 55.03 g | 3.64 b | 22.77 b | 6.46 c | 58.83 d | 205.93 d |
| **2** | **1** | **0** | 42.20 h | 30.19 h | 13.62 h | 16.13 i | 7.32 h | 8.11 f | 69.56 f | 1.55 h | 9.70 h | 4.72 f | 42.14 e | 87.08 h |
|  |  | **1** | 52.60 f | 35.00 g | 15.17 g | 18.79 h | 9.23 f | 7.37 g | 72.91 e | 1.90 g | 11.89 g | 5.25 e | 54.03 d | 123.12 f |
|  |  | **2** | 61.90 d | 42.08 d | 20.56 e | 22.79 f | 11.60 d | 6.60 ih | 75.68 d | 1.92 g | 12.00 g | 5.18 e | 57.52 d | 133.26 f |
|  |  | **0** | 57.90 e | 46.21 c | 20.21 e | 26.31 e | 11.09 e | 13.38 a | 42.87 k | 2.00 g | 12.54 g | 3.54 h | 18.48 g | 65.72 i |
|  | **2** | **1** | 61.90 d | 50.12 b | 24.10 c | 29.34 d | 13.49 c | 12.27 b | 45.62 j | 2.52 f | 15.78 f | 3.98 g | 24.35 gf | 96.89 hg |
|  |  | **2** | 68.00 b | 61.24 a | 29.63 a | 35.61 b | 17.90 a | 10.88 d | 48.68 i | 2.45 f | 15.36 f | 4.18 g | 28.62 f | 104.52 g |

**PH:** Plant height, **NMB:** Number of main branches, **NLB:** Number of lateral branches, **NSP:** Number of seeds per plant, **NPP:** Number of pods per plant, **PWP:** Pod weight per plant, **W100S:** 100-seed weight, **W100P:** 100-pod weight, **SSY:** Straw and stubble yield, **HI:** Harvest index, **MBD:** Main branch diameter, **PSP:** Percentage of seedless pods, **GY:** Grain Yield, **SC**: Stomatal Conductance, **Chl a:** Chlorophyll a, **Chl b:** Chlorophyll b, **Tot Chl:** Total chlorophyll, **Cart:** Carotenoid, **MDA**: Malondialdehyde, **EL**: Electrolyte leakage, **CAT:** Catalase, **SOD:** Superoxide dismutase, **GPX:** Guaiacol peroxidase, **APX:** Ascorbate peroxidase, **PPO:** Polyphenol oxidase, **Tot Pro**: Total soluble protein, **Prol**: Proline, **Flavo**: Flavonoid, **Phe**: Phenol, **Sug**: Sugar, **RW**C: Relative water content, **%N**: Seed nitrogen, **%Pro**: Seed protein, **%Oil:** Seed oil, **SOY:** Seed oil yield, **SPY:** Seed protein yield.

Mean comparison was performed by DUNCAN method at 5% probability. Columns with similar letters did not differ significantly

**Table S13.** The mean comparison of interaction effects of **d**ifferent levels of water deficit, year and melatonin on evaluated traits of chickpea plants

| **Traetment** | | | **PH** | **NMB** | NLB | **NSP** | NPP | **PWP** | **W100S** | **W100P** | **SSY** | **HI** | **MBD** | **PSP** | **GY** |
| --- | --- | --- | --- | --- | --- | --- | --- | --- | --- | --- | --- | --- | --- | --- | --- |
| **Year** | **Irr** | **ML** |  |  |  |  |  |  |  |  |  |  |  |  |  |
| **1** | **1** | **0** | 50.53 c | 3.24 cd | 24.14 c | 72.83 b | 54.77 c | 25.85 c | 41.44 c | 46.56 c | 117.11 g | 0.76 a | 4.79 c | 5.51 f | 1509.87 c |
|  |  | **1** | 52.46 b | 3.33 b | 25.05 b | 75.15 a | 56.93 b | 27.15 b | 42.74 b | 47.66 b | 127.33 f | 0.75 ba | 5.10 b | 4.91 g | 1610.14 b |
|  |  | **2** | 54.11 a | 3.54 a | 25.92 a | 76.48 a | 58.75 a | 28.52 a | 44.87 a | 49.23 a | 137.33 e | 0.74 bc | 5.43 a | 4.45 h | 1720.94 a |
|  | **2** | **0** | 45.64 gf | 2.67 g | 21.80 e | 43.96 h | 29.95 h | 16.41 i | 32.99 h | 40.66 f | 99.11 i | 0.72 d | 3.93 f | 7.65 b | 725.73 h |
|  |  | **1** | 47.44 e | 2.88 f | 22.91 d | 48.17 g | 32.88 f | 17.41 h | 34.11 g | 42.28 e | 103.55 i | 0.74 c | 4.23 e | 6.58 de | 823.83 g |
|  |  | **2** | 48.62 d | 2.97 e | 23.86 c | 52.79 e | 36.06 e | 18.57 g | 35.34 f | 43.93 d | 112.00 h | 0.70 e | 4.53 d | 5.59 f | 935.37 f |
| **2** | **1** | **0** | 45.43 g | 3.04 e | 19.77 g | 50.86 f | 33.96 f | 19.47 f | 35.26 f | 38.54 g | 162.00 c | 0.62 f | 4.19 e | 7.03 c | 897.94 gf |
|  |  | **1** | 46.28 f | 3.17 d | 20.50 f | 55.13 d | 36.06 e | 20.60 e | 36.29 e | 40.60 f | 175.55 b | 0.61 gf | 4.52 d | 6.29 e | 1002.90 e |
|  |  | **2** | 47.55 e | 3.33 cb | 21.21 e | 59.00 c | 38.60 d | 22.12 d | 37.92 d | 42.48 e | 189.22 a | 0.61 g | 4.79 c | 5.55 f | 1121.65 d |
|  |  | **0** | 42.24 i | 2.33 i | 17.33 i | 35.37 k | 26.60 i | 10.87 l | 29.81 j | 33.29 j | 131.44 f | 0.57 h | 3.28 i | 9.01 a | 528.66 k |
|  | **2** | **1** | 43.46 h | 2.47 h | 17.90 ih | 38.88 j | 29.31 h | 12.20 k | 30.99 i | 35.05 i | 140.22 e | 0.57 ih | 3.47 h | 7.85 b | 604.62 j |
|  |  | **2** | 44.28 h | 2.60 g | 18.43 h | 41.44 i | 31.42 g | 13.41 j | 32.48 h | 36.32 h | 153.22 d | 0.56 i | 3.72 g | 6.81 dc | 675.57 i |

**Table 13** (continued).

| **Traetment** | | | **SC** | **Chl a** | **Chl b** | **Tot Chl** | **Cart** | **MDA** | **EL** | **CAT** | **SOD** | **GPX** | **APX** | **PPO** |
| --- | --- | --- | --- | --- | --- | --- | --- | --- | --- | --- | --- | --- | --- | --- |
| **Year** | **Irr** | **ML** |  |  |  |  |  |  |  |  |  |  |  |  |
| **1** | **1** | **0** | 292.57 c | 10.39 c | 4.38 c | 14.78 c | 7.01 c | 6.87 f | 28.34 f | 0.03 i | 0.04 h | 0.03 h | 0.04 j | 0.06 h |
|  |  | **1** | 305.70 b | 11.34 b | 4.87 b | 16.22 b | 7.43 b | 6.61 gf | 27.85 gf | 0.04 g | 0.05g | 0.03 g | 0.04 h | 0.06 g |
|  |  | **2** | 322.24 a | 12.36 a | 5.38 a | 17.75 a | 8.12 a | 6.45 gh | 27.17 g | 0.04 d | 0.05 f | 0.04 f | 0.05 e | 0.07 e |
|  | **2** | **0** | 181.02 h | 5.75 i | 2.05 gh | 7.80 i | 3.88 h | 8.42 c | 37.26 c | 0.04 c | 0.07 d | 0.04 e | 0.05 d | 0.07 c |
|  |  | **1** | 200.42 g | 6.43 g | 2.21 g | 8.64 g | 4.39 g | 7.48 e | 31.40 e | 0.05 b | 0.07d | 0.05 d | 0.06 b | 0.08 b |
|  |  | **2** | 216.82 e | 7.02 f | 2.47 f | 9.50 f | 5.05 f | 6.13 h | 23.55 h | 0.06 a | 0.08 a | 0.05 c | 0.06 a | 0.09 a |
| **2** | **1** | **0** | 206.62 f | 8.81 e | 3.73 e | 12.54 e | 5.96 e | 7.94 d | 36.98 c | 0.03 j | 0.04 j | 0.03 g | 0.03 l | 0.04 k |
|  |  | **1** | 218.63 e | 9.70 d | 4.16 d | 13.84 d | 6.45 d | 7.65 de | 36.21 cd | 0.03 h | 0.04 i | 0.04 f | 0.03 k | 0.05 j |
|  |  | **2** | 232.99 d | 10.54 c | 4.58 c | 15.12 c | 7.05 c | 7.51 e | 35.32 d | 0.04 f | 0.05 g | 0.05 d | 0.04 i | 0.05 i |
|  |  | **0** | 141.40 k | 4.92 j | 1.74 i | 6.66 k | 3.30 i | 10.10 a | 48.44 a | 0.04 e | 0.06 e | 0.05 d | 0.04 g | 0.06 g |
|  | **2** | **1** | 155.55 j | 5.54 i | 1.88 hi | 7.42 j | 3.86 h | 8.98 b | 40.82 b | 0.04 c | 0.06 d | 0.05 b | 0.05 f | 0.06 f |
|  |  | **2** | 170.30 i | 6.07 h | 2.10 gh | 8.17 h | 4.45 g | 7.35 e | 30.84 e | 0.05 b | 0.08 b | 0.06 a | 0.05 c | 0.07 d |

**Table 13** (continued).

| **Traetment** | | | **So pro** | **Prol** | **Flavo** | **Phe** | **Sug** | **H_2_O_2_** | **RWC** | **N** | **Pro** | **Oil** | **SOY** | **SPY** |
| --- | --- | --- | --- | --- | --- | --- | --- | --- | --- | --- | --- | --- | --- | --- |
| **Year** | **Irr** | **ML** |  |  |  |  |  |  |  |  |  |  |  |  |
| **1** | **1** | **0** | 52.33 g | 24.31 k | 11.77 h | 19.23 i | 5.96 g | 6.85 gh | 78.09 c | 2.92 e | 18.29 e | 6.88 b | 104.26 b | 278.19 b |
|  |  | **1** | 58.33 f | 28.33 j | 13.90 j | 20.98 h | 7.21 f | 6.40 hi | 79.79 b | 3.38 dc | 21.16 dc | 7.73 a | 125.71 a | 344.83 a |
|  |  | **2** | 67.88 bc | 34.00 h | 17.11 h | 25.38 f | 9.26 e | 5.97 i | 81.38 a | 3.25 d | 20.32 d | 7.57 a | 129.71 a | 344.65 a |
|  | **2** | **0** | 64.88 d | 37.33 g | 18.11 g | 30.22 d | 8.97 e | 11.40 c | 46.07 j | 3.42 c | 21.43 c | 5.78 d | 42.25 e | 156.36 d |
|  |  | **1** | 68.77 b | 41.66 f | 21.22 d | 33.66 c | 11.12 dc | 10.55 d | 51.42 h | 3.85 a | 24.11 a | 6.64 cb | 55.44 dc | 200.53 c |
|  |  | **2** | 75.00 a | 48.77 c | 26.11 b | 39.33 a | 14.11 b | 9.33 e | 54.88 g | 3.62 b | 22.66 b | 6.42 c | 60.10 c | 210.68 c |
| **2** | **1** | **0** | 47.10 h | 30.01 i | 13.42 j | 16.59 j | 7.39 f | 7.93 f | 69.68 f | 1.55 h | 9.73 h | 4.72 f | 42.54 e | 87.96 h |
|  |  | **1** | 52.50 g | 34.78 h | 16.38 i | 19.30 i | 9.31 e | 7.33 g | 72.91 e | 1.90 g | 11.88 g | 5.25 e | 53.20 d | 121.07 f |
|  |  | **2** | 61.10 e | 42.48 e | 19.55 f | 21.82 g | 11.44 c | 6.82 gh | 75.57 d | 1.91 g | 11.99 g | 5.17 e | 57.95 dc | 134.43 e |
|  |  | **0** | 58.40 f | 45.88 d | 20.46 e | 26.26 e | 10.75 d | 13.27 a | 43.48 k | 2.06 g | 12.91 g | 3.57 h | 19.05 g | 69.07 i |
|  | **2** | **1** | 61.90 e | 52.19 b | 23.98 c | 30.62 d | 13.96 b | 12.27 b | 45.32 j | 2.53 f | 15.84 f | 4.05 g | 24.83 gf | 97.84 hg |
|  |  | **2** | 67.50 c | 59.50 a | 29.50 a | 34.39 b | 17.77 a | 11.00 cd | 48.38 i | 2.38 f | 14.93 f | 4.08 g | 27.56 f | 100.22 g |

**PH:** Plant height, **NMB:** Number of main branches, **NLB:** Number of lateral branches, **NSP:** Number of seeds per plant, **NPP:** Number of pods per plant, **PWP:** Pod weight per plant, **W100S:** 100-seed weight, **W100P:** 100-pod weight, **SSY:** Straw and stubble yield, **HI:** Harvest index, **MBD:** Main branch diameter, **PSP:** Percentage of seedless pods, **GY:** Grain Yield, **SC**: Stomatal Conductance, **Chl a:** Chlorophyll a, **Chl b:** Chlorophyll b, **Tot Chl:** Total chlorophyll, **Cart:** Carotenoid, **MDA**: Malondialdehyde, **EL**: Electrolyte leakage, **CAT:** Catalase, **SOD:** Superoxide dismutase, **GPX:** Guaiacol peroxidase, **APX:** Ascorbate peroxidase, **PPO:** Polyphenol oxidase, **Tot Pro**: Total soluble protein, **Prol**: Proline, **Flavo**: Flavonoid, **Phe**: Phenol, **Sug**: Sugar, **RW**C: Relative water content, **%N**: Seed nitrogen, **%Pro**: Seed protein, **%Oil:** Seed oil, **SOY:** Seed oil yield, **SPY:** Seed protein yield.

Mean comparison was performed by DUNCAN method at 5% probability. Columns with similar letters did not differ significantly

**Table S14.** The mean comparison of interaction effects of year**, d**ifferent levels of melatonin and 24-epibrassinolide on evaluated traits of chickpea plants

| **Traetment** | | | PH | **NMB** | NLB | **NSP** | **NPP** | **PWP** | W100S | **W100P** | **SSY** | **HI** | **MBD** | **PSP** | **GY** |
| --- | --- | --- | --- | --- | --- | --- | --- | --- | --- | --- | --- | --- | --- | --- | --- |
| **Year** | **EBL** | **ML** |  |  |  |  |  |  |  |  |  |  |  |  |  |
| **1** | **0** | **0** | 46.66 gf | 2.80 ef | 21.43 e | 55.77 fg | 40.46 f | 19.82 fg | 36.31 d | 42.48 gf | 100.00 j | 0.74 ba | 4.07 f | 7.58 c | 1041.49 f |
|  |  | **1** | 48.20 e | 2.89 ed | 22.70 d | 55.92 fg | 42.06 fe | 20.74 fe | 36.66 d | 43.45 fe | 106.33 i | 0.74 b | 4.31 ed | 6.67 fe | 1051.64 fe |
|  |  | **2** | 49.76 dc | 2.98 d | 23.63 c | 59.05 e | 43.86 dc | 21.85 dc | 38.16 c | 44.47 cde | 115.50 h | 0.73 b | 4.63 c | 5.77 hi | 1154.08 d |
|  | **1** | **0** | 48.40 e | 2.96 d | 23.41 dc | 58.01 fe | 42.86 de | 21.19 de | 36.85 d | 43.70 de | 107.66 i | 0.74 b | 4.36 d | 6.48 fg | 1100.95 e |
|  |  | **1** | 50.26 c | 3.12 c | 23.98 c | 63.23 cd | 45.16 c | 22.27 c | 38.42 c | 45.09 c | 114.50 h | 0.73 b | 4.70 c | 5.72 i | 1240.61 c |
|  |  | **2** | 51.60 b | 3.20 c | 25.03 b | 65.20 cb | 47.43 b | 23.53 b | 40.13 b | 46.34 b | 122.00 gf | 0.73 b | 4.98 b | 4.96 j | 1332.07 b |
|  | **2** | **0** | 49.20 de | 3.11 c | 24.06 c | 61.40 d | 43.76 cde | 22.37 c | 38.48 c | 44.64 dc | 116.66 gh | 0.74 b | 4.66 c | 5.69 i | 1210.95 c |
|  |  | **1** | 51.40 b | 3.31 b | 25.26 b | 65.83 b | 47.50 b | 23.82 b | 40.20 b | 46.37 b | 125.50 f | 0.76 a | 4.99 b | 4.83 j | 1358.71 b |
|  |  | **2** | 52.73 a | 3.59 a | 26.01 a | 69.67 a | 50.93 a | 25.26 a | 42.02 a | 48.93 a | 136.50 e | 0.70 c | 5.34 a | 4.32 k | 1498.32 a |
| **2** | **0** | **0** | 44.81 ji | 2.63 g | 17.93 k | 40.43 l | 28.13 l | 14.14 l | 31.66 g | 34.62 k | 135.00 e | 0.60 d | 3.52 i | 8.95 a | 650.61 j |
|  |  | **1** | 43.73 lk | 2.71 gf | 18.46 jk | 42.63 kl | 30.02 k | 15.06 lk | 32.05 g | 35.93 j | 143.33 d | 0.60 ed | 3.69 h | 8.13 b | 694.16 ji |
|  |  | **2** | 44.56 jk | 2.78 ef | 19.00 ji | 45.40 j | 31.91 ji | 16.29 ji | 33.36 f | 37.26 i | 155.66 c | 0.59 d-g | 3.95 g | 7.20 dc | 768.35 h |
|  | **1** | **0** | 43.76 lk | 2.63 g | 18.66 ji | 43.56 kj | 30.26 jk | 15.36 jk | 32.08 g | 35.89 j | 146.83 d | 0.59 def | 3.72 h | 8.12 b | 711.18 i |
|  |  | **1** | 45.26 hij | 2.78 ef | 19.35 ghi | 47.83 i | 32.82 i | 16.44 i | 33.56 f | 37.80 i | 158.33 c | 0.59 e-h | 3.96 gf | 7.05 de | 813.00 h |
|  |  | **2** | 46.06 gh | 2.91 d | 19.93 gf | 51.13 h | 34.94 h | 17.80 h | 35.23 e | 39.43 h | 171.83 b | 0.58 gh | 4.23 e | 6.17 hg | 912.67 g |
|  | **2** | **0** | 44.81 ji | 2.79 ef | 19.06 hij | 45.36 j | 32.45 i | 16.02 ijk | 33.88 f | 37.23 i | 158.33 c | 0.59 e-h | 3.97 gf | 6.99 de | 778.11 h |
|  |  | **1** | 45.63 ghi | 2.98 d | 19.78 gh | 50.56 h | 35.21 h | 17.71 h | 35.32 e | 39.73 h | 172.00 b | 0.58 fgh | 4.33 ed | 6.04 hi | 904.12 g |
|  |  | **2** | 47.13 f | 3.20 c | 20.53 f | 54.13 g | 38.17 g | 19.20 g | 37.02 d | 41.51 g | 186.16 a | 0.58 h | 4.59 c | 5.16 j | 1014.81 f |

**Table 14** (continued).

| **Traetment** | | | **SC** | **Chl a** | **Chl b** | **Tot Chl** | **Cart** | **MDA** | **EL** | **CAT** | **SOD** | **GPX** | **APX** | **PPO** |
| --- | --- | --- | --- | --- | --- | --- | --- | --- | --- | --- | --- | --- | --- | --- |
| **Year** | **EBL** | **ML** |  |  |  |  |  |  |  |  |  |  |  |  |
| **1** | **0** | **0** | 220.76 f | 7.27 hi | 2.85 gf | 10.12 ij | 5.00 gh | 8.73 bc | 37.50 de | 0.03 i | 0.05 k | 0.03 j | 0.04 j | 0.06 h |
|  |  | **1** | 233.33 e | 8.00 g | 3.05 ef | 11.05 h | 5.38 ef | 7.83 def | 34.00 f | 0.04 fgh | 0.05 i | 0.03 i | 0.04 gh | 0.06 f |
|  |  | **2** | 246.78 d | 8.68 de | 3.40 d | 12.08 def | 5.85 c | 6.77 hi | 28.50 h | 0.04 de | 0.06 hi | 0.04 gf | 0.05 d | 0.07 d |
|  | **1** | **0** | 235.42 e | 8.08 gf | 3.30 de | 11.38 hg | 5.45 def | 7.50 ef | 32.64 g | 0.04 gh | 0.05 hi | 0.03 i | 0.04 g | 0.06 f |
|  |  | **1** | 252.32 dc | 8.68 de | 3.57 cd | 12.26 de | 5.88 c | 6.98 hg | 29.40 h | 0.04 e | 0.06 g | 0.04 h | 0.05 e | 0.07 e |
|  |  | **2** | 269.49 b | 9.79 b | 3.96 b | 13.75 b | 6.60 b | 6.33 i | 25.15 i | 0.05 c | 0.07 d | 0.05 d | 0.059 b | 0.07 c |
|  | **2** | **0** | 254.22 c | 8.85 cd | 3.51 cd | 12.37 d | 5.90 c | 6.71 hi | 28.26 h | 0.04 de | 0.06 e | 0.04 f | 0.05 d | 0.07 d |
|  |  | **1** | 273.55 b | 9.97 b | 4.01 b | 13.99 b | 6.47 b | 6.33 i | 25.48 i | 0.05 bc | 0.07 c | 0.05 de | 0.06 b | 0.08 b |
|  |  | **2** | 292.33 a | 10.60 a | 4.43 a | 15.03 a | 7.31 a | 5.77 j | 22.43 j | 0.06 a | 0.08 a | 0.05 bc | 0.06 a | 0.08 a |
| **2** | **0** | **0** | 162.54 j | 6.18 k | 2.42 h | 8.60 l | 4.25 j | 10.26 a | 48.96 a | 0.03 j | 0.04 l | 0.03 i | 0.03 l | 0.05 l |
|  |  | **1** | 174.04 i | 6.88 j | 2.59 gh | 9.47 k | 4.80 hi | 9.21 b | 44.20 b | 0.03 i | 0.05 k | 0.04 f | 0.04 k | 0.05 k |
|  |  | **2** | 185.63 h | 7.38 h | 2.89 f | 10.27 i | 5.20 gf | 8.00 de | 37.39 de | 0.04 f | 0.05 hi | 0.05 d | 0.04 i | 0.06 i |
|  | **1** | **0** | 172.83 j | 6.90 ji | 2.80 gf | 9.70 jk | 4.63 i | 8.88 b | 42.44 c | 0.03 i | 0.05 k | 0.04 gh | 0.04 k | 0.05 k |
|  |  | **1** | 185.42 h | 7.49 h | 3.03 ef | 10.53 i | 5.18 gf | 8.25 cd | 38.22 d | 0.04 fgh | 0.05 j | 0.04 e | 0.04 j | 0.05 j |
|  |  | **2** | 201.79 g | 8.41 ef | 3.37 d | 11.78 fg | 5.73 dc | 7.47 fg | 32.69 fg | 0.04 d | 0.06 f | 0.05 bc | 0.05 f | 0.06 h |
|  | **2** | **0** | 186.66 h | 7.52 h | 2.98 f | 10.51 i | 5.01 gh | 7.93 def | 36.74 e | 0.04 fg | 0.05 h | 0.051 d | 0.04 hi | 0.06 ij |
|  |  | **1** | 201.80 g | 8.48 e | 3.41 d | 11.89 ef | 5.50 de | 7.48 fg | 33.13 fg | 0.04 d | 0.06 e | 0.05 b | 0.05 f | 0.06 h |
|  |  | **2** | 217.52 f | 9.11 c | 3.76 bc | 12.88 c | 6.33 b | 6.82 hi | 29.16 h | 0.05 b | 0.07 b | 0.06 a | 0.05 c | 0.07 e |

**Table 14** (continued).

| **Traetment** | | | **So pro** | **Prol** | **Flavo** | **Phe** | **Sug** | **H_2_O_2_** | **RWC** | **N** | **Pro** | **Oil** | **SOY** | **SPY** |
| --- | --- | --- | --- | --- | --- | --- | --- | --- | --- | --- | --- | --- | --- | --- |
| **Year** | **EBL** | **ML** |  |  |  |  |  |  |  |  |  |  |  |  |
| **1** | **0** | **0** | 50.83 l | 25.80 m | 11.83 l | 21.30 l | 5.55 h | 9.96 cd | 60.09 f | 2.80 | 17.51 d | 5.58 d | 60.14 f | 175.78 f |
|  |  | **1** | 57.16 i | 30.16 l | 14.50 j | 23.63 k | 7.08 g | 9.35 de | 61.70 e | 3.13 c | 19.60 c | 6.26 c | 68.15 e | 199.65 e |
|  |  | **2** | 65.50 ed | 36.33 i | 18.00 h | 27.92 f | 9.30 e | 8.43 fg | 63.86 d | 3.58 b | 22.40 b | 6.96 b | 83.04 d | 255.23 cd |
|  | **1** | **0** | 58.83 h | 29.66 l | 15.00 j | 24.15 jk | 7.30 g | 9.03 ef | 61.95 e | 3.11 c | 19.49 c | 6.41 c | 72.35 e | 206.27 e |
|  |  | **1** | 61.83 g | 33.83 j | 16.75 i | 26.21 h | 8.61 f | 8.45 fg | 66.25 c | 3.71 b | 23.19 b | 7.36 b | 93.37 cb | 284.03 b |
|  |  | **2** | 70.16 c | 39.83 g | 20.83 ef | 30.81 d | 11.26 dc | 7.65 h | 68.97 b | 4.02 a | 25.17 a | 7.83 a | 106.34 a | 330.38 a |
|  | **2** | **0** | 66.16 d | 37.00 hi | 18.00 h | 28.73 e | 9.56 e | 8.38 g | 64.21 d | 3.61 b | 22.57 b | 6.98 b | 87.28 cb | 269.78 cb |
|  |  | **1** | 71.66 b | 41.00 f | 21.43 e | 32.11 c | 11.80 c | 7.63 h | 68.86 b | 4.01 a | 25.12 a | 7.93 a | 110.20 a | 334.36 a |
|  |  | **2** | 78.66 a | 48.00 c | 26.00 b | 38.33 a | 14.50 b | 6.88 i | 71.56 a | 2.70 d | 16.90 d | 6.20 c | 95.35 b | 247.39 d |
| **2** | **0** | **0** | 45.75 m | 31.47 k | 13.37 k | 18.74 n | 6.88 g | 11.50 a | 54.06 i | 1.53 g | 9.56 g | 3.82 h | 25.86 k | 59.815 k |
|  |  | **1** | 51.45 l | 37.80 h | 17.05 i | 21.45 l | 9.36 e | 10.75 b | 56.02 h | 1.74 f | 10.93 f | 4.12 fgh | 29.80 jk | 73.51 kj |
|  |  | **2** | 58.95 h | 45.32 d | 20.34 f | 23.47 k | 11.37 cd | 10.00 c | 58.56 g | 2.06 e | 12.89 e | 4.45 efg | 35.27 ji | 95.88 i |
|  | **1** | **0** | 52.95 k | 36.72 i | 16.45 i | 20.59 m | 9.03 ef | 10.65 b | 56.83 h | 1.79 f | 11.19 f | 4.07 hg | 30.34 jk | 76.34 j |
|  |  | **1** | 55.65 j | 42.38 e | 18.92 g | 24.50 ij | 10.91 d | 9.83 cd | 59.48 fg | 2.23 e | 13.99 e | 4.59 e | 38.33 hi | 109.28 ih |
|  |  | **2** | 63.15 fg | 48.59 c | 23.54 d | 27.11 g | 14.13 b | 9.00 efg | 61.49 e | 2.61 d | 16.32 d | 5.18 d | 48.90 g | 144.39 g |
|  | **2** | **0** | 59.55 h | 45.64 d | 21.00 ef | 24.95 i | 11.31 cd | 9.66 cd | 58.84 g | 2.11 e | 13.20 e | 4.54 fe | 36.19 ji | 99.39 ih |
|  |  | **1** | 64.50 ef | 50.29 b | 24.57 c | 28.92 e | 14.63 b | 8.83 efg | 61.84 e | 2.66 d | 16.66 d | 5.25 d | 48.91 g | 145.57 g |
|  |  | **2** | 70.80 bc | 59.06 a | 29.71 a | 33.73 b | 18.31 a | 7.73 h | 65.87 c | 1.78 f | 11.18 f | 4.25 efg | 44.11 hg | 111.70 h |

**PH:** Plant height, **NMB:** Number of main branches, **NLB:** Number of lateral branches, **NSP:** Number of seeds per plant, **NPP:** Number of pods per plant, **PWP:** Pod weight per plant, **W100S:** 100-seed weight, **W100P:** 100-pod weight, **SSY:** Straw and stubble yield, **HI:** Harvest index, **MBD:** Main branch diameter, **PSP:** Percentage of seedless pods, **GY:** Grain Yield, **SC**: Stomatal Conductance, **Chl a:** Chlorophyll a, **Chl b:** Chlorophyll b, **Tot Chl:** Total chlorophyll, **Cart:** Carotenoid, **MDA**: Malondialdehyde, **EL**: Electrolyte leakage, **CAT:** Catalase, **SOD:** Superoxide dismutase, **GPX:** Guaiacol peroxidase, **APX:** Ascorbate peroxidase, **PPO:** Polyphenol oxidase, **Tot Pro**: Total soluble protein, **Prol**: Proline, **Flavo**: Flavonoid, **Phe**: Phenol, **Sug**: Sugar, **RW**C: Relative water content, **%N**: Seed nitrogen, **%Pro**: Seed protein, **%Oil:** Seed oil, **SOY:** Seed oil yield, **SPY:** Seed protein yield.

Mean comparison was performed by DUNCAN method at 5% probability. Columns with similar letters did not differ significantly

**Table S15.** The mean comparison of interaction effects of **d**ifferent levels of water deficit, 24-epibrassinolide, and melatonin on evaluated traits of chickpea plants

| **Traetment** | | | PH | **NMB** | NLB | **NSP** | **NPP** | **PWP** | W100S | **W100P** | **SSY** | **HI** | **MBD** | **PSP** | **GY** |
| --- | --- | --- | --- | --- | --- | --- | --- | --- | --- | --- | --- | --- | --- | --- | --- |
| **Irr** | **EBL** | **ML** |  |  |  |  |  |  |  |  |  |  |  |  |  |
| **1** | **0** | **0** | 46.86 ef | 3.01 g | 21.40 fgh | 58.53 f | 42.30 g | 21.51 f | 37.56 d | 41.39 fg | 129.66 gh | 0.70 a | 4.20 f | 6.93 c | 1115.84 g |
|  |  | **1** | 48.06 d | 3.09 fg | 22.00 efg | 59.69 f | 43.92 fg | 22.36 ef | 37.79 d | 42.63 de | 138.00 ef | 0.69 abc | 4.43 e | 6.25 d | 1141.69 g |
|  |  | **2** | 49.53 c | 3.20 de | 22.56 ed | 62.86 e | 45.81 de | 23.48 dc | 39.17 c | 43.64 cd | 149.83 dc | 0.68 bcd | 4.73 d | 5.62 e | 1245.71 ef |
|  | **1** | **0** | 47.83 de | 3.13 ef | 22.08 ef | 62.45 e | 44.76 ef | 22.75 de | 38.10 d | 42.40 ef | 139.00 e | 0.69 ab | 4.50 e | 6.31 d | 1205.55 f |
|  |  | **1** | 49.46 c | 3.23 de | 22.81 cd | 65.93 dc | 47.05 dc | 23.86 c | 39.31 c | 44.03 c | 150.33 c | 0.68 abc | 4.89 c | 5.63 e | 1311.58 d |
|  |  | **2** | 50.73 b | 3.35 bc | 23.66 b | 67.50 c | 48.84 b | 25.29 b | 41.40 b | 45.64 b | 161.16 b | 0.68 b-e | 5.09 b | 4.93 f | 1410.84 c |
|  | **2** | **0** | 49.25 c | 3.28 cd | 22.40 ed | 64.55 ed | 46.05 de | 23.71 dc | 39.39 c | 43.87 c | 150.00 c | 0.68 bc | 4.77 d | 5.56 e | 1290.33 de |
|  |  | **1** | 50.60 b | 3.45 b | 23.51 bc | 69.81 b | 48.51 bc | 25.41 b | 41.45 b | 45.72 b | 166.00 b | 0.68 cde | 5.11 b | 4.92 f | 1466.28 b |
|  |  | **2** | 52.23 a | 3.76 a | 24.46 a | 72.86 a | 51.37 a | 27.19 a | 43.62 a | 48.28 a | 178.83 a | 0.67 de | 5.50 a | 4.44 g | 1607.33 a |
| **2** | **0** | **0** | 42.73 h | 2.41 l | 17.96 l | 37.66 k | 26.30 m | 12.44 l | 30.42 h | 35.71 l | 105.33 l | 0.65 fg | 3.38 i | 9.60 a | 576.25 l |
|  |  | **1** | 43.86 g | 2.51 kl | 19.16 k | 38.86 k | 28.16 l | 13.44 k | 30.92 h | 36.75 kl | 111.66 k | 0.65 g | 3.57 h | 8.55 b | 604.10 l |
|  |  | **2** | 44.80 g | 2.56 jk | 20.06 ij | 41.59 j | 29.96 jk | 14.66 ji | 32.35 g | 38.09 ij | 121.33 ij | 0.64 gh | 3.84 g | 7.36 c | 676.71 k |
|  | **1** | **0** | 44.33 g | 2.46 kl | 20.00 j | 39.13 k | 28.36 kl | 13.80 jk | 30.82 h | 37.20 jk | 105.33 l | 0.64 gh | 3.59 h | 8.28 b | 606.58 l |
|  |  | **1** | 46.06 f | 2.67 ij | 20.51 ij | 45.13 i | 30.93 j | 14.85 i | 32.66 g | 38.87 i | 111.66 k | 0.64 gh | 3.77 g | 7.14 c | 742.03 j |
|  |  | **2** | 46.93 ef | 2.76 hi | 21.30 hg | 48.83 gh | 33.53 i | 16.04 h | 33.96 f | 40.13 h | 121.33 ij | 0.63 h | 4.11 f | 6.20 d | 833.90 i |
|  | **2** | **0** | 44.76 g | 2.63 j | 20.73 hi | 42.21 j | 30.16 j | 14.68 ij | 32.97 g | 38.01 ij | 125.00 hi | 0.65 gh | 3.85 g | 7.12 c | 698.74 jk |
|  |  | **1** | 46.43 f | 2.85 h | 21.53 fg | 46.59 hi | 34.20 i | 16.12 h | 34.07 f | 40.39 hg | 131.50 g | 0.67 ef | 4.21 f | 5.95 de | 796.55 i |
|  |  | **2** | 47.63 de | 3.03 fg | 22.08 ef | 50.93 g | 37.73 h | 17.27 g | 35.42 e | 42.15 ef | 143.83 de | 0.61 i | 4.42 e | 5.04 f | 905.80 h |

**Table 15** (continued).

| **Traetment** | | | **SC** | **Chl a** | **Chl b** | **Tot chl** | **Cart** | **MDA** | **EL** | **CAT** | **SOD** | **GPX** | **APX** | **PPO** |
| --- | --- | --- | --- | --- | --- | --- | --- | --- | --- | --- | --- | --- | --- | --- |
| **Irr** | **EBL** | **ML** |  |  |  |  |  |  |  |  |  |  |  |  |
| **1** | **0** | **0** | 235.04 e | 8.67 e | 3.54 e | 12.21 g | 6.10 e | 7.84 de | 33.56 ef | 0.02 i | 0.03 m | 0.03 l | 0.03 l | 0.04 n |
|  |  | **1** | 247.92 d | 9.48 d | 3.79 e | 13.28 f | 6.53 d | 7.29 fgh | 32.96 efg | 0.03 h | 0.04 l | 0.03 k | 0.03 k | 0.05 m |
|  |  | **2** | 260.94 c | 10.25 c | 4.31 dc | 14.56 d | 6.95 c | 7.11 hig | 31.83 hig | 0.04 f | 0.04 j | 0.04 ij | 0.04 i | 0.05 k |
|  | **1** | **0** | 249.52 d | 9.56 d | 4.22 d | 13.78 e | 6.47 d | 7.27 f-i | 32.53 fgh | 0.03 h | 0.04 l | 0.03 k | 0.03 k | 0.05 m |
|  |  | **1** | 262.33 c | 10.36 c | 4.56 c | 14.93 cd | 6.96 c | 7.16 ghi | 32.04 hig | 0.03 g | 0.04 k | 0.04 j | 0.04 j | 0.05 l |
|  |  | **2** | 277.90 b | 11.72 b | 5.05 b | 16.78 b | 7.58 b | 7.01 hi | 31.40 hij | 0.04 e | 0.05 i | 0.04 fg | 0.04 g | 0.06 j |
|  | **2** | **0** | 264.23 c | 10.58 c | 4.40 cd | 14.99 c | 6.88 c | 7.12 ghi | 31.89 ghi | 0.03 g | 0.04 j | 0.04 hi | 0.04 h | 0.05 k |
|  |  | **1** | 276.25 b | 11.71 b | 5.18 b | 16.89 b | 7.33 b | 6.94 hi | 31.09 ij | 0.04 de | 0.05 i | 0.04 g | 0.04 g | 0.06 i |
|  |  | **2** | 294.02 a | 12.37 a | 5.58 a | 17.95 a | 8.22 a | 6.82 hi | 30.51 j | 0.05 c | 0.06 g | 0.05 e | 0.05 e | 0.07 f |
| **2** | **0** | **0** | 148.26 j | 4.77 k | 1.72 j | 6.50 l | 3.14 j | 11.14 a | 52.90 a | 0.04 f | 0.05 h | 0.04 h | 0.04 g | 0.06 h |
|  |  | **1** | 159.45 i | 5.39 j | 1.85 ij | 7.24 k | 3.65 i | 9.75 b | 45.23 b | 0.04 d | 0.06 f | 0.04 f | 0.05 f | 0.07 g |
|  |  | **2** | 171.47 h | 5.81 h | 1.97 hij | 7.79 j | 4.10 h | 7.66 def | 34.06 e | 0.05 c | 0.07 d | 0.05 d | 0.05 c | 0.07 d |
|  | **1** | **0** | 158.72 i | 5.42 ij | 1.88 ij | 7.31 k | 3.60 i | 9.12 c | 42.55 c | 0.04 d | 0.06 f | 0.04 fg | 0.05 f | 0.07 fg |
|  |  | **1** | 175.40 h | 5.81 h | 2.04 ghi | 7.86 j | 4.09 h | 8.08 d | 35.57 d | 0.05 c | 0.07 e | 0.05 e | 0.05 d | 0.07 e |
|  |  | **2** | 193.38 g | 6.47 g | 2.28 g | 8.75 i | 4.74 g | 6.79 i | 26.45 k | 0.05 b | 0.08 c | 0.06 c | 0.06 b | 0.08 c |
|  | **2** | **0** | 176.65 h | 5.80 hi | 2.09 ghi | 7.89 j | 4.03 h | 7.52 efg | 33.12 efg | 0.05 c | 0.07 d | 0.05 d | 0.05 c | 0.07 d |
|  |  | **1** | 199.11 g | 6.74 g | 2.25 gh | 8.99 i | 4.64 g | 6.86 hi | 27.52 k | 0.05 b | 0.08 b | 0.06 b | 0.06 b | 0.08 b |
|  |  | **2** | 215.84 f | 7.35 f | 2.62 f | 9.97 h | 5.41 f | 5.78 j | 21.08 l | 0.06 a | 0.09 a | 0.06 a | 0.07 a | 0.09 a |

**Table 15** (continued).

| **Traetment** | | | **So pro** | **Prol** | **Flavo** | **Phe** | **Sug** | **H_2_O_2_** | **RWC** | **N** | **Pro** | **Oil** | **SOY** | **SPY** |
| --- | --- | --- | --- | --- | --- | --- | --- | --- | --- | --- | --- | --- | --- | --- |
| **Irr** | **EBL** | **ML** |  |  |  |  |  |  |  |  |  |  |  |  |
| **1** | **0** | **0** | 39.90 o | 21.38 o | 9.94 l | 15.60 m | 4.66 j | 7.96 f | 72.05 f | 1.91 g | 11.98 g | 5.26 fg | 61.27 e | 144.48 hi |
|  |  | **1** | 47.81 n | 26.27 n | 12.73 j | 17.02 l | 6.82 i | 7.51 gf | 73.40 e | 2.19 f | 13.70 f | 5.81 de | 69.25 d | 167.14 gf |
|  |  | **2** | 58.58 j | 33.93 l | 15.26 h | 19.76 k | 8.27 gh | 7.10 gh | 75.64 d | 2.62 d | 16.37 d | 6.33 bc | 82.67 c | 218.48 c |
|  | **1** | **0** | 50.35 m | 26.42 n | 11.92 k | 17.04 l | 6.64 i | 7.50 fg | 73.99 e | 2.17 f | 13.56 f | 5.79 de | 72.94 d | 174.69 ef |
|  |  | **1** | 53.83 l | 31.04 m | 14.02 i | 19.65 k | 7.96 h | 6.78 hi | 76.99 c | 2.71 d | 16.95 d | 6.48 b | 89.24 bc | 238.05 b |
|  |  | **2** | 62.38 gh | 37.37 j | 17.39 g | 22.84 i | 10.09 f | 6.31 ij | 78.42 b | 3.04 cb | 19.04 bc | 7.11 a | 103.84 a | 281.34 a |
|  | **2** | **0** | 58.90 j | 33.67 l | 15.93 h | 21.09 j | 8.73 g | 6.71 hi | 75.61 d | 2.63 d | 16.48 d | 6.32 bc | 86.00 c | 230.06 bc |
|  |  | **1** | 64.60 ef | 37.37 j | 18.67 f | 23.73 h | 10.00 f | 6.30 ij | 78.65 b | 3.02 c | 18.91 c | 7.19 a | 109.88 a | 293.67 a |
|  |  | **2** | 72.51 b | 43.42 g | 22.34 cd | 28.20 f | 12.69 d | 5.78 j | 81.36 a | 2.09 fg | 13.06 fg | 5.68 def | 94.99 b | 218.80 c |
| **2** | **0** | **0** | 56.68 k | 35.89 k | 15.26 h | 24.44 g | 7.76 h | 13.50 a | 42.10 l | 2.41 e | 15.09 e | 4.13 j | 24.73 k | 91.12 k |
|  |  | **1** | 60.80 i | 41.70 h | 18.81 ef | 28.07 f | 9.62 f | 12.58 b | 44.32 k | 2.69 d | 16.82 d | 4.57 i | 28.71 jk | 106.03 j |
|  |  | **2** | 65.86 de | 47.73 e | 23.07 c | 31.62 e | 12.40 d | 11.33 c | 46.77 j | 3.02 c | 18.92 c | 5.08 gh | 28.71 ij | 132.63 i |
|  | **1** | **0** | 61.43 hi | 39.96 i | 19.52 e | 27.70 f | 9.69 f | 12.18 b | 44.78 k | 2.74 d | 17.12 d | 4.70 hi | 29.76 jk | 107.92 j |
|  |  | **1** | 63.65 fg | 45.17 f | 21.65 d | 31.06 e | 11.57 e | 11.50 c | 48.73 i | 3.23 b | 20.23 b | 5.47 efg | 42.46 ghi | 155.27 gh |
|  |  | **2** | 70.93 c | 51.06 c | 26.98 b | 35.09 c | 15.30 c | 10.33 d | 52.05 h | 3.59 a | 22.45 a | 5.89 d | 51.40 f | 193.43 d |
|  | **2** | **0** | 66.81 d | 48.97 d | 23.07 c | 32.58 d | 12.14 d | 11.33 c | 47.45 j | 3.08 bc | 19.30 bc | 5.20 g | 37.47 hi | 139.11 i |
|  |  | **1** | 71.56 bc | 53.92 b | 27.33 b | 37.30 b | 16.42 b | 10.16 d | 52.05 h | 3.65 a | 22.87 a | 5.99 cd | 49.24 fg | 186.26 de |
|  |  | **2** | 76.95 a | 63.64 a | 33.37 a | 43.86 a | 20.12 a | 8.83 e | 56.08 g | 2.40 e | 15.02 e | 4.77 hi | 44.47 fgh | 140.30 i |

**PH:** Plant height, **NMB:** Number of main branches, **NLB:** Number of lateral branches, **NSP:** Number of seeds per plant, **NPP:** Number of pods per plant, **PWP:** Pod weight per plant, **W100S:** 100-seed weight, **W100P:** 100-pod weight, **SSY:** Straw and stubble yield, **HI:** Harvest index, **MBD:** Main branch diameter, **PSP:** Percentage of seedless pods, **GY:** Grain Yield, **SC**: Stomatal Conductance, **Chl a:** Chlorophyll a, **Chl b:** Chlorophyll b, **Tot Chl:** Total chlorophyll, **Cart:** Carotenoid, **MDA**: Malondialdehyde, **EL**: Electrolyte leakage, **CAT:** Catalase, **SOD:** Superoxide dismutase, **GPX:** Guaiacol peroxidase, **APX:** Ascorbate peroxidase, **PPO:** Polyphenol oxidase, **Tot Pro**: Total soluble protein, **Prol**: Proline, **Flavo**: Flavonoid, **Phe**: Phenol, **Sug**: Sugar, **RW**C: Relative water content, **%N**: Seed nitrogen, **%Pro**: Seed protein, **%Oil:** Seed oil, **SOY:** Seed oil yield, **SPY:** Seed protein yield.

Mean comparison was performed by DUNCAN method at 5% probability. Columns with similar letters did not differ significantly

**Table S16.** The mean comparison of interaction effects of different levels of water deficit, 24-epibrassinolide, melatonin, and year on evaluated traits of chickpea plants

| **Traetment** | | | | **PH** | **NMB** | **NLB** | **NSP** | **NPP** | **PWP** | **W100S** | **W100P** | **SSY** | **HI** | **MBD** | **PSP** | **GY** |
| --- | --- | --- | --- | --- | --- | --- | --- | --- | --- | --- | --- | --- | --- | --- | --- | --- |
| **Year** | **Irr** | **EBL** | **ML** |  |  |  |  |  |  |  |  |  |  |  |  |  |
| **1** | **1** | **0** | **0** | 49.26 fg | 3.06 g-j | 23.60 fgh | 69.34 ef | 52.86 f | 24.78 gh | 40.56 ef | 45.74 d | 108.33 nop | 0.77 ab | 4.46 g | 6.19 j | 1406.78 e |
|  |  |  | **1** | 51.06 de | 3.11 f-i | 24.33 def | 68.78 f | 54.53 ef | 25.48 fg | 40.78 ef | 46.60 cd | 116.00 l-o | 0.76 abc | 4.70 ef | 5.49 kl | 1402.70 e |
|  |  |  | **2** | 53.06 bc | 3.23 ef | 25.00 cde | 72.39 de | 56.26 cde | 26.31 def | 42.20 cd | 47.44 bc | 126.33 jk | 0.75 cd | 5.00 d | 4.90 lmn | 1527.73 d |
|  |  | **1** | **0** | 50.13 ef | 3.26 def | 24.16 ef | 72.83 cd | 55.46 de | 25.82 efg | 41.19 de | 46.43 cd | 117.00 kmn | 0.76 abc | 4.82 e | 5.50 kl | 1499.75 d |
|  |  |  | **1** | 52.33 cd | 3.33 cde | 24.96 cde | 76.00 bc | 57.73 bcd | 27.30 cd | 42.48 cd | 47.68 bc | 124.00 jkl | 0.76 abc | 5.19 c | 4.84 mn | 1613.42 c |
|  |  |  | **2** | 54.00 ab | 3.46 bc | 26.06 ab | 74.67 bcd | 59.33 ab | 28.77 b | 44.98 b | 48.83 b | 131.66 ij | 0.75 bc | 5.38 b | 4.39 no | 1680.58 c |
|  |  | **2** | **0** | 52.20 cd | 3.40 cd | 24.66 de | 76.31 b | 56.00 de | 26.94 de | 42.56 c | 47.52 bc | 126.00 jk | 0.75 cd | 5.10 cd | 4.84 mn | 1623.06 c |
|  |  |  | **1** | 54.00 ab | 3.56 b | 25.86 abc | 80.68 a | 58.53 abc | 28.67 bc | 44.97 b | 48.69 b | 142.00 h | 0.74 cd | 5.41 b | 4.40 no | 1814.30 b |
|  |  |  | **2** | 55.26 a | 3.93 a | 26.70 a | 82.40 a | 60.66 a | 30.49 a | 47.44 a | 51.42 a | 154.00 ef | 0.73 def | 5.91 a | 4.05 o | 1954.51 a |
|  | **2** | **0** | **0** | 44.06 n-q | 2.53 qr | 19.26 nop | 42.20 pq | 28.06 qr | 14.85 tu | 32.07 lm | 39.22 mno | 91.66 r | 0.72 ef | 3.68 l | 8.96 b | 676.18 pq |
|  |  |  | **1** | 45.33 k-o | 2.66 opq | 21.06 kl | 43.06 op | 29.60 opq | 16.01 st | 32.54 l | 40.30 j-m | 96.66 rq | 0.72 f | 3.91 k | 7.86 cd | 700.56 op |
|  |  |  | **2** | 46.46 jkl | 2.73 nop | 22.26 ij | 45.72 no | 31.46 m-p | 17.40 qrs | 34.13 k | 41.51 h-k | 104.66 pq | 0.71 f | 4.25 h | 6.64 g-j | 780.42 n |
|  |  | **1** | **0** | 46.66 jk | 2.66 opq | 22.66 hi | 43.19 pq | 30.26 n-q | 16.57 rs | 32.51 l | 40.98 i-l | 98.33 qr | 0.72 f | 3.91 k | 7.46 def | 702.15 op |
|  |  |  | **1** | 48.20 ghi | 2.90 klm | 23.00 ghi | 50.47 lm | 32.60 k-n | 17.24 qrs | 34.36 k | 42.51 gh | 105.00 pq | 0.71 f | 4.21 hi | 6.61 g-j | 867.80 klm |
|  |  |  | **2** | 49.20 fg | 2.93 j-m | 24.00 efg | 55.73 jk | 35.53 ij | 18.29 n-q | 35.28 h-k | 43.85 efg | 112.33 m-p | 0.71 f | 4.57 fg | 5.52 k | 983.55 hi |
|  |  | **2** | **0** | 46.20 jkl | 2.83 lmn | 23.46 fgh | 46.48 n | 31.53 m-p | 17.79 pqr | 34.40 k | 41.77 hij | 107.33 op | 0.73 ef | 4.21 hi | 6.54 g-j | 798.84 mn |
|  |  |  | **1** | 48.80 fgh | 3.06 g-j | 24.66 de | 50.98 l | 36.46 hi | 18.97 m-p | 35.43 h-k | 44.05 ef | 109.00 nop | 0.77 a | 4.57 fg | 5.27 klm | 903.12 jk |
|  |  |  | **2** | 50.20 ef | 3.26 def | 25.33 bcd | 56.93 ij | 41.20 g | 20.04 klm | 36.60 gh | 46.44 cd | 119.00 klm | 0.67 g | 4.77 e | 4.60 no | 1042.12 h |
| **2** | **1** | **0** | **0** | 44.46 n-q | 2.96 i-l | 19.20 nop | 47.73 mn | 31.73 l-p | 18.24 opq | 34.55 k | 37.04 pq | 151.00 fg | 0.62 h | 3.95 jk | 7.68 cde | 824.89 lmn |
|  |  |  | **1** | 45.06 l-o | 3.06 g-j | 19.66 mno | 50.60 lm | 33.32 j-m | 19.23 l-o | 34.80 jk | 38.66 no | 160.00 de | 0.62 hi | 4.16 hi | 7.02 fgh | 880.68 kl |
|  |  |  | **2** | 46.00 j-m | 3.16 fg | 21.26 jk | 53.33 kl | 35.36 ij | 20.65 jk | 36.14 hij | 39.85 lmn | 173.33 c | 0.61 hi | 4.46 g | 6.33 ij | 963.68 ij |
|  |  | **1** | **0** | 45.53 k-n | 3.00 h-k | 20.00 mn | 52.06 l | 34.06 il | 19.68 k-n | 35.01 ijk | 38.37 nop | 161.00 de | 0.62 hi | 4.19 hi | 7.13 efg | 911.34 jk |
|  |  |  | **1** | 46.60 jk | 3.13 fgh | 20.66 klm | 55.86 ijk | 36.38 hi | 20.41 jkl | 36.15 hi | 40.38 j-m | 176.66 c | 0.61 hi | 4.59 fg | 6.42 hij | 1009.74 hi |
|  |  |  | **2** | 47.46 hij | 3.23 ef | 21.26 ij | 60.33 gh | 38.35 h | 21.81 ij | 37.83 g | 42.45 ghi | 190.66 b | 0.60 ij | 4.81 e | 5.48 kl | 1141.10 g |
|  |  | **2** | **0** | 46.30 jkl | 3.16 fg | 20.13 lmn | 52.80 kl | 36.10 hi | 20.48 jkl | 36.22 hi | 40.22 klm | 174.00 c | 0.62 hi | 4.44 g | 6.28 ij | 957.59 ij |
|  |  |  | **1** | 47.20 ij | 3.33 cde | 21.16 kl | 58.93 hi | 38.49 h | 22.15 i | 37.93 g | 42.75 fgh | 190.00 b | 0.61 hi | 4.81 e | 5.44 klm | 1118.26 g |
|  |  |  | **2** | 49.20 fg | 3.60 b | 22.23 ij | 63.33 g | 42.09 g | 23.89 h | 39.79 f | 45.15 de | 203.66 a | 0.61 hi | 5.10 cd | 4.84 mn | 1260.15 f |
|  | **2** | **0** | **0** | 41.40 s | 3.30 st | 16.66 s | 33.13 u | 24.53 s | 10.03 a | 28.77 p | 32.20 u | 119.00 klm | 0.58 jk | 3.09 q | 10.23 a | 476.32 t |
|  |  |  | **1** | 42.40 rs | 2.36 st | 17.26 rs | 34.66 tu | 26.73 rs | 10.88 az | 29.30 op | 33.20 tu | 126.66 jk | 0.58 kl | 3.23 pq | 9.25 b | 507.64 st |
|  |  |  | **2** | 43.13 qr | 2.40 rst | 17.86 qr | 37.46 rst | 28.46 qr | 11.92 xyz | 30.57 no | 34.67 st | 138.00 hi | 0.57 klm | 3.44 no | 8.07 c | 573.00 rs |
|  |  | **1** | **0** | 42.00 rs | 2.26 t | 17.33 rs | 35.06 stu | 26.46 rs | 11.03 az | 29.14 p | 33.42 tu | 132.66 ij | 0.57 k-n | 3.26 p | 9.11 b | 511.01 st |
|  |  |  | **1** | 43.93 opq | 2.43 rs | 18.03 qr | 39.80 qr | 29.26 qr | 12.46 wxy | 30.97 mn | 35.23 rs | 140.00 hi | 0.57 k-n | 3.34 op | 7.68 cde | 616.26 qr |
|  |  |  | **2** | 44.66 m-p | 2.60 pq | 18.60 pq | 41.93 pq | 31.53 m-p | 13.80 uvm | 32.63 l | 36.41 qr | 153.00 ef | 0.56 mn | 3.65 lm | 6.87 f-i | 684.24 pq |
|  |  | **2** | **0** | 43.33 pqr | 2.42 rs | 18.00 qr | 37.93 rs | 28.80 qr | 11.56 yz | 31.53 lmn | 34.24 st | 142.66 gh | 0.56 lmn | 3.50 mn | 7.70 cde | 598.64 r |
|  |  |  | **1** | 44.06 n-q | 2.63 pq | 18.40 pq | 42.20 pq | 31.93 k-o | 13.28 vwx | 32.71 l | 36.72 q | 154.00 ef | 0.56 mn | 3.84 k | 6.64 g-j | 689.97 p |
|  |  |  | **2** | 45.06 l-o | 2.80 mno | 18.83 opq | 44.93 nop | 34.26 jkl | 14.51 uv | 34.25 k | 37.87 opq | 168.66 cd | 0.55 n | 4.08 ij | 5.49 kl | 769.47 op |

**Table 16** (continued).

| **Traetment** | | | | **SC** | **Chl a** | **Chl b** | **Tot Chl** | **Cart** | **MDA** | **EL** | **CAT** | **SOD** | **GPX** | **APX** | **PPO** |
| --- | --- | --- | --- | --- | --- | --- | --- | --- | --- | --- | --- | --- | --- | --- | --- |
| **Year** | **Irr** | **EBL** | **ML** |  |  |  |  |  |  |  |  |  |  |  |  |
| **1** | **1** | **0** | **0** | 274.10 f | 9.37 h | 3.83 gh | 13.20 hi | 6.60 f-i | 7.33 g-j | 29.00 nop | 0.03 n | 0.04 t | 0.02 p | 0.03 r | 0.05 p |
|  |  |  | **1** | 287.23 e | 10.16 fg | 4.10 g | 14.26 g | 6.93 fg | 6.80 i-l | 28.66 n-q | 0.03 lm | 0.04 q | 0.03 o | 0.04 pq | 0.05 q |
|  |  |  | **2** | 301.03 cd | 11.08 dc | 4.66 e | 15.75 e | 7.38 de | 6.57 kl | 27.68 o-r | 0.042 gh | 0.05 mn | 0.03 mn | 0.04 lm | 0.06 j |
|  |  | **1** | **0** | 293.78 de | 10.34 ef | 4.56 ef | 14.90 f | 7.00 ef | 6.71 jkl | 28.29 n-r | 0.03 lm | 0.04 pq | 0.03 o | 0.04 p | 0.06 m |
|  |  |  | **1** | 306.54 c | 11.20 dc | 4.93 de | 16.14 cd | 7.43 d | 6.61 kl | 27.86 o-r | 0.04 hi | 0.04 op | 0.03 n | 0.04 n | 0.06 kl |
|  |  |  | **2** | 321.26 b | 12.62 b | 5.46 bc | 18.09 b | 8.16 b | 6.48 kl | 27.30 pqr | 0.04 e | 0.05 l | 0.04 j | 0.05 i | 0.07 h |
|  |  | **2** | **0** | 309.84 c | 11.46 c | 4.76 de | 16.23 cd | 7.44 d | 6.58 kl | 27.73 o-r | 0.04 hi | 0.05 m | 0.04 m | 0.04 kl | 0.06 j |
|  |  |  | **1** | 323.35 b | 12.66 b | 5.60 b | 18.26 b | 7.93 bc | 6.42 kl | 27.04 qr | 0.04 de | 0.05 l | 0.04 jkl | 0.05 i | 0.07 gh |
|  |  |  | **2** | 344.44 a | 13.37 a | 6.03 a | 19.40 a | 8.83 a | 6.30 kl | 26.53 r | 0.05 c | 0.06 j | 0.05 i | 0.05 ef | 0.07 e |
|  | **2** | **0** | **0** | 167.42 p | 5.16 o | 1.86 m-q | 7.03 rst | 3.40 s | 10.13 b | 46.00 d | 0.04 fg | 0.06 j | 0.04 m | 0.05 ij | 0.07 f |
|  |  |  | **1** | 179.42 o | 5.83 mln | 2.00 m-p | 7.83 opq | 3.83 qr | 8.86 c | 39.33 ef | 0.04 de | 0.06 hi | 0.04 jk | 0.05 h | 0.07 e |
|  |  |  | **2** | 192.53 n | 6.29 l | 2.13 k-o | 8.42 nm | 4.33 o | 6.96 h-k | 29.33 mno | 0.05 c | 0.07 de | 0.05 gh | 0.06 d | 0.08 c |
|  |  | **1** | **0** | 177.05 op | 5.83 mnl | 2.03 m-p | 7.86 nop | 3.90 qp | 8.29 cde | 37.00 g-k | 0.04 d | 0.07 h | 0.04 j | 0.056 h | 0.07 e |
|  |  |  | **1** | 198.10 mn | 6.16 l | 2.21 k-n | 8.38 mno | 4.33 o | 7.34 g-j | 30.93 m | 0.05 c | 0.07 f | 0.05 i | 0.05 e | 0.08 d |
|  |  |  | **2** | 217.71 jk | 6.95 k | 2.46 jk | 9.42 l | 5.03 n | 6.17 l | 23.00 s | 0.06 b | 0.08 c | 0.05 ef | 0.06 b | 0.08 b |
|  |  | **2** | **0** | 198.60 mn | 6.25 l | 2.26 klm | 8.51 m | 4.36 o | 6.84 i-l | 28.80 n-q | 0.05 c | 0.08 d | 0.05 gh | 0.06 d | 0.08 c |
|  |  |  | **1** | 223.76 ij | 7.29 k | 2.43 jkl | 9.72 l | 5.02 n | 6.23 l | 23.93 s | 0.06 b | 0.08 b | 0.05 ef | 0.06 b | 0.09 b |
|  |  |  | **2** | 240.22 g | 7.83 j | 2.83 j | 10.66 k | 5.80 lm | 5.25 m | 18.33 t | 0.07 a | 0.09 a | 0.06 c | 0.07 a | 0.09 a |
| **2** | **1** | **0** | **0** | 195.98 mn | 7.96 j | 3.25 i | 11.22 k | 5.61 m | 8.36 cd | 38.12 fgh | 0.02 o | 0.03 u | 0.03 o | 0.03 t | 0.04 t |
|  |  |  | **1** | 208.61 kl | 8.80 i | 3.48 hi | 12.29 j | 6.13 jkl | 7.78 d-g | 37.26 g-j | 0.03 n | 0.04 st | 0.03 mn | 0.03 s | 0.04 s |
|  |  |  | **2** | 220.86 ij | 9.42 h | 3.96 g | 13.38 h | 6.52 g-j | 7.65 e-h | 35.98 i-l | 0.03 jk | 0.045qr | 0.04 kl | 0.04 pq | 0.05 q |
|  |  | **1** | **0** | 205.26 lm | 8.78 i | 3.88 gh | 12.66 ij | 5.95 klm | 7.82 d-g | 36.78 h-k | 0.03 n | 0.04 t | 0.03 n | 0.03 rs | 0.04 s |
|  |  |  | **1** | 218.13 jk | 9.52 h | 4.19 fg | 13.72 gh | 6.49 hij | 7.70 d-g | 36.22 i-l | 0.03 lm | 0.04 rs | 0.04 l | 0.03 q | 0.05 r |
|  |  |  | **2** | 234.53 gh | 10.83 ed | 4.64 e | 15.47 ef | 7.00 ef | 7.54 fgh | 35.49 jkl | 0.04 fgh | 0.04 no | 0.05 hi | 0.04 o | 0.05 o |
|  |  | **2** | **0** | 218.61 j | 9.70 hg | 4.05 g | 13.75 de | 6.32 ijk | 7.66 d-h | 36.05 i-l | 0.03 kl | 0.04 q | 0.04jkl | 0.04 p | 0.05 q |
|  |  |  | **1** | 229.15 hi | 10.76 ed | 4.76 de | 15.52 e | 6.74 fgh | 7.47 ghi | 35.15 kl | 0.04 fg | 0.05 mno | 0.05 i | 0.04 o | 0.05 no |
|  |  |  | **2** | 243.59 g | 11.36 c | 5.12 cd | 16.49 c | 7.62 cd | 7.34 g-j | 34.49 l | 0.04 d | 0.05 k | 0.05 ef | 0.04 kl | 0.06 j |
|  | **2** | **0** | **0** | 129.10 s | 4.39 p | 1.58 q | 5.97 u | 2.89 t | 12.16 a | 59.80 a | 0.03 ij | 0.05 l | 0.04 j | 0.04 o | 0.05 mn |
|  |  |  | **1** | 139.48 r | 4.95 o | 1.70 pq | 6.65 t | 3.46 rs | 10.64 b | 51.13 b | 0.04 f | 0.06 j | 0.05 g | 0.04 mn | 0.06 l |
|  |  |  | **2** | 150.41 q | 5.34 no | 1.81 n-q | 7.16 rst | 3.88 pq | 8.36 cd | 38.80 efg | 0.04 f | 0.07 fg | 0.05 d | 0.05 i | 0.06 i |
|  |  | **1** | **0** | 140.39 r | 5.02 o | 1.72 opq | 6.75 st | 3.31 s | 9.95 b | 48.10 c | 0.04 f | 0.06 j | 0.05 hi | 0.04 mn | 0.06 jk |
|  |  |  | **1** | 152.71 q | 5.47 mno | 1.88 m-q | 7.35 pqr | 3.86 pqr | 8.81 c | 40.21 e | 0.04 de | 0.06 i | 0.05 f | 0.05 jk | 0.06 i |
|  |  |  | **2** | 169.04 p | 5.99 ml | 2.09 k-q | 8.09 mno | 4.45 o | 7.41 g-j | 29.90 mn | 0.05 c | 0.07 e | 0.06 c | 0.05 i | 0.07 g |
|  |  | **2** | **0** | 154.70 q | 5.35 no | 1.92 m-q | 7.27 qrs | 3.71 qrs | 8.20 c-f | 37.44 hig | 0.04 d | 0.07 g | 0.05 de | 0.05 i | 0.06 i |
|  |  |  | **1** | 174.46 op | 6.19 l | 2.06 k-p | 8.26 nmo | 4.26 op | 7.48 ghi | 31.11 m | 0.05 c | 0.08 d | 0.06 b | 0.05 fg | 0.07 f |
|  |  |  | **2** | 191.45 n | 6.86 k | 2.40 kl | 9.27 l | 5.03 n | 6.30 kl | 23.83 s | 0.06 b | 0.08 b | 0.07 a | 0.06 c | 0.08 d |

**Table 16** (continued).

| **Traetment** | | | | **So pro** | **Prol** | **Flavo** | **Phe** | **Sug** | **H_2_O_2_** | **RWC** | **N** | **Pro** | **Oil** | **SOY** | **SPY** |
| --- | --- | --- | --- | --- | --- | --- | --- | --- | --- | --- | --- | --- | --- | --- | --- |
| **Year** | **Irr** | **EBL** | **ML** |  |  |  |  |  |  |  |  |  |  |  |  |
| **1** | **1** | **0** | **0** | 42.00 z | 19.26 u | 9.33 r | 16.60 t | 4.16 r | 7.26 k-n | 76.86 fgh | 2.51 jkl | 15.74 jkl | 6.13 gh | 86.24 f | 221.24 gh |
|  |  |  | **1** | 50.33 w | 23.66 t | 11.33 pq | 17.94 s | 5.80 q | 7.03 l-o | 77.48 efg | 2.84 ghi | 17.75 ghi | 6.86 de | 96.57 ef | 249.08 f |
|  |  |  | **2** | 61.66 lmn | 29.66 qr | 14.33 m | 21.84 op | 7.60 nop | 6.53 m-q | 79.18 cde | 3.44 ef | 21.51 ef | 7.66 c | 117.13 d | 328.62 d |
|  |  | **1** | **0** | 53.00 v | 23.33 t | 11.66 p | 18.30 rs | 5.93 q | 6.86 l-p | 77.96 def | 2.79 hij | 17.45 hij | 6.83 def | 102.58 e | 261.29 f |
|  |  |  | **1** | 56.66 rst | 27.66 s | 13.16 no | 20.10 q | 6.90 p | 6.23 o-r | 80.500 bc | 3.54 c-f | 22.16 c-f | 7.86 bc | 126.98 cd | 357.66 c |
|  |  |  | **2** | 65.66 gh | 33.66 op | 16.33 l | 24.30 m | 8.86 l | 5.96 qr | 81.62 ab | 3.81 bc | 23.82 bc | 8.33 ab | 140.38 b | 399.87 b |
|  |  | **2** | **0** | 62.00 klm | 30.33 q | 14.33 m | 22.80 no | 7.80 no | 6.43 n-q | 79.46 cd | 3.46 def | 21.68 def | 7.63 c | 123.96 cd | 352.05 c |
|  |  |  | **1** | 68.00 ef | 33.66 op | 17.20 kl | 24.90 lm | 8.93 kl | 5.93 qr | 81.39 b | 3.77 bc | 23.57 bc | 8.46 a | 153.57 a | 427.76 a |
|  |  |  | **2** | 76.33 b | 38.66 kl | 20.66 hi | 30.00 hi | 11.33 h | 5.43 r | 83.33 a | 2.50 kl | 15.64 kl | 6.73 ef | 131.63 bd | 305.47 e |
|  | **2** | **0** | **0** | 59.66 nop | 32.33 p | 14.33 m | 26.00 jk | 6.93 p | 12.66 bc | 43.33 st | 3.08 g | 19.28 g | 5.03 jk | 34.03 n-q | 130.32 mn |
|  |  |  | **1** | 64.00 hij | 36.66 n | 17.66 jk | 29.33 i | 8.36 lmn | 11.66 de | 45.93 r | 3.43 f | 21.45 f | 5.66 hi | 39.74 m-p | 150.22 klm |
|  |  |  | **2** | 69.33 de | 43.00 i | 21.66 h | 34.00 f | 11.00 hi | 10.33 g | 48.53 q | 3.72 b-e | 23.30 b-e | 6.26 fg | 48.94 j-m | 181.84 ij |
|  |  | **1** | **0** | 64.66 hi | 36.00 n | 18.33 j | 30.00 hi | 8.66 l | 11.20 efg | 45.93 r | 3.44 ef | 21.54 ef | 6.00 gh | 42.13 lmn | 151.25 kl |
|  |  |  | **1** | 67.00 fg | 40.00 jk | 20.33 i | 32.33 g | 10.33 ij | 10.66 fg | 52.00 p | 3.87 b | 24.22 b | 6.86 de | 59.75 hi | 210.41 h |
|  |  |  | **2** | 74.66 bc | 46.00 h | 25.33 de | 37.33 d | 13.66 ef | 9.33 h | 56.33 o | 4.24 a | 26.51 a | 7.33 cd | 72.29 g | 260.90 f |
|  |  | **2** | **0** | 70.33 d | 43.66 i | 21.66 h | 34.66 ef | 11.33 h | 10.33 g | 48.96 q | 3.75 bcd | 23.47 bcd | 6.33 efg | 50.60 i-l | 187.51 i |
|  |  |  | **1** | 75.33 b | 48.33 g | 25.66 d | 39.33 c | 14.66 d | 9.33 h | 56.33 o | 4.26 a | 26.67 a | 7.40 cd | 66.83 gh | 240.95 fg |
|  |  |  | **2** | 81.00 a | 57.33 c | 31.33 b | 46.66 a | 17.66 bc | 8.33 ij | 59.80 n | 2.90 gh | 18.17 gh | 5.66 hi | 59.06 hij | 189.31 i |
| **2** | **1** | **0** | **0** | 37.80 a | 23.50 t | 10.54 q | 14.60 u | 5.16 q | 8.66 hi | 67.25 m | 1.31 q | 8.22 q | 4.40 lmn | 36.30 nop | 67.71 stu |
|  |  |  | **1** | 45.30 y | 28.87 rs | 14.14 mn | 16.10 t | 7.84 mno | 8.00 ijk | 69.33 l | 1.54 pq | 9.65 pq | 4.76 jkl | 41.93 l-o | 85.20 rs |
|  |  |  | **2** | 55.50 tu | 38.19 lm | 16.19 l | 17.68 s | 8.95 kl | 7.66 jkl | 72.10 j | 1.79 nop | 11.24 nop | 5.00 jk | 48.21 klm | 108.34 opq |
|  |  | **1** | **0** | 47.70 x | 29.52 qr | 12.18 op | 15.78 t | 7.35 op | 8.13 ijk | 70.02 kl | 1.54 pq | 9.68 pq | 4.75 jkl | 43.30 lmn | 88.09 qrs |
|  |  |  | **1** | 51.00 w | 34.42 o | 14.87 m | 19.21 qr | 9.02 kl | 7.33 klm | 73.49 ij | 1.87 no | 11.73 no | 5.10 ij | 51.50 i-l | 118.44 nop |
|  |  |  | **2** | 59.10 opq | 41.07 j | 18.45 j | 21.38 p | 11.32 h | 6.66 m-q | 75.22 hi | 2.28 lm | 14.26 lm | 5.90 gh | 67.30 gh | 162.82 jk |
|  |  | **2** | **0** | 55.80 st | 37.00 mn | 17.53 jk | 19.39 q | 9.67 jk | 7.00 l-p | 71.76 jk | 1.80 nop | 11.28 nop | 5.01 jk | 48.03 klm | 108.07 opq |
|  |  |  | **1** | 61.20 lmn | 41.07 j | 20.14 i | 22.57 no | 11.07 hi | 6.66 m-q | 75.92 gh | 2.28 lm | 14.25 lm | 5.91 gh | 66.19 gh | 159.58 k |
|  |  |  | **2** | 68.70 def | 48.17 g | 24.02 fg | 26.40 j | 14.05 de | 6.13 pqr | 79.38 cd | 1.67 op | 10.48 op | 4.63 j-m | 58.35 h-k | 132.12 lmn |
|  | **2** | **0** | **0** | 53.70 uv | 39.44 kl | 16.19 l | 22.88 n | 8.59 lm | 14.33 a | 40.88 u | 1.74 op | 10.90 op | 3.24 q | 15.43 s | 51.91 u |
|  |  |  | **1** | 57.60 qrs | 46.73 h | 19.96 i | 26.81 j | 10.88 hi | 13.50 ab | 42.71 t | 1.95 no | 12.20 no | 3.48 opq | 17.67 s | 61.83 u |
|  |  |  | **2** | 62.40 jkl | 52.46 e | 24.48 ef | 29.25 i | 13.80 e | 12.33 cd | 45.01 rs | 2.32 kl | 14.54 kl | 3.90 nop | 22.33 rs | 83.42 rst |
|  |  | **1** | **0** | 58.20 pqr | 43.92 i | 20.71 hi | 25.40 kl | 10.72 hi | 13.16 bc | 43.63 st | 2.03 mn | 12.70 mn | 3.40 pq | 17.39 s | 64.59 tu |
|  |  |  | **1** | 60.30 mno | 50.34 f | 22.97 g | 29.78 hi | 12.81 g | 12.33 cd | 45.47 r | 2.60 ijk | 16.25 jkl | 4.08 mn | 25.16 qrs | 100.12 pqr |
|  |  |  | **2** | 67.20 fg | 56.12 c | 28.62 c | 32.85 g | 16.94 c | 11.33 ef | 47.77 q | 2.94 gh | 18.38 gh | 4.46 k-n | 30.50 pqr | 125.97 no |
|  |  | **2** | **0** | 63.30 ijk | 54.27 d | 24.48 ef | 30.50 h | 12.96 fg | 12.33 cd | 45.93 r | 2.42 kl | 15.12 kl | 4.07 mno | 24.34 qrs | 90.72 qr |
|  |  |  | **1** | 67.80 ef | 59.52 b | 29.00 c | 35.28 e | 18.18 b | 11.00 efg | 47.77 q | 3.05 gh | 19.07 gh | 4.58 j-m | 31.64 o-r | 131.57 lmn |
|  |  |  | **2** | 72.90 c | 69.94 a | 35.40 a | 41.06 b | 22.57 a | 9.33 h | 52.36 p | 1.90 no | 11.87 no | 3.88 nop | 29.87 pqr | 91.29 qr |

**PH:** Plant height, **NMB:** Number of main branches, **NLB:** Number of lateral branches, **NSP:** Number of seeds per plant, **NPP:** Number of pods per plant, **PWP:** Pod weight per plant, **W100S:** 100-seed weight, **W100P:** 100-pod weight, **SSY:** Straw and stubble yield, **HI:** Harvest index, **MBD:** Main branch diameter, **PSP:** Percentage of seedless pods, **GY:** Grain Yield, **SC**: Stomatal Conductance, **Chl a:** Chlorophyll a, **Chl b:** Chlorophyll b, **Tot Chl:** Total chlorophyll, **Cart:** Carotenoid, **MDA**: Malondialdehyde, **EL**: Electrolyte leakage, **CAT:** Catalase, **SOD:** Superoxide dismutase, **GPX:** Guaiacol peroxidase, **APX:** Ascorbate peroxidase, **PPO:** Polyphenol oxidase, **Tot Pro**: Total soluble protein, **Prol**: Proline, **Flavo**: Flavonoid, **Phe**: Phenol, **Sug**: Sugar, **RW**C: Relative water content, **%N**: Seed nitrogen, **%Pro**: Seed protein, **%Oil:** Seed oil, **SOY:** Seed oil yield, **SPY:** Seed protein yield.

Mean comparison was performed by DUNCAN method at 5% probability. Columns with similar letters did not differ significantly

**The coding for PCA-biplot analysis:**

library(factoextra)

data<-read.table("Y1.txt", header = TRUE, sep="\t")

res.pca <- prcomp(data, scale = TRUE)

fviz_pca_var(res.pca, col.var="contrib",

gradient.cols = c("#00AFBB", "#E7B800", "#FC4E07"),

repel = FALSE # Avoid text overlapping

)

fviz_pca_ind(res.pca, col.ind = "cos2",

gradient.cols = c("#00AFBB", "#E7B800", "#FC4E07"),

repel = FALSE # Avoid text overlapping (slow if many points)

)
